# Environmental Mixture Toxicity of Guanitoxin and Organophosphates in Zebrafish: from Developmental and Neurobehavioral Phenotypes to Transcriptomic Responses

**DOI:** 10.1021/acs.est.5c16673

**Published:** 2026-03-11

**Authors:** Larissa Souza Passos, Melissa von Wyl, Elisabeth M.-L. Janssen, David Lopez-Rodriguez, Ernani Pinto, Colette vom Berg

**Affiliations:** † Laboratory of Environmental Toxicology, Center for Nuclear Energy in Agriculture, University of São Paulo, Piracicaba, São Paulo 13416-000, Brazil; ‡ Department of Environmental Toxicology, 28499 Swiss Federal Institute of Aquatic Science and Technology, Dübendorf 8600, Switzerland; § Department of Environmental Chemistry, Swiss Federal Institute of Aquatic Science and Technology, Dübendorf 8600, Switzerland; ∥ Department of Biomedical Sciences, University of Lausanne, Lausanne 1005, Switzerland; ⊥ Institute of Earth Surface Dynamics, University of Lausanne, Lausanne 1015, Switzerland

**Keywords:** cardiotoxicity, cyanobacteria, cyanotoxins, ecotoxicology, pesticides

## Abstract

Guanitoxin, a neurotoxin produced by cyanobacteria and the only known naturally occurring organophosphate, poses environmental risks comparable to synthetic organophosphate insecticides. Its effects on aquatic organisms, particularly in combination with compounds sharing similar mechanisms of action, remain poorly understood. This study evaluated the developmental toxicity of guanitoxin, alone and combined with the organophosphate insecticides malathion and trichlorfon, in zebrafish (*Danio rerio*) embryos and larvae at environmentally relevant concentrations. Developmental, neurobehavioral, and molecular endpoints were assessed. All compounds exhibited dose-dependent toxicity, with mixtures significantly enhancing both lethal and sublethal effects. The trichlorfon + guanitoxin combination was particularly potent, causing embryo coagulation, cardiac arrest, hatching failure, and pronounced pericardial swelling. Sublethal effects were consistently observed across mixtures, including edema, craniofacial malformations, muscle disorganization, and strong hypoactivity. Transcriptomic profiling revealed downregulation of genes critical for muscle contraction, synaptic signaling, energy metabolism and oxidative stress. The highest number of differentially expressed genes was observed in the trichlorfon + guanitoxin mixture (1348 genes). Synergistic interactions were observed, with mixture-induced effects exceeding those of individual compounds. Together, these findings demonstrate that guanitoxin, particularly in combination with synthetic organophosphates, poses a significant ecological risk and highlights the importance of considering mixture toxicity in environmental risk assessments.

## Graphical abstract

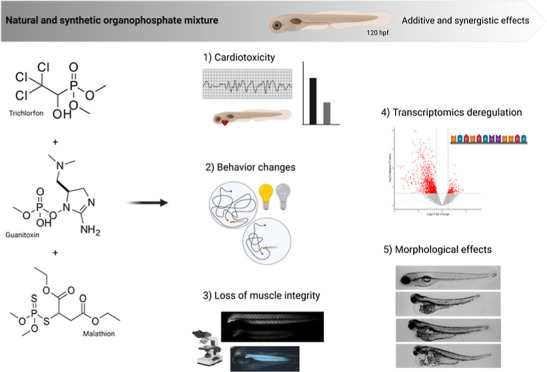

## 1 Introduction

Cyanobacteria are photosynthetic prokaryotes with fossil records dating back 3.8 billion years, representing one of the most abundant and evolutionarily significant groups of organisms on Earth, with approximately 170 genera identified to date.^1^ They play a crucial role in ecological processes and have applications in pharmaceuticals and the food industry.^2^ However, eutrophication driven by anthropogenic nutrient enrichment often promotes excessive cyanobacterial blooms, known as cyanobacterial harmful algal blooms (cyanoHABs), which produce toxic secondary metabolites.^3^

CyanoHABs lead to increased concentrations of cyanotoxins in aquatic environments.^4^ While toxins such as microcystins and saxitoxins are well documented, many cyanotoxins remain understudied with respect to their toxicological profiles.^5^ Guanitoxin is a potent neurotoxin and the only naturally occurring organophosphate identified to date.^6^ Guanitoxin irreversibly inhibits acetylcholinesterase (AChE), resulting in excessive cholinergic stimulation that can cause respiratory paralysis, convulsions, and death.^7^ Fish kills, bird poisonings, and mammalian fatalities have been linked to blooms producing guanitoxin, underscoring its significant environmental and ecological risks.^8^ Guanitoxin has been detected in freshwater ecosystems across North and South America, Europe, and Asia, primarily in habitats dominated by cyanobacteria of the genera *Dolichospermum* and *Sphaerospermopsis*.^9,10^

Despite its high toxicity, research on guanitoxin remains limited due to its chemical instability and the lack of standardized analytical methods.^11,12^ Bloom-associated animal mortality events consistent with guanitoxin exposure suggest that this cyanotoxin may represent an under-recognized threat in global watersheds.^13^ However, toxicological data on guanitoxin are scarce, despite it being one of the most potent cyanotoxins reported, with lethality comparable to that of saxitoxin.^13^ This lack of information hampers the inclusion of guanitoxin in governmental environmental monitoring programs.

Since guanitoxin shares a mechanism of action with synthetic organophosphate insecticides such as malathion and trichlorfon, their co-occurrence in aquatic ecosystems could potentiate toxic effects. Although direct evidence documenting the simultaneous presence of guanitoxin with these insecticides in aquatic environments is currently limited, their widespread use and detection, combined with the increasing frequency of cyanobacterial blooms, highlight the relevance of investigating their combined toxicity. Absence of documented co-occurrence most likely reflects analytical and monitoring limitations rather than the absence of real-world coexposure. Cyanobacterial blooms often coincide with pesticide pollution in freshwater environments due to runoff, leaching, and spray drift, particularly in agricultural and aquaculture settings.^14^ Trichlorfon is widely used in fish farming to control parasites,^15^ while malathion is broadly applied in agriculture and aquaculture, with documented toxicity to nontarget aquatic species.^16^ Toxicological studies have demonstrated adverse effects of these insecticides on various aquatic organisms, including fish and crustaceans.^17^ Despite increasing evidence of such coexposure scenarios, current environmental risk assessments often overlook the combined toxicity of natural cyanotoxins and synthetic insecticides, especially regarding early life stages of aquatic organisms.

Considering the environmental relevance of coexposure scenarios, this study investigates the developmental toxicity of guanitoxin alone and in combination with malathion and trichlorfon using zebrafish larvae. The zebrafish serves as a well-established vertebrate model organism in developmental and neurobehavioral toxicology characterized by high sensitivity to sublethal and developmental disruptions and by the availability of molecular and genomic resources for mechanistic investigations.^18^ The exposure concentrations were selected to represent environmentally relevant contamination levels and to maintain ecological relevance by simulating environmentally plausible and precautionary coexposure scenarios. These scenarios are supported by the shared mechanism of action of guanitoxin and organophosphate insecticides, the widespread use of these compounds, and the increasing frequency of cyanobacterial blooms.^19^ Although such combined exposures have not yet been captured by environmental monitoring programs, their co-occurrence in freshwater systems is environmentally plausible and likely underrepresented due to the fragmentation of pesticide and cyanotoxin surveillance and historical analytical limitations for guanitoxin. Multiple endpoints were assessed, including morphological abnormalities, cardiac function, skeletal muscle integrity, behavioral alterations, and transcriptomic changes, to characterize potential synergistic effects. The integration of morphological, behavioral, physiological, and transcriptomic biomarkers allows a comprehensive evaluation of mixture toxicity.

We hypothesize that guanitoxin and synthetic organophosphate insecticides interact through their shared mechanism of action as acetylcholinesterase inhibitors, leading to additive or synergistic disruption of cholinergic signaling. This interaction is expected to impair neuromuscular function, alter neurobehavioral development, and trigger downstream effects on energy metabolism and muscle integrity, thereby exacerbating developmental toxicity in aquatic organisms.

## 2 Material and Methods

### 2.1 Chemicals and Cyanobacterial Cultures

Methanol and acetonitrile (Optima LC/MS, 99.9%) were purchased from Thermo Scientific. Formic acid (98–100%) and absolute ethanol (EMSURE ACS ISO Reag. Ph Eur) were supplied by Sigma-Aldrich. All salts used in the preparation of culture media were of analytical grade. The insecticides malathion (99% purity) and trichlorfon (98% purity) were also sourced from Sigma-Aldrich (USA). Ultrapure water was obtained using a NANOpure 21 purification system (Barnstead, Thermo Scientific).

Because a purified guanitoxin analytical standard is not commercially available and its quantitative determination as an isolated compound was not feasible under our experimental conditions, exposures were conducted using crude extracts from *Sphaerospermopsis torques-reginae* (ITEP-024 strain), a known guanitoxin-producing strain. Cultures were grown in ASM-1 medium and maintained at 22 ± 0.5 °C under a light intensity of 20 μmol photons m^–2^ s^–1^ with a 12:12 h light–dark cycle. After cultivation, cell suspensions were concentrated and lyophilized. For toxicity testing, the biomass was subjected to aqueous extraction following the cell lysis and guanitoxin release protocol described by Passos et al.^20^ Accordingly, all concentrations reported throughout the manuscript as “guanitoxin” refer to crude extract–equivalent concentrations (i.e., mg crude extract per L), rather than analytically quantified concentrations of purified guanitoxin. This extract-equivalent exposure approach is consistent with our previous work conducted under the same analytical constraints (see^6,20^ ).

### 2.2 Zebrafish Maintenance and Embryo-Larval Experimental Procedures

Zebrafish (*Danio rerio*, WM strain with a mixed wild-type genotype) were kept at the Swiss Federal Institute of Aquatic Science and Technology (Eawag, Switzerland) in accordance with Swiss animal welfare regulations under the license number ZH166/0000 approved through the Cantonal Veterinary office Zürich. Since the experimental procedures only involved zebrafish larvae up to 5 days post fertilization, ethical approval was not required under Swiss regulations. The WM (wildtype mix) zebrafish strain was selected for this study due to its stable reproductive performance, increased genetic diversity relative to inbred laboratory strains, and demonstrated sensitivity to organophosphate insecticides, as confirmed by both internal experiments and single-compound dose–response testing in this study (Figure 1). Adults were maintained in 12 L tanks within a recirculating water system at 26 °C, under a 14:10 h light–dark cycle. For egg collection, spawning trays were introduced at the end of the day, and fertilized eggs were retrieved approximately 1 h postfertilization the following morning. Collected embryos were rinsed and transferred to aerated embryo medium prepared with nanopure water containing CaCl_2_·2H_2_O, MgSO_4_·7H_2_O, NaHCO_3_, and KCl, according to ISO 7346-3:1996 guidelines.

**Figure 1:**
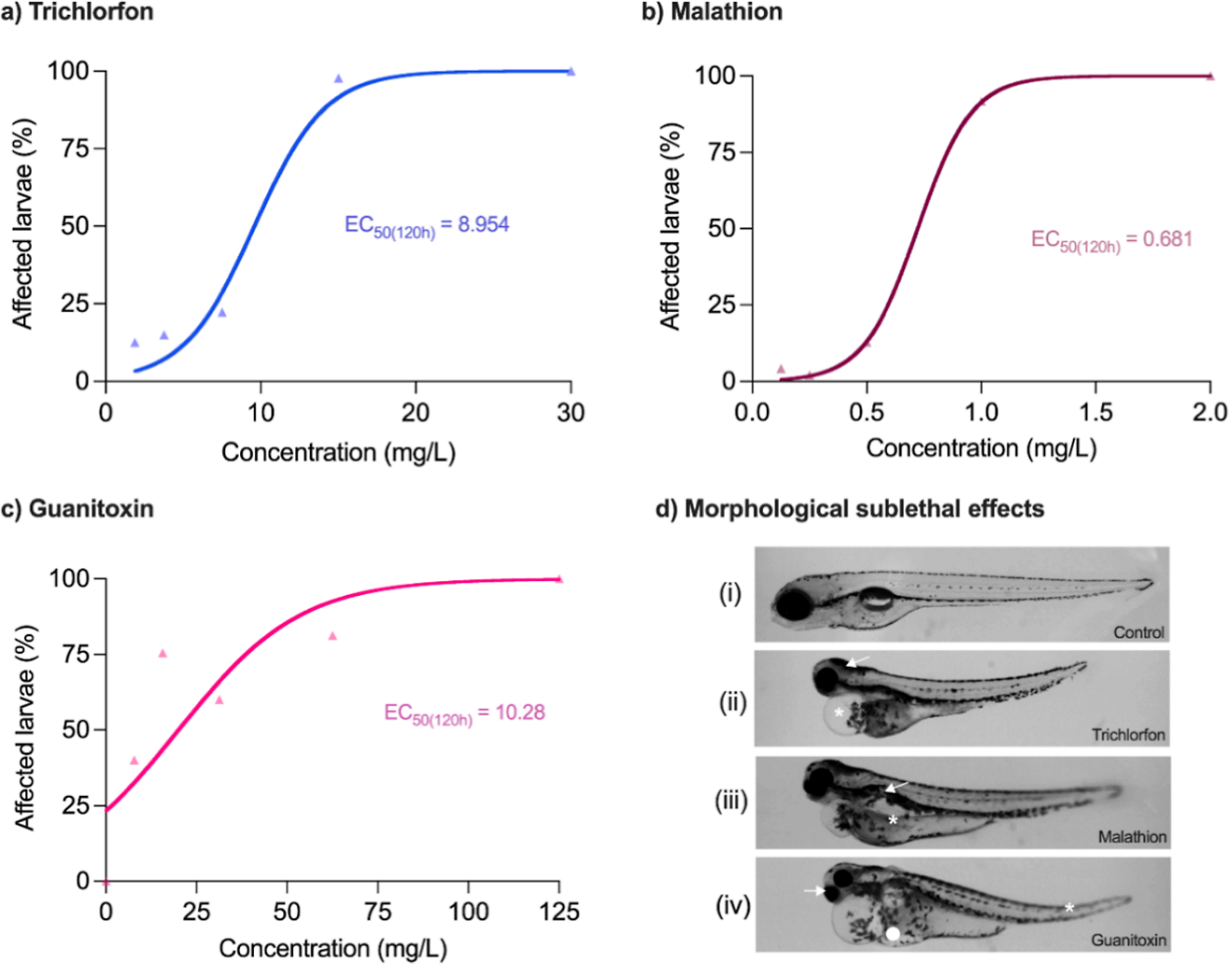
Concentration–response relationships and effective concentration affecting 50% of zebrafish larvae after 120 h postfertilization (EC_50_) for trichlorfon (A), malathion (B), and guanitoxin (C). Panel D illustrates sublethal effects through morphological and morphometric abnormalities: (i) normal morphology (control); (ii) head malformation (arrow), cardiac edema (asterisk); (iii) absence of swim bladder inflation (arrow); yolk sac edema (asterisk); (iv) reduced eye size (arrow), spinal curvature (asterisk), and yolk sac deformation (circle).

For each experiment, stock solutions of guanitoxin-containing extracts, trichlorfon, and malathion were freshly prepared by dissolving the compounds in embryo medium, and final concentrations were achieved by serial dilution in the same medium. Fertilized eggs were pre-exposed to the chemical solutions in Petri dishes, and only fertilized eggs were selected and transferred to exposure plates (48-well plates) for the Fish Embryo Toxicity (FET) tests.^21^ Additional experiments for transcriptomic analysis were conducted under the same conditions in large Petri dishes. The toxicity of guanitoxin, trichlorfon, and malathion was tested both individually and in various binary and tertiary mixtures, with exposure lasting 120 h postfertilization (hpf). Due to the short half-life of the organophosphates, the exposure was nonstatic, with medium changes every 48 h. The chemical characteristics of the three compounds are presented in Table S1.

The concentrations used in the individual experiments were determined based on literature-reported EC_50_ values for guanitoxin (7.81–125 mg/L; T1–T5), trichlorfon (1.875–30 mg/L; T1–T5), and malathion (0.125–2.0 mg/L; T1–T5), while maintaining environmentally plausible and precautionary coexposure scenarios. T1 to T5 correspond to the treatment concentrations from the lowest (T1) to the highest (T5). Detailed information on each treatment concentration is provided in the Supporting Information (Appendix 1).

Guanitoxin concentrations were based on levels associated with cyanobacterial blooms, corresponding to cell densities of 10^6^–10^7^ cells/mL (≈250 mg/L crude extract).^22^ The reported guanitoxin concentrations refer to the aqueous crude extract of *S. torques-reginae* (ITEP-024 strain) producing guanitoxin, as described in Section 2.1. Accordingly, a concentration of 125 mg/L corresponds to 125 mg of crude extract per liter of water. Malathion concentrations were selected according to reported levels in agricultural runoff that may exceed drinking water guidelines (e.g., ∼0.8 mg/L).^23^ Trichlorfon concentrations were chosen based on residues reported in aquaculture systems, where recommended doses (0.1–1 mg/L) are frequently exceeded, with documented applications reaching up to 300 mg/L at low temperatures and 15 mg/L at moderate temperatures due to improper use.^19^

For each organophosphate, the exposures were performed in 3 independent replicates with 16 fish per concentration and replicate, resulting in a total of 48 fish per concentration, including the negative control (embryo medium). A positive control (4 mg/L of 3,4-dichloroaniline) was also included (*n* = 42). The sample size is in accordance with OECD Test Guideline 236.

For the binary mixture experiments, concentrations were defined based on the EC_50_ values obtained from the individual compound tests. A concentration-ratio approach was applied to systematically assess mixture effects across different exposure scenarios, including six equitoxic and four nonequitoxic ratios, resulting in ten distinct binary combinations (Table 1). This design allowed evaluation of both balanced and unbalanced contributions of each compound to mixture toxicity with equitoxic mixtures enabling mechanistic evaluation under equal toxic pressure, irrespective of differences in potencies, while nonequitoxic mixtures representing environmentally realistic scenarios in which individual components contribute unevenly to overall toxicity.

**Table 1:** Ratios Used in the Binary Mixture Experiments with Guanitoxin-Containing Extracts, Malathion, and Trichlorfon Based on the EC_50(120h)_ Values from the Individual Experiments

combination	EC_50(120h)_ ratios	GNT + MLT (mg/L)	MLT + TCF (mg/L)	TCF + GNT (mg/L)
C1	0.5 + 0.25	5.14 + 0.17	0.34 + 2.24	4.48 + 2.57
C2	0.25 + 0.5	2.57 + 0.34	0.17 + 4.48	2.24 + 5.14
C3	0.5 + 1	5.14 + 0.68	0.34 + 8.95	4.48 + 10.28
C4	1 + 0.5	10.28 + 0.34	0.68 + 4.48	8.95 + 5.14
C5	0.5 + 1.5	5.14 + 1.02	0.34 + 13.43	4.48 + 15.42
C6	1.5 + 0.5	15.42 + 0.34	1.02 + 4.48	13.43 + 5.14
C7	0.5 + 0.5	5.14 + 0.34	0.34 + 4.48	4.48 + 5.14
C8	1 + 1	10.28 + 0.68	0.68 + 8.95	8.95 + 10.28
C9	1.5 + 1.5	15.42 + 1.02	1.02 + 13.43	13.43 + 15.42
C10	2 + 2	20.56 + 1.36	1.36 + 17.91	17.91 + 20.56

Tertiary mixture concentrations were subsequently selected based on the outcomes of the binary mixture’s experiments, with three equitoxic and three nonequitoxic ratios used (Table 2). Each binary and tertiary combination was tested in a single experiment, with a sample size of 20 embryos per combination, 32 embryos for the negative control, and 42 for the positive control, in accordance with OECD Test Guideline 236. In all exposures, the final volume in each well of the microplate was 500 μL.

**Table 2:** Ratios Used in the Tertiary Mixture Experiments with Guanitoxin-Containing Extracts, Malathion, and Trichlorfon Based on the Results from the Binary Experiments

combination	EC_50(120h)_ ratios	TCF + GNT + MLT (mg/L)
C1	0.1 + 0.1 + 0.5	0.90 + 1.03 + 0.34
C2	0.1 + 0.5 + 0.1	0.90 + 5.14 + 0.07
C3	0.5 + 0.1 + 0.1	4.48 + 1.03 + 0.07
C4	0.1 + 0.1 + 0.1	0.90 + 1.03 + 0.07
C5	0.25 + 0.25 + 0.25	2.24 + 2.57 + 0.17
C6	0.5 + 0.5 + 0.5	4.48 + 5.14 + 0.34

For all experiments, physicochemical parameters, including temperature, dissolved oxygen, and pH, were measured in the negative and positive controls, as well as in the highest concentration treatments at the start of the experiment. Water samples were collected at 0, 48, 96, and 120 h for chemical analysis (see Section 2.3). Daily observations of lethal and sublethal effects were conducted following OECD guideline 236 ^21^ and Kimmel et al.,^24^ using a stereo microscope (Leica S8APO). Cardiovascular, morphological, and behavioral assessments were performed on the final day after evaluating lethal and sublethal endpoints. Additionally, analyses of skeletal muscle integrity and transcriptomic profiling were carried out. All assessments were conducted by a single trained observer using standardized criteria to minimize interobserver variability.

### 2.3 Chemical Analysis

High-performance liquid chromatography coupled with mass spectrometry (LC–MS/MS) was used to detect guanitoxin, while HPLC coupled with diode-array detection (DAD) was employed to quantify malathion and trichlorfon and assess their stability. For this purpose, samples were collected and analyzed at 0, 48, 96, and 120 h of exposure (*n* = 3 per concentration). Detailed methods used for guanitoxin and insecticide analyses are presented in the Supporting Information (Appendix 1).

### 2.4 Heart Rate and Morphological Measurements

Heart rate and morphological measurements of all exposed larvae, including the control group, were performed on the last day of the experiment, at 120 hpf. The larvae were anesthetized with ethyl 3-aminobenzoate methanesulfonate (160 mg/L). A 30 s video of each larva in the lateral position was recorded at 30 frames per second (Media Recorder 4 software, version 4.0; Noldus, Netherlands) using a Basler acA2000–165 μM camera installed in a stereo microscope (Leica S8APO). The acquired videos were analyzed in the DanioScope Software (version 1.2.206; Noldus, Netherlands). The software calculates the number of heart beats per minute. Body length, eye size, pericardial area, yolk area, and swim bladder area were manually delimited in the images and measured using the software.

### 2.5 Behavior Tracking

For analysis of larval behavior, we used the DanioVision Observation Chamber (v. DVOC-0040T; Noldus, Netherlands). This equipment consists of a Gigabit Ethernet video camera with infrared and white light sources and a transparent multiwell cardholder. A standard PC system with EthoVision XT13 software (version 13.0.1220, Noldus, Netherlands) connected to the camera recorded videos for further analysis of locomotor activity. The behavioral analysis consists of six phases, the acclimation period being 20 min to light to allow the fish to adjust (I); spontaneous swimming behavior (II); two alternating 10 min dark intervals (III and V); and two alternating 10 min light intervals (IV and VI). The recorded videos make it possible to track the movement of the fish in the EthoVision XT13 software. To minimize potential handling effects, all larvae were handled identically across treatments and were allowed a 20 min acclimation period inside the observation chamber prior to the start of behavioral recordings.

The effect size for each treatment of behavior mixtures in dark phases, compared to the control group, was calculated using Cohen’s d and the common language effect size (CLES), with the aid of the online tool available at Psychometrica (https://www.psychometrica.de/effect_size.html). Cohen’s d was interpreted according to conventional benchmarks: small (0.2), medium (0.5), large (0.8), and very large (1.3).

### 2.6 Assessment of Skeletal Muscles

The integrity of the skeletal muscles of zebrafish was assessed using the birefringence measurement method adapted from Smith et al.^25^ To achieve this, following morphological, behavioral, and heart rate analyses, all larvae were fixed. Larvae were immersed in a solution of 4% PFA (paraformaldehyde) + 100× Triton and left on a shaker overnight. After this period, the PFA solution was replaced with 1× PBS (phosphate-buffered saline) and shaken for 10 min. Subsequently, the solution was changed again, and a mixture of 1:1 PBS: glycerol was added, shaken for 1 h. Finally, 100% glycerol was added, and the samples were refrigerated at 4 °C until microscope analysis.

The measurement evaluates a physical effect resulting from the diffraction of polarized light through muscle sarcomeres.^26^ A Leica S8APO stereo microscope with a Basler acA2000-165 μm camera and additionally equipped with two linear polarizing sheets (400–700 nm, Thorlabs, USA) was used. For imaging, a single larva was placed laterally on the under leaf and positioned to maximize the brightness of the birefringence signal. Images of 15 larvae per treatment were acquired on the same day with the same magnification and light and camera settings using pylon video recording software (Basler AG, DE). The images were analyzed with the FIJI software using the polygon selection tool to select the trunk musculature and measure the average signal intensity. In addition, the angle between the dorsal and ventral muscle Hemisegments were evaluated using the FIJI angle tool. Somite angles were measured in the trunk region above the bud extension. From three independent measurements per fish, an average angle was calculated.

### 2.7 Transcriptomics Assessment

Transcriptomic analyses were performed using a binary (C7) and a tertiary (C6) mixture, both corresponding to 0.5 × EC_50_. This concentration was chosen because it represents the lowest equitoxic concentration among the compounds tested. This allowed us to compare transcriptomic responses across treatments while minimizing secondary effects associated with higher toxicity.

Guanitoxin-containing extracts (T5:125 mg/L), malathion (T4:1 mg/L), and trichlorfon (T4:15 mg/L) were also tested individually. The binary combination was selected based on equitoxicity, and among the equitoxic mixtures, the one producing the least morphological damage (e.g., severe edema or deformation) and lowest mortality was chosen. Individual concentrations were defined according to the criterion of minimizing lethal effects. All analyses were conducted in triplicate, with each replicate consisting of 20 pooled larvae. RNA extraction, library construction, sequencing, and data processing are presented in the Supporting Information (Appendix 1).

### 2.8 Assessment of Mixture Effects

To determine the type of interaction in the mixtures, the total percentage of affected larvae during exposure (total affected larvae, %) in the FET assay was evaluated using the concentration addition (CA) model, which assumes additive effects of compounds with similar modes of action. Interactions were defined by comparing the observed effect with the predicted effect: synergistic when the observed effect exceeded the predicted effect, antagonistic when it was lower, and additive when it was similar.

For other biological parameters, including locomotion, muscle integrity, morphology, and cardiac endpoints, an empirical approach was applied. Interactions were classified as synergistic when the observed mixture effect exceeded the empirical sum of the individual effects, antagonistic when the observed mixture effect was substantially lower than the empirical sum (difference >10%), and additive when the observed mixture effect differed from the empirical sum by less than 10%. These deviation thresholds (±10%) were adopted to account for inherent biological variability and experimental uncertainty when distinguishing additive from synergistic or antagonistic interactions.

This dual framework enabled a comprehensive assessment of both general toxicity and sublethal biological effects of trichlorfon, malathion, and guanitoxin mixtures in zebrafish larvae.

### 2.9 Statistical Analysis

Normality of the data was evaluated using the Shapiro–Wilk test. When data met the assumptions of normality and homogeneity of variance, treatment effects were assessed using one-way analysis of variance (ANOVA), followed by Dunnett’s post hoc test for comparisons with the control group. In cases where data were nonparametric, the Kruskal–Walli’s test was applied. For concentration–response analyses, EC_50_ values (the concentration producing 50% of the maximal effect) were calculated using nonlinear regression based on a four-parameter logistic (4PL) model, as the study was designed to employ sublethal concentrations and to evaluate sublethal endpoints. This model estimates the minimum and maximum response levels, the slope (Hill coefficient), and the logarithm of the EC_50_. Goodness-of-fit was evaluated through *R* ^2^ values and residual analysis, and 95% confidence intervals for the EC_50_ were reported. A heatmap was constructed using all biomarker data, normalized relative to the control group (%). Detailed raw data are available in Tables S2 and S3. All statistical analyses were performed using GraphPad Prism version 10 (GraphPad Software, USA), and results were considered statistically significant at *p* ≤ 0.05.

## 3 Results and Discussion

Zebrafish embryos were exposed to natural and synthetic organophosphates following OECD guidelines, with chemical concentrations in the exposure media provided in Table S4. Malathion concentrations (0.125–1.0 mg/L) were selected based on levels detected in agricultural runoff, which have been reported to exceed WHO drinking water guidelines.^23,27^ Similarly, trichlorfon concentrations (1.875–30 mg/L) reflect residues found in aquaculture environments and doses commonly used for parasite control, which may reach up to 300 mg/L at low temperatures and 15 mg/L at moderate temperatures due to improper use.^19^

The concentration of guanitoxin extracts corresponds to cyanobacterial cell densities reported during blooms, ranging from 10^6^ to 10^7^ cells/mL,^2^ as well as concentrations applied in previous toxicity studies. Based on Passos et al.,^20^ an equivalent of 250 mg/L of crude extract from the ITEP-024 strain producing guanitoxin corresponds to approximately 10^6^ cells/mL, which is considered representative of a bloom. A limitation inherent to studies of guanitoxin is that exposures must be conducted using crude extracts, as the purified cyanotoxin is not commercially available and quantitative dosing is not possible. Accordingly, the effect concentrations reported here should be interpreted as extract-equivalent toxicity thresholds of the administered material.

Although cyanobacterial extracts may contain additional coextracted metabolites (e.g., natural products primarily associated with nontoxic bioactivities or photoprotective functions, such as namalides, spumigins, and mycosporine-like amino acids [MAAs]), these constituents are not generally recognized as the primary toxicants in this species. Moreover, guanitoxin is highly potent in the biological endpoints evaluated in this study, and its toxicity is expected to predominate in the observed responses. Consequently, coextracted metabolites are more plausibly secondary modifiers rather than primary drivers of the dose–response relationship. Nonetheless, we acknowledge that such constituents could theoretically influence apparent potency through additive, antagonistic, or mitigating interactions.

### 3.1 Acute Toxicity toward Zebrafish Larvae

Guanitoxin, malathion, and trichlorfon exhibited significant toxicity in zebrafish larvae, with 120 hpf EC_50_ values (derived from sublethal concentration–response relationships, as mortality was not an intended endpoint) of 8.95 mg/L (95% CI: 4.41–14.78; HillSlope: 6.48) for trichlorfon, 0.68 mg/L (0.596–0.751; HillSlope: 6.214) for malathion, and 10.28 mg/L (0.19–22.19; HillSlope: 0.90) for guanitoxin-containing extracts (Figure 1A–C). The shallow HillSlope observed for guanitoxin, in contrast to the steep slopes obtained for the synthetic organophosphate insecticides, suggests a more gradual dose–response relationship. This may reflect a combination of factors, including variability associated with the use of a cyanobacterial extract, the known chemical instability of guanitoxin, and potential differences in toxicodynamics compared with synthetic organophosphates. Additionally, inherent biological variability and limitations in slope estimation under these conditions may contribute to the observed response pattern.

These findings highlight potential ecotoxicological risks even at environmentally relevant concentrations. Environmental concentrations of trichlorfon are generally lower than the EC_50_ values reported here (0.00027–0.179 mg/L),^28–30^ but overdosing in aquaculture can reach up to 25 g/L. Trichlorfon has been shown to disrupt metabolism, neurodevelopment, immunity, oxidative balance, and induce genotoxicity.^15,31^ In line with these observations, Shi et al.^32^ demonstrated that exposure of zebrafish embryos to trichlorfon at concentrations commonly used in aquaculture (0.1–5 mg/L) resulted in reduced survival, impaired hatching and growth, increased malformations, and decreased locomotor activity. At the molecular level, trichlorfon inhibited acetylcholinesterase activity and altered cholinergic, dopaminergic, and serotonergic signaling pathways, accompanied by downregulation of genes associated with central nervous system development, highlighting its developmental neurotoxicity. To the best of our knowledge, despite these well-documented sublethal and neurodevelopmental effects, no EC_50_ values have been previously reported for zebrafish embryos or larvae exposed to trichlorfon.

On the other hand, similar concentrations have been detected for malathion in rivers (0.31–0.72 mg/L) and surface waters (0.77–0.86 mg/L) in Jalisco, Mexico.^15,33^ Moreover, chronic exposure to malathion (0.1–1 mg/L over 60 days) impairs reproductive parameters, disrupts gametogenesis, alters hormone and vitellogenin levels, and modifies gene expression in adult zebrafish.^17^ In addition, exposure to sublethal concentrations of malathion induces significant developmental alterations in zebrafish embryos (0.5–3 mg/L).^34^ Binary mixtures involving malathion also significantly altered gene expression compared to single exposures.^35^ Like trichlorfon, no EC_50_ has been reported in the literature for zebrafish embryos and larvae exposed to malathion. Thus, this study provides this important toxicological value for both insecticides.

Moreover, guanitoxin-producing blooms have caused fish kills and poisonings in birds and mammals worldwide.^8^ The EC_50_ value reported here (∼10 mg/L) is lower than the concentration of crude extract from the ITEP-024 strain already considered representative of a bloom (250 mg/L, calculated based on the number of cells in a bloom; 10^6^ cells/mL).^20^ It is important to note, as explained previously in the Materials and Methods section, that no analytical standards are currently available for guanitoxin; therefore, aqueous extracts obtained from the ITEP-024 strain were used. Guanitoxin is among the most potent naturally occurring freshwater neurotoxins. Cytotoxicity of *S. torques-reginae* ITEP-024 was observed in zebrafish hepatocytes (ZF-L), with an EC_50_ (24 h) of 366.5 mg/L.^36^ FET assays with aqueous extracts from this strain induced lethality and sublethal effects in larvae, including edema, spinal curvature, developmental delay, hatching failure, and yolk sac enlargement, with an LC_50_ (96 h) of 353.6 mg/L. The differences in EC_50_ observed in this study could be explained by the methodologies used in both studies. In Passos et al.,^36^ after extraction, samples were dried in a SpeedVac for approximately 24 h at room temperature, which may have degraded part of the toxin present in the extract, decreasing consequently the toxicity of the extract. This step was not included in our extraction method here.

### 3.2 Morphological Assessment

Lethal and sublethal abnormalities were evaluated in zebrafish larvae (Tables S5 and S6, Figure 1D). For individual exposures, guanitoxin and trichlorfon frequently caused absent heartbeat and hatching failure, while malathion mainly induced coagulation and impaired hatching. Sublethal effects were most severe with trichlorfon, where high concentrations (T5, 30 mg/L) induced widespread cardiac and yolk sac edema (>90%), yolk deformation, and impaired motility. Guanitoxin also triggered high incidences of tremors and edema at T4–T5 (62.5–125 mg/L), whereas malathion primarily caused neuromuscular symptoms, reflected in uncontrolled movements and trembling at elevated levels (T5, 2 mg/L), with other malformations being less pronounced.

Binary mixtures enhanced toxicity relative to single exposures (Table S7). Guanitoxin + malathion combinations produced 100% affected larvae in half of the tested ratios, with prominent edema and abnormal motility. The most common interaction type was additive. Malathion + trichlorfon mixtures were particularly severe, with all combinations exhibiting synergism and additivity; most combinations led to complete larval impairment, and the highest doses (C10) caused extensive craniofacial, ocular, and cardiac malformations. Guanitoxin + trichlorfon mixtures also proved highly toxic, with equitoxic ratios (C8–C9) inducing high lethality and multiple malformations. Interactions were mostly synergistic and additive, with two cases of antagonism. In tertiary mixtures (guanitoxin + malathion + trichlorfon), even moderate concentrations resulted in 100% affected larvae, dominated by neuromuscular dysfunction such as tremors and loss of coordination. Almost all combinations were synergistic.

Overall, the guanitoxin + trichlorfon combination emerged as the most lethal, with systemic developmental disruption consistent with neurotoxic effects.^37^ The widespread occurrence of tremors across treatments strongly indicates cholinergic neurotoxicity, consistent with AChE inhibition and receptor overstimulation.^38^ Similar neuromuscular phenotypes have been observed in zebrafish exposed to diisopropylfluorophosphate^39^ or methomyl,^40^ confirming the mechanistic link to neuronal hyperexcitation and degeneration. Beyond neuromuscular outcomes, frequent cardiac abnormalities and swim bladder defects were observed, which are further discussed in Section 3.3.

### 3.3 Morphometric and Cardiac Assessment

Developmental endpoints were quantified via image analysis (excluding severely deformed larvae, i.e. trichlorfon + guanitoxin C8). For individual exposures, trichlorfon markedly reduced body length, eye size, and swim bladder area while enlarging yolk sac and pericardial regions, with heart rate halved at the highest dose (Figure S1). Guanitoxin caused moderate decreases in eye and swim bladder size, with enlarged pericardial area but no effect on heartbeat (Figure S2). Malathion primarily reduced swim bladder size and induced moderate pericardial enlargement, with only slight effects on growth and cardiac frequency (Figure S3).

Binary mixtures amplified toxicity beyond additive expectations. Guanitoxin + malathion consistently reduced swim bladder size (Figure S4) and showed clear synergism in pericardial enlargement (Figure 2A, Table S10), exceeding the sum of single-exposure effects. Trichlorfon + guanitoxin exhibited the strongest synergy (Table S9), with drastic reductions in body length and eye size, major yolk sac enlargement (Figure S5), and pericardial swelling >600% (Figure 2B). Notably, this pronounced pericardial increase reflects a consistent shift in pericardial area across exposed individuals, rather than being driven by isolated extreme values, as evidenced by the distribution patterns shown in the violin plots.

**Figure 2:**
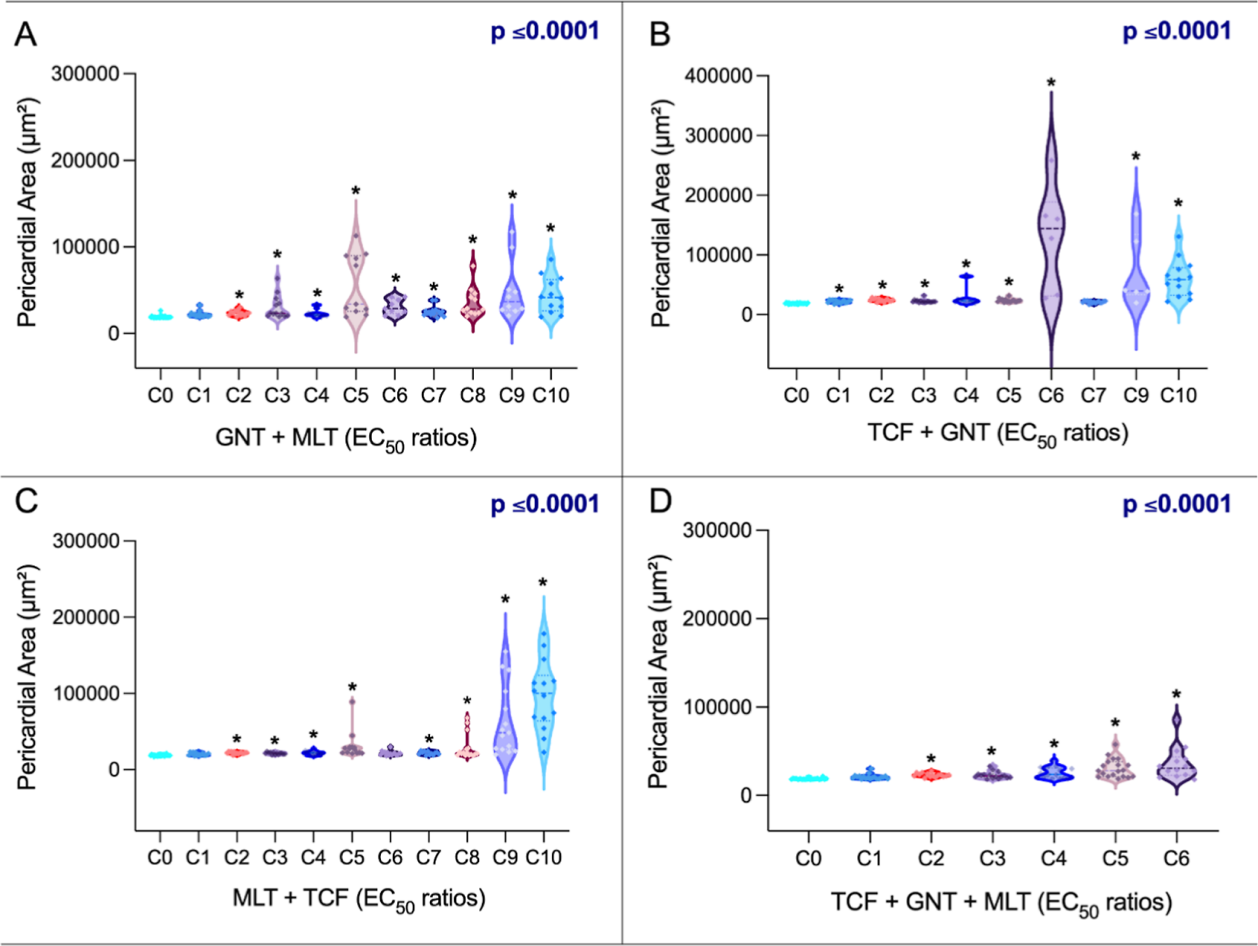
Pericardial area (μm^2^) of zebrafish larvae exposed to different combinations of guanitoxin + malathion (GNT + MLT) (A), trichlorfon + guanitoxin (TCF + GNT) (B), malathion + trichlorfon (MLT + TCF) (C), and trichlorfon + guanitoxin + malathion (TCF + GNT + MLT) (D) for 120 h. Data are presented as violin plots, showing the distribution of values with the median and quartiles indicated. Asterisks (*) indicate statistically significant differences compared to the control group at each experimental time point, based on one-way ANOVA followed by Dunnett’s post hoc test (*p* ≤ 0.05). The sample size was 20 larvae per chemical. The *p*-value for each mixture is also presented. C1 to C10 or C1 to C6 represent the different chemical mixtures (ratios based on the EC_50_, expressed in mg/L; see Tables 1 and 2).

Malathion + trichlorfon combinations also caused reductions in body length and eye size, showing synergistic effects at certain concentrations (Figure S6 and Table S11). In tertiary mixtures (trichlorfon + guanitoxin + malathion) (Figure S7), reductions in growth parameters coincided with pronounced pericardial expansion (Figure 2D).

These alterations highlight systemic developmental impairment. Sublethal cardiac defects, particularly pericardial edema and heart rate depression is consistent with AChE inhibition and muscarinic overstimulation of the sinoatrial node.^41^ Such cardiotoxic disruption during organogenesis can impair cardiovascular performance and survival later in life.^42^ In addition, reduced eye size, yolk sac enlargement, and swim bladder malformations indicate broad neurodevelopmental and metabolic disruption. Swim bladder defects prevented surface gulping, leading to uninflated bladders, compromising buoyancy and locomotion.^43^ Together, these effects are likely to reduce larval growth, viability, and physiological performance.

### 3.4 Behavioral Tracking

Locomotor activity was measured in zebrafish larvae across light/dark phases. Trichlorfon and guanitoxin consistently reduced activity during dark phases at all concentrations (T1–T5; lowest to highest), with maximal decreases of 98% (trichlorfon, 30 mg/L) and 51% (guanitoxin, 125 mg/L) (Figure S8). Very large effects were observed for guanitoxin at all concentrations using Cohen’s d and common language effect size—CLES (e.g., T5: *d* = 5.147, CLES = 1.0) (Table S7), indicating a robust effect independent of dose. Trichlorfon also induced strong, dose-dependent hypoactivity, with very large effect sizes at all concentrations (e.g., T5: *d* = 12.391, CLES = 1.0) (Table S7), reflecting pronounced locomotor disruption even at the lowest doses tested.

Malathion reduced locomotion only at the highest concentrations (T4–T5), reaching 73% at T5 (2 mg/L) in the dark phase, and exhibited a biphasic response in the light phase, with hypoactivity at low doses (T1–T2: −6–15%) and hyperactivity at high doses (T4–T5: +9–18%). Effect size analysis supported this pattern: T1 and T2 showed large negative effects and T4–T5 presented very large positive effects (Table S7). These findings indicate that malathion triggers dose-dependent alterations in locomotor activity, shifting from suppression at low to intermediate doses to pronounced hyperactivity at high doses. Thus, the biphasic pattern of malathion reflects complex interactions between inhibitory toxicity at low doses and neuronal overexcitation at high doses, which become apparent during light phases under tighter inhibitory and sensory control.

Mixture exposures generally amplified locomotor effects (Figure 3). In the malathion + trichlorfon group, most combinations exhibited synergistic reductions in locomotion (Table S11). Trichlorfon + guanitoxin mixtures were largely antagonistic (Table S9). Guanitoxin + malathion combinations showed synergism (Table S10). In tertiary mixtures (trichlorfon + guanitoxin + malathion), several combinations also exhibited reductions, highlighting the enhanced neurotoxicity of multicompound exposures. This pattern was supported by Cohen’s d effect size analysis, which revealed very large to extremely large behavioral effects across most mixture conditions in the dark phases (Cohen’s *d* > 1.3) (Table S8). In several combinations, Cohen’s d values exceeded 4, with CLES values approaching 1.0, indicating a near-certain probability that exposed larvae displayed reduced locomotor activity compared to controls. Collectively, these results demonstrate that mixture exposures markedly amplify neurobehavioral impairment relative to single-compound exposures.

**Figure 3:**
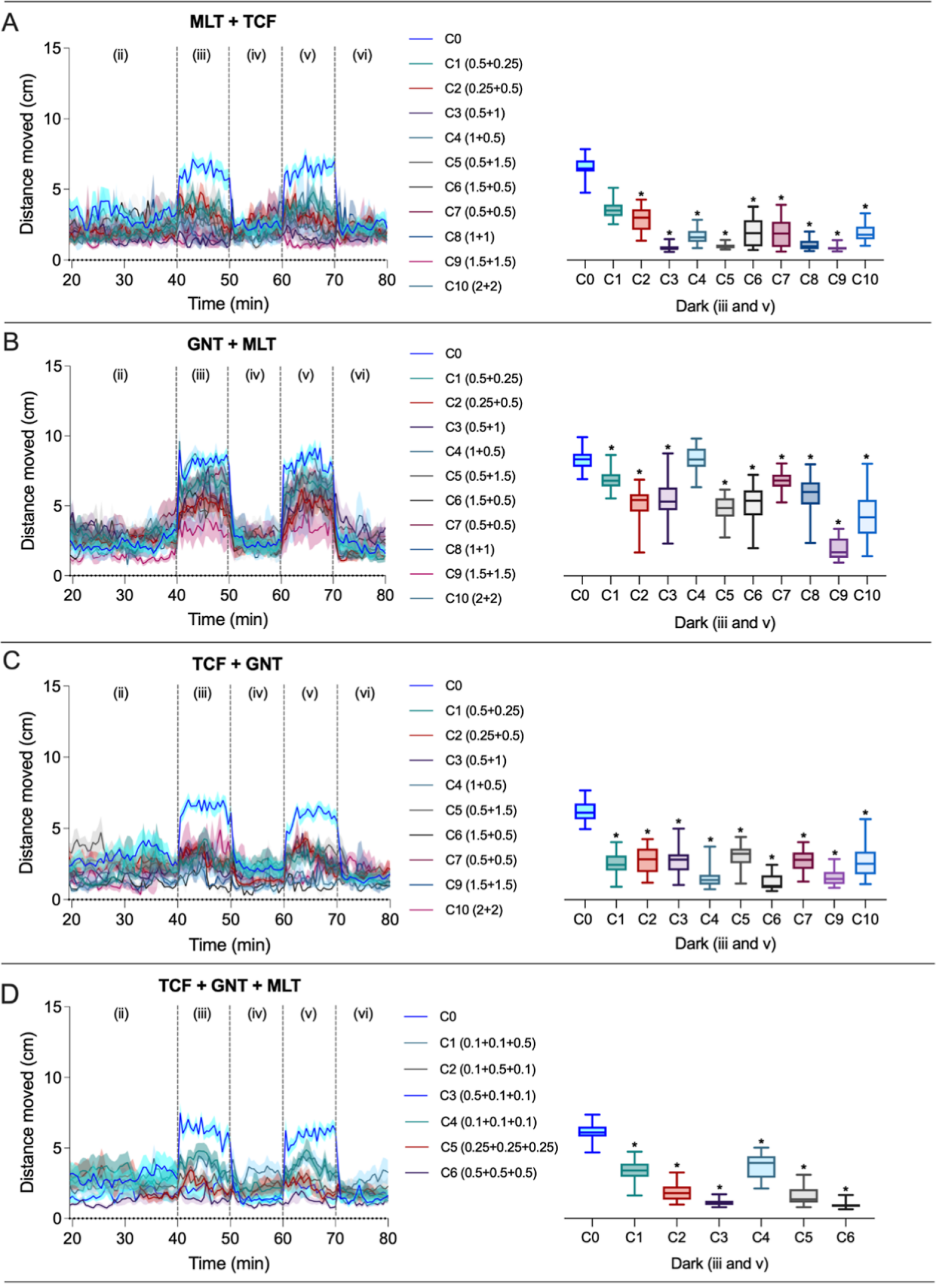
Locomotor activity of zebrafish larvae exposed to different combinations of malathion + trichlorfon (MLT + TCF) (A), guanitoxin + malathion (GNT + MLT) (B), trichlorfon + guanitoxin (TCF + GNT) (C), and trichlorfon + guanitoxin + malathion (TCF + GNT + MLT) (D) during the behavioral assay. Results are presented by stimulation phases: Phase II (spontaneous activity), Phases III and V (dark stimulation), and Phases IV and VI (light stimulation). The dark phases are also presented as boxplots, showing the median, interquartile range, and minimum/maximum values (whiskers). Asterisks (*) indicate statistically significant differences compared to the control group at each experimental time point, based on one-way ANOVA followed by Dunnett’s post hoc test (*p* ≤ 0.05). The sample size was 20 larvae per chemical. C1 to C10 or C1 to C6 represent the different chemical mixtures (ratios based on the EC_50_, expressed in mg/L; see Tables 1 and 2).

These reductions align with previous studies showing that AChE inhibitors decrease zebrafish larval motility.^40,44^ Although AChE activity was not directly assessed in the present study, the observed behavioral impairments agree with the well-established mode of action of organophosphate compounds, which act primarily through AChE inhibition. Besides, distinct behavioral profiles were observed among the compounds. Trichlorfon exposure led to unresponsiveness, likely due to neurodepression or disrupted neuromuscular transmission, consistent with reduced locomotion and AChE inhibition reported by Shi et al.^45^ In contrast, guanitoxin- and malathion-exposed larvae often exhibited uncontrolled movements, including tremors, indicative of neuroexcitation from excessive ACh accumulation at neuromuscular junctions. Malathion also exhibited a biphasic response in light phases, with hypoactivity at low doses (T1–T2) and hyperactivity at higher concentrations (T4–T5). Interestingly, this is opposite to the expected pattern of hyperactivity at low and hypoactivity at high doses, which would result from AChE inhibition and subsequent acetylcholine accumulation. Such an inversion may stem from differential sensitivity of fast and slow muscle fibers with effects becoming more apparent in the light phase where baseline locomotion is low and motor output is under tighter inhibitory control.

Overall, these neurobehavioral impairments can progress to paralysis and mortality, particularly in trichlorfon and guanitoxin exposures—that caused reduced activity even at low concentrations. These findings further underscore the importance of assessing mixture toxicity in environmental risk evaluations, as real-world exposures rarely involve single compounds.^46^

### 3.5 Assessment of Skeletal Muscles

Muscle and somite development were evaluated using birefringence as an indicator of muscle integrity (Figures S9 and S10). Control larvae and those exposed to malathion alone showed well-organized muscle fibers, whereas trichlorfon exposure markedly reduced birefringence in a concentration-dependent manner (up to 48% at T5, 30 mg/L), indicating disrupted muscle structure. Guanitoxin exposure caused moderate reductions at specific concentrations. Increased somite angles were observed at intermediate concentrations for trichlorfon and guanitoxin.

Combination treatments revealed enhanced effects (Figure 4). Malathion + trichlorfon mixtures showed substantial reductions in muscle integrity (∼35–47%) and showed synergistic effects in almost all combinations for somite angle (Table S11), whereas trichlorfon + guanitoxin mixtures generally exhibited antagonistic effects on muscle integrity and synergistic effects on somite angle (Table S9). Tertiary mixtures caused modest reductions (∼14%) in birefringence and increased somite angles. Notably, trichlorfon potentiated the effects of malathion, whereas malathion alone had minimal impact on muscle structure.

**Figure 4:**
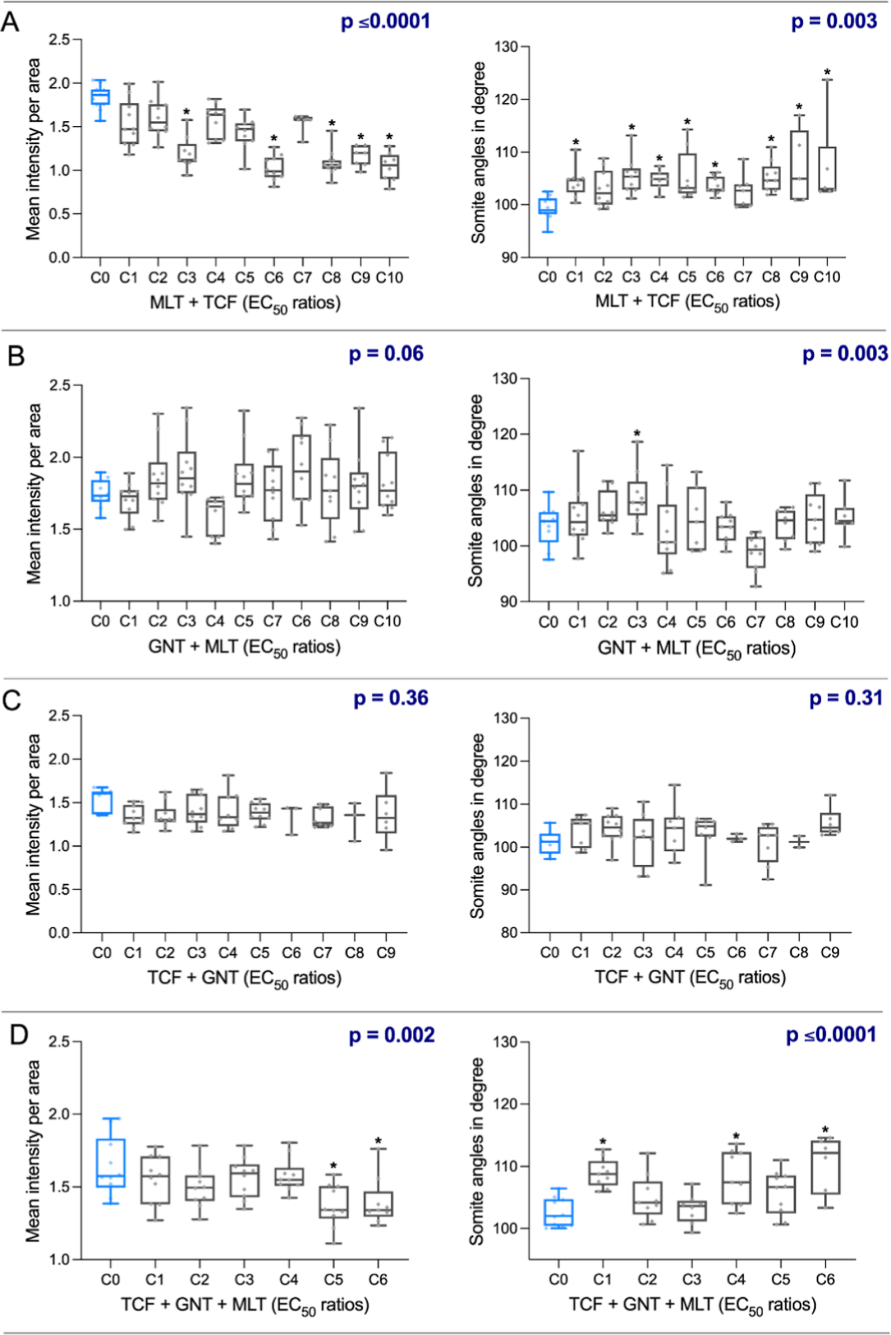
Average signal intensity per area (left panels) and somite angle in degrees (right panels) in zebrafish larvae exposed to different combinations of malathion + trichlorfon (MLT + TCF) (A), guanitoxin + malathion (GNT + MLT) (B), trichlorfon + guanitoxin (TCF + GNT) (C), and trichlorfon + guanitoxin + malathion (TCF + GNT + MLT) (D). Signal intensity and somite angles were assessed using the FIJI angle tool. Between 10 and 15 larvae were imaged and analyzed per treatment. Data are presented as boxplots, showing the median, interquartile range, and minimum/maximum values (whiskers). Asterisks (*) indicate statistically significant differences compared to the control group at each experimental time point, based on one-way ANOVA followed by Dunnett’s post hoc test (*p* ≤ 0.05). The *p*-value for each mixture is also presented. C1 to C10 or C1 to C6 represent the different chemical mixtures (ratios based on the EC_50_, expressed in mg/L; see Tables 1 and 2).

Potential mechanisms include AChE inhibition during early development, disrupting muscle formation,^7,47^ and secondary muscle fiber degeneration from neuromuscular hyperstimulation, activating calcium-dependent proteases that degrade Z-disk proteins.^48^ Increased somite angles may reflect impaired adaxial or muscle pioneer cell development via disrupted Sonic hedgehog (Shh) signaling.^49^

Birefringence provides a reliable assessment of muscle structure. However, it remains unclear whether muscle damage is a primary or secondary effect of neurotoxicity. Although muscle damage can contribute to locomotor deficits, it is often difficult to distinguish primary (muscular) from secondary (neurotoxic) effects. All combinations except MLT + TCF caused only minor alterations in muscle integrity. Notably, GNT + TCF induced pronounced locomotor impairment without detectable muscle changes by birefringence, indicating that locomotor deficits can occur overt muscle damage becomes apparent. Impaired muscle integrity likely contributes to the locomotor deficits observed across treatments; however, behavioral alterations may also result from neurodevelopmental or other physiological disturbances.

### 3.6 Transcriptomics Assessment

Transcriptomic analysis revealed that significant gene deregulation occurred exclusively in the mixture exposures in the selected concentrations and under the significance and fold change criteria chosen, i.e. a Benjamini–Hochberg (BH), adjusted *p*-value ≤0.05 and log2FoldChange ±1 (Appendix 2A). Although differentially expressed genes (DEGs) were detected for individual exposures to guanitoxin, trichlorfon, and malathion when using nonadjusted p-values, interpretation was based on Benjamini–Hochberg-adjusted values to minimize false-positive findings; DEG lists based on nonadjusted *p*-values are therefore provided in Appendix 2B, with the corresponding pathway analyses shown in Figures S11–S17. Consequently, the lack of significant DEGs in single-compound exposures likely reflects the conservative statistical thresholds applied, including multiple-testing correction, and the relatively subtle transcriptional responses induced at the tested concentrations.

Principal component analysis (PCA) revealed an overlap between treatments and control conditions (PC1 explaining 81% of the variance) (Figure S18). This overlap was primarily driven by a high dispersion among control samples, which limited the ability of PCA to clearly separate exposure groups. Consequently, PCA results should be interpreted with caution and are presented here as an exploratory analysis rather than as evidence of treatment-specific clustering. Importantly, the primary transcriptomic conclusions of this study are based on differential expression analysis (DEA) and subsequent pathway enrichment analyses, which provide statistically robust identification of treatment-related molecular responses. Despite the variability observed in the PCA, differential expression analysis identified statistically significant transcriptional responses in mixture exposures, indicating that biologically relevant effects were detectable even in the presence of high background variability. Specifically, differential expression analysis revealed that the trichlorfon + guanitoxin mixture affected the highest number of genes (1348 genes), followed by guanitoxin + malathion (513), malathion + trichlorfon (192), and the tertiary mixture trichlorfon + guanitoxin + malathion (83). These results are illustrated as volcano plots in Figure 5, and the full DEA results, including adjusted and raw p-values, are presented in Appendix 2C,D.

**Figure 5:**
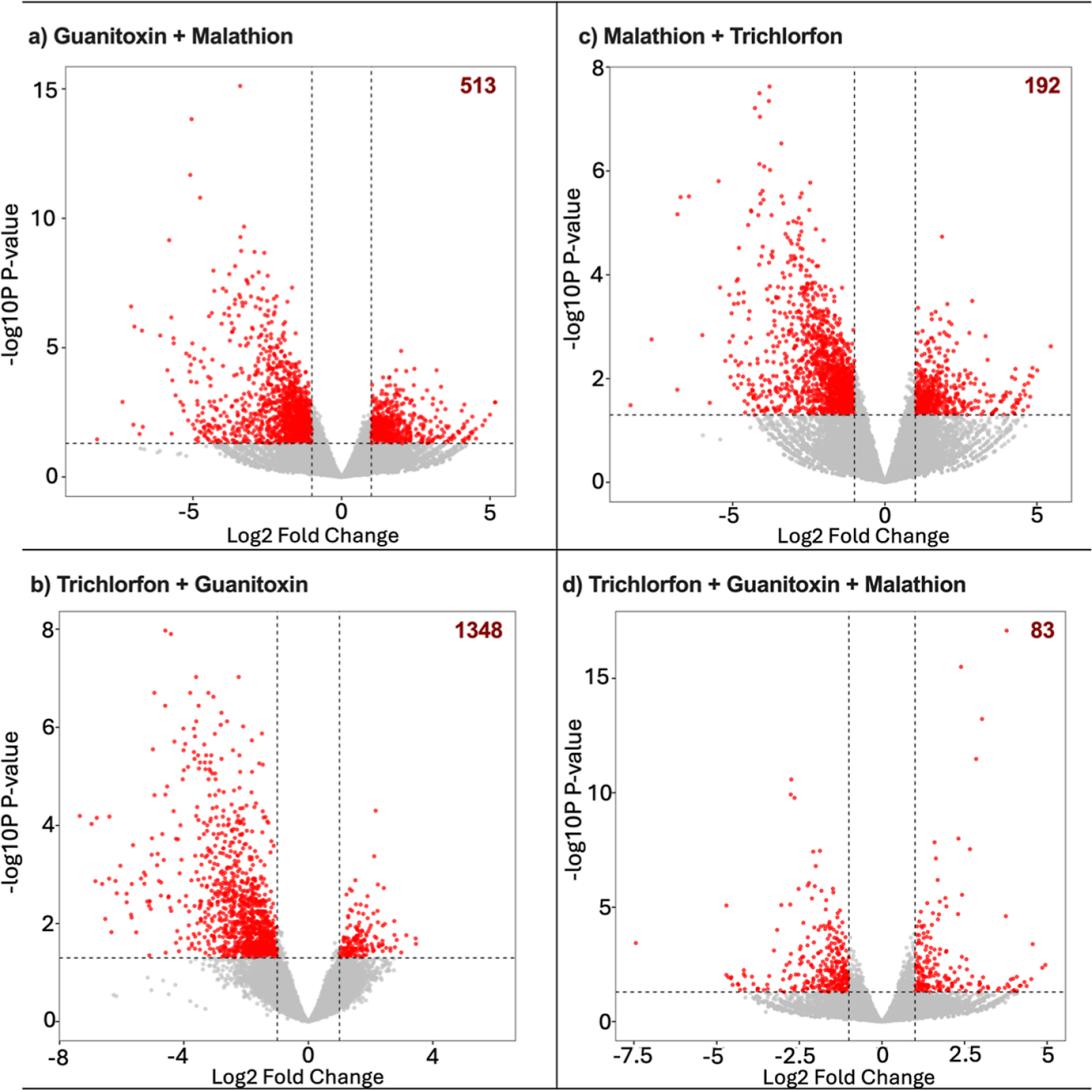
Volcano plot of differentially expressed genes. The *X*-axis represents log_2_ fold change (magnitude of expression changes), while the *Y*-axis shows—log_10_(adj *p*-value) (statistical significance). Genes further from the center and higher on the plot indicate stronger and more significant transcriptional responses. The burgundy numbers in each graph represent the total number of up- and down-regulated genes.

Gene ontology (GO) enrichment analysis of the top 25 biological processes (Figure 6A) revealed a higher number of up- and downregulated pathways, particularly in the guanitoxin-containing mixtures. Figure 6B presents a schematic highlighting the GO enrichment across one or more mixtures. The main affected processes include energy metabolism (including ATP and mitochondrial processes), muscle and heart function, visual perception, lipid metabolism, neuronal transmission, and detoxification pathways. Corresponding apical effects observed in the multibiomarker approach are indicated as bullet points and include reduced locomotor activity, increased pericardial area with decreased heart rate, reduced eye size, and compromised muscle integrity.

**Figure 6:**
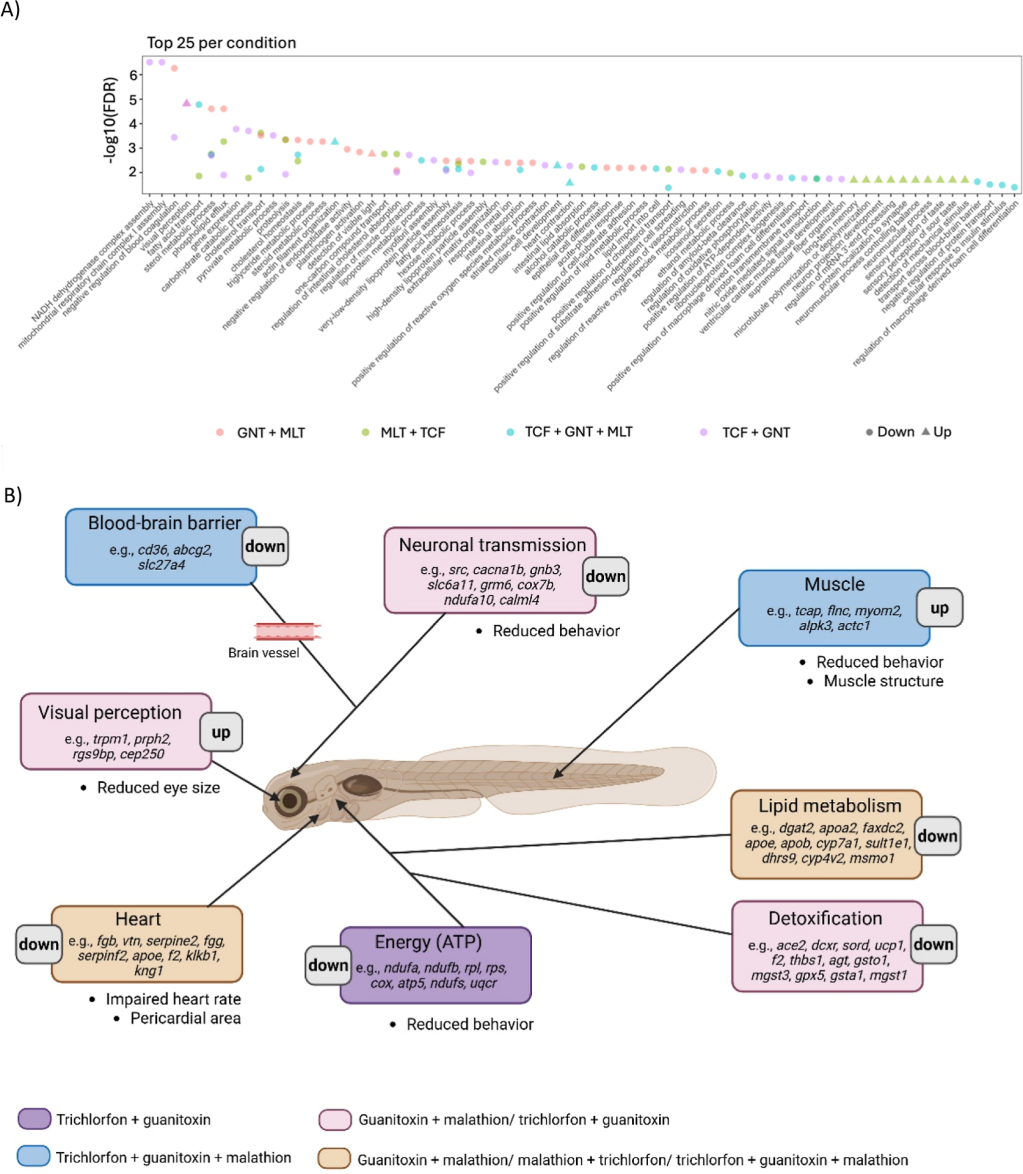
(A) Top 25 enriched gene ontology (GO) biological processes across all mixtures. Colors indicate the mixtures: lilac – trichlorfon + guanitoxin (TCF + GNT); burgundy – guanitoxin + malathion (GNT + MLT); green – malathion + trichlorfon (MLT + TCF); blue – trichlorfon + guanitoxin + malathion (TCF + GNT + MLT). Triangles indicate upregulated genes, whereas circles indicate downregulated genes. The −log_10_(FDR) represents pathway significance, with higher values indicating more statistically significant enrichment. Pathways are displayed along the horizontal dimension. (B) The schematic highlights the GO enrichment for one or more mixtures, with each mixture, or combination of mixtures, represented by a specific box color, as shown in the figure legend. Examples of differentially expressed genes are indicated within the boxes, and the corresponding apical effects are listed as bullet points.

#### 3.6.1 Energy Metabolism and Other Metabolic Processes

Trichlorfon + guanitoxin strongly downregulated genes involved in mitochondrial function and ATP production, including complexes I–IV of the respiratory chain and ATP synthase, indicating impaired oxidative phosphorylation and energy metabolism, as shown in Figure 6A (lilac circles). Different genes were affected, including *ndufa*, *ndufb*, *rpl*, *rps*, *cox*, *atp5*, *ndufs*, *uqcr*, among others. These genes are part of pathways related to processes such as cellular energy generation (ATP) through mitochondrial respiration and oxidative phosphorylation, as well as the synthesis of proteins and other macromolecules required for growth, maintenance, and metabolic regulation. Notably, all the affected genes were downregulated, highlighting the significant impact of trichlorfon + guanitoxin on fundamental bioenergetic and biosynthetic processes in the cell, reflecting broad mitochondrial dysfunction. These changes are consistent with increased oxidative stress and potential neurodegeneration.^50^

Furthermore, although they were not among the top 25 GO terms, genes involved in the regulation of reactive oxygen species (ROS), such as *ace2*, *dcxr*, *sord*, *ucp1*, *f2*, *thbs1*, and *agt* (in the guanitoxin + malathion combination), as well as genes associated with antioxidant pathways in both guanitoxin combinations were consistently downregulated (Appendix 2A). These included genes related to glutathione metabolism (e.g., *gsto1/2*, *mgst3*, *gpx5*, *gsta1*, *mgst1/3*) and metabolism of xenobiotics by cytochrome P450 (e.g., *ugt1a1*, *ugt1a5*, *hsd11b1l*, *ugt2a2*), suggesting impaired redox homeostasis, detoxification, and cellular response to stress. Moreover, the suppression of key enzymes in the citrate cycle (e.g., *pc*, *idh1*, *suclg2*, *sdhc*, *pdhb*, *pck2*) and amino acid metabolism points to potential energy deficits and disturbances in cellular metabolism. Notably, organophosphate biotransformation via cytochrome P450 can itself generate ROS, further challenging antioxidant defenses.^51^

Downregulation of PPAR (peroxisome proliferator-activated receptor) signaling were also observed for all mixtures (e.g., *fabp1*, *fabp2*, *apoa2*, *apoa1*, *cd36*, *cyp8b1*) (Appendix 2A), suggesting disruption of maintenance of metabolic homeostasis.^52^ Besides, genes involved in carbohydrate catabolism and absorption (e.g., *neu3*, *amy2a*, *pklr*, *aldob*, *gck*) were also suppressed for trichlorfon + guanitoxin and malathion + trichlorfon (Figure 6A; lilac and green circles). Together, these molecular changes indicate an energy-deficient state, disturbed lipid–glucose balance, and impaired metabolic flexibility, consistent with previous findings in other xenobiotic-exposed models,^53^ which may be factors contributing to the reduced locomotion observed.

#### 3.6.2 Lipid Metabolism and Homeostasis

Our data indicate that malathion-containing mixtures significantly disrupt lipid metabolism and homeostasis, among the top 25 enriched biological processes (Figure 6A; burgundy, green, and blue circles). The pathway affected were phospholipid efflux (e.g., *apoc1*, *apoa1*, *apoe*), sterol metabolic process (e.g., *cyp7a1*, *cyp8b1*, *hmgcr*), triglyceride biosynthesis and metabolism (e.g., *dgat1*, *dgat2*, *gck*), fatty acid homeostasis and transport (e.g., *dgat2*, *apoe*, *fabp1*, *fabp2*), and cholesterol transport, efflux, and homeostasis (e.g., *dgat2*, *mttp*, *apob*, *cyp7a1*, *npc1*, *apoe*). These alterations may compromise cellular energy balance, membrane integrity, and systemic metabolic function, consistent with previous reports of organophosphate-induced lipid dysregulation in fish and rats.^54,55^

Overall, these molecules are considered important biomarkers for monitoring alterations in lipid metabolism. For example, reduced levels of total cholesterol and triglycerides are strongly associated with liver function and may indicate an imbalance in lipid homeostasis.^56^ Likewise, the expression of genes involved in fatty acid and cholesterol synthesis was markedly reduced in larval zebrafish following exposure to the insecticide chlorpyrifos.^57^ In addition, Zhang et al.^56^ reported changes in metabolites linked to lipid metabolism pathways in adult zebrafish exposed to the pesticide propamocarb.

Moreover, a decrease in phospholipid efflux may lead to metabolic imbalances, as these molecules serve as essential precursors for a variety of biologically active compounds that regulate metabolism and physiological processes.^58^ Previous studies have shown dysregulation of lysophosphatidylcholines and phosphatidylcholines (classes of phospholipids) in juvenile *Oreochromis niloticus* (Nile tilapia) exposed to combinations of guanitoxin, malathion, and trichlorfon,^22^ underscoring the effects of these chemicals on lipid metabolism.

Together, the results herein indicate that malathion-containing mixtures induce a coordinated suppression of multiple lipid metabolic pathways, potentially compromising energy balance, membrane integrity, and systemic lipid homeostasis in developing larvae.

#### 3.6.3 Neuronal and Synaptic Impacts

The two guanitoxin-containing combinations significantly modulated neuronal function, connectivity, and plasticity, as shown in Appendix 2A. For example, genes associated with chemical synaptic transmission, including GABAergic (e.g., *src*, *cacna1b*, *gnb3*, *slc6a11*, *cacna1f*) and glutamatergic (e.g., *grm6*, *grm8*, *gnb3*, *slc17a7*, *plcb1*, *kcnj3*) neurotransmission were upregulated. The upregulation may act as a compensatory response to impaired neurotransmission, which itself may further contribute to the reduced locomotor behavior observed. Furthermore, trichlorfon + guanitoxin combination broadly suppressed genes essential for pathways linked to neurodegeneration, such as Huntington’s, Alzheimer’s, and Parkinson’s diseases (e.g., *cox7b*, *ndufa10*, *calml4*, *tubb6*, *capn2*, *gpx5*, *sigmar1*, *sdhc*, *sod2*, *uqcrc1*, *ppif*, *vdac2*, *uba52*, *atp5mc1*, *slc25a4*). Downregulation of mitochondrial respiratory chain genes (e.g., *ndufa*, *cox*, *uqcrc*, *atp5*), antioxidant defenses (e.g., *sod2*, *gpx5*) and mitochondrial transport regulators (e.g., *slc25a4*, *ucp1*), could create a pro-degenerative environment characterized by high oxidative stress. These findings align with previous evidence linking pesticide exposure to human diseases and altered antioxidant enzyme activities,^59^ as well as the established role of oxidative stress in neurological disorders, including Alzheimer’s and Parkinson’s diseases, cardiovascular conditions, and others.^60^

The tertiary mixture caused downregulation of genes of transport across blood–brain barrier (BBB) (e.g., *cd36*, *abcg2*, *slc27a4*) (Appendix 2A), suggesting vulnerability of the central nervous system (CNS) to neurotoxicants.^61^ This is particularly relevant for the insecticides, which can cross the BBB under compromised conditions, potentially exacerbating neurotoxicity.^28^ Trichlorfon is known to cross the BBB^62^ and malathion has also been shown to cross the BBB *in vitro* in brain microvascular endothelial cells.^63^ Although the direct effects of guanitoxin on BBB permeability are unknown, its presence in mixtures may contribute to neurotoxicity by synergizing with the insecticides or by directly affecting barrier function, which requires further studies to be fully understood.

#### 3.6.4 Muscle and Cardiac Function

According to Figure 6A (blue triangles), among the top 25 GO terms most enriched for muscle-related genes, the tertiary mixture had the greatest effect, showing upregulation of several genes involved in actin filament and sarcomere organization, skeletal myofibril assembly, and cardiac cell development (e.g., *tcap*, *flnc*, *myom2*, *alpk3*, *actc1*). This combination, along with the other insecticide mixtures (malathion + trichlorfon), also resulted in a complete loss of muscle integrity, as evidenced by apical birefringence (Figure 4A). These findings suggest that the larvae may be attempting to regenerate, developing and rebuilding the affected muscles.

On the other hand, in the trichlorfon + guanitoxin mixture, genes associated with muscle and heart contraction (e.g., *myh6*, *myh7*, *gamt*), sarcomere organization (e.g., *csrp1*, *synpo2l*, *tpm1*, *tnnt3*, *casq1*, *myoz1/2*, *myh6*), and the actin cytoskeleton (e.g., *itgb1*, *gsn*, *pxn*, *fn1*, *iqgap1*, *fgf10*) were consistently downregulated (Appendix 2A). Although no morphological effects were detected in the birefringence analyses, these findings suggest that exposure to this mixture induced significant molecular alterations affecting muscle structure and function, further contributing to reduced locomotion. Impaired skeletal muscle structure and associated locomotor defects have previously been linked to exposure to cholinergic insecticides^40^ and to mutations in the *ache* gene^47^ in zebrafish.

Furthermore, all mixtures containing malathion, including the tertiary mixture, exhibited downregulation of fibrinolysis-related genes (e.g., *vtn*, *serpinf2*, *f2*) (Figure 6A; burgundy, green and blue circles). Likewise, pathways associated with the regulation of blood coagulation (e.g., *fgb*, *vtn*, *serpine2*, *fgg*, *serpinf2*, *apoe*, *f2*, *klkb1*, *kng1*) were also downregulated, as shown in Figure 6A (lilac and burgundy circles) for both combinations with guanitoxin. The suppression of coagulation- and fibrinolysis-related pathways suggests that these exposures may induce systemic hemostatic dysfunction. Downregulation of key genes such as *fga*, *fgb*, *fgg*, *serpinf2*, *klkb1*, and *kng1* implies impaired clot formation and dissolution, potentially disrupting blood homeostasis and increasing susceptibility to thrombotic or hemorrhagic events.^64^

Given the essential role of hemostasis in tissue repair, vascular integrity, and neurological protection through blood–brain barrier maintenance, these findings raise concerns regarding the multisystemic toxicity of these insecticide mixtures. Further studies are warranted to determine whether these transcriptomic alterations translate into functional hematological changes and to elucidate their potential clinical relevance.

#### 3.6.5 Visual Perception

Visual perception pathways were activated in both guanitoxin coexposures (Figure 6A; lilac and burgundy triangles). Genes associated with the detection of light stimuli and visual perception were upregulated in the trichlorfon + guanitoxin (i.e., *trpm1*, *prph2*, *rgs16*, *grm8*, *guca1c*, *fscn2*, *cacna1f*, *gpr179*) and guanitoxin + malathion (i.e., *rgs9bp*, *cep250*, *tulp1*, *pax6*) groups. The upregulation of these genes suggests alterations in retinal signaling and light sensitivity, potentially affecting photoreceptor and bipolar cell function. The upregulation may also reflect a compensatory mechanism elicited by impaired retinal function, potentially arising from an energy-deficient state. Retinal neurons, similar to those in the central nervous system, have exceptionally high metabolic demands and are therefore particularly vulnerable to energy depletion.^65^ This vulnerability is illustrated by mutations in ubiquitously expressed metabolic genes that produce retina-specific phenotypes in zebrafish^66,67^ or systemic diseases with ocular manifestations in humans, such as patients suffering from pyruvate dehydrogenase deficiency.^68^

Similarly, studies with other pesticides, such as carbofuran, have shown ocular toxicity primarily mediated by cholinergic mechanisms, where AChE inhibition leads to acetylcholine accumulation and overstimulation of muscarinic and nicotinic receptors, resulting in symptoms such as miosis, tearing, and impaired visual accommodation.^69^ Such evidence supports the notion that pesticide exposure can disrupt multiple components of the visual system, from molecular signaling to retinal energy metabolism and functional vision. The eyes of humans and zebrafish are comparable in both anatomy and functionality, which raises concern since these findings highlight potential risks to human health, considering that millions of people worldwide are affected by pesticide contamination.^70^

Although information on this mechanism is lacking in the literature for guanitoxin, it is possible that it acts synergistically with insecticides, as observed in its combination with trichlorfon, where morphometric eye size measurements showed evidence of a synergistic effect in reducing eye size. Thus, this interaction could further enhance ocular toxicity and potentially serve as a driver of overall toxicity.

#### 3.6.6 Potential Molecular Mechanisms Underlying Synergistic Effects

Our transcriptomic data indicate that the combined exposure leads to amplified and system-wide molecular disturbances, supporting the occurrence of synergistic toxicity beyond simple additivity. Several nonmutually exclusive mechanisms may underlie these synergistic effects.

Multiorgan energetic failure and systemic stress responses likely integrate these molecular events. The simultaneous disruption of energy metabolism, redox balance, neuronal signaling, muscle function, and hemostasis suggests that synergism arises not from a single molecular target, but from the convergence of metabolic exhaustion, oxidative damage, and impaired compensatory capacity across multiple physiological systems. This integrated mechanistic framework provides a molecular basis for the synergistic phenotypic effects observed, although establishing direct links to all phenotypic endpoints remains challenging.

Metabolic enzyme inhibition and impaired detoxification capacity likely play a central role. The consistent downregulation of genes involved in xenobiotic metabolism, particularly cytochrome P450s and glutathione-related enzymes (e.g., *gsto*, *gpx*, *mgst*, *ugt* families), suggests a reduced capacity to biotransform and eliminate the organophosphates in mixture exposures.^71^ Such impairment may increase the internal bioavailability and persistence of both guanitoxin and insecticides, thereby potentiating toxicity and oxidative stress. Oxidative stress is widely recognized as a major mechanism underlying pesticide-induced toxicity.^72^ Previous studies have reported that inhibition of AChE activity is associated with elevated ROS levels in agricultural workers exposed to organophosphate pesticides.^72^ Nevertheless, the exact molecular processes through which either acute or chronic pesticide exposure leads to oxidative stress and cellular damage remain incompletely understood. Furthermore, the strong and coordinated downregulation of genes encoding mitochondrial respiratory chain complexes, ATP synthase, and tricarboxylic acid cycle enzymes indicates profound disruption of oxidative phosphorylation and cellular energy production.

Moreover, *cyp* family genes were implicated in pathways related to cholesterol metabolism, PPAR signaling, sterol metabolism, and lipid homeostasis (e.g., *cyp7a1*, *cyp8b1*, *cyp3a7*, *cyp51a1*, *cyp4v2*), reinforcing the role of lipid metabolic disruption in the observed toxic effects. For example, *cyp51* is essential in de novo cholesterol synthesis and is ubiquitously expressed, playing a housekeeping role in lipid metabolism.^73^ In zebrafish, *cyp51* is strongly expressed in the eye, brain, and epidermal cells of the forehead and tail fin, suggesting its involvement in critical processes such as hematopoietic and vascular development.^74^

In general, the normalized expression plots of *cyp* genes presented in Figure S19 indicate a reduction in expression relative to the control group and single-exposure treatments, further supporting the idea that the organism may not only exhibit a compromised capacity for xenobiotic biotransformation and a potential amplification of chemical stress, but also disruptions in the homeostasis of endogenous metabolites.

Morphological analyses revealed increased yolk sac area, edema, deformation, and cardiac abnormalities. These phenotypes may be indirectly associated with mitochondrial dysfunction and altered metabolic homeostasis, potentially involving hepatic impairment, which plays a central role in energy metabolism and lipid mobilization during early development. Because lipid mobilization from the yolk sac and hepatic metabolic activity are tightly coupled to mitochondrial energy production, disruptions in cholesterol and lipid metabolism, together with impaired cellular respiration, may compromise energy allocation and tissue development. Such systemic metabolic disturbances may ultimately manifest as the cardiovascular and morphological defects observed.

Additional, convergent overstimulation of cholinergic and neuronal signaling pathways may contribute to synergistic neurotoxicity. Although all compounds share cholinergic mechanisms, combined exposures may intensify acetylcholine accumulation and downstream receptor activation, overwhelming compensatory responses. The observed upregulation of genes associated with GABAergic and glutamatergic transmission may reflect an attempt to restore synaptic balance under conditions of excessive excitation, but such compensatory plasticity may itself become maladaptive, contributing to altered locomotion, neurodegenerative signaling, and sensory dysfunction.

Genes related to *ache* expression were not identified as significantly different in any comparison after correction for multiple testing. However, qualitative assessment suggests an increasing trend in *ache* expression in mixture exposures (Figure S20), which was not statistically significant and was not validated by targeted approaches such as qPCR. Importantly, AChE enzymatic activity was not directly measured in the present study; therefore, mechanistic interpretations are inferred from the concordance between phenotypic and transcriptomic responses rather than direct biochemical assessment. While phenotypic effects in individual exposures are consistent with direct AChE inhibition, the stronger responses observed in mixtures may reflect enhanced enzyme inhibition accompanied by compensatory feedback upregulation of *ache*, potentially contributing to the synergistic phenotypic effects observed. In a study with Nile tilapia exposed to guanitoxin, AChE activity in muscle tissue reached 77% inhibition, whereas in the brain it increased by 46.6%.^22^ These findings suggest that cholinergic neurotransmission may be either enhanced or suppressed depending on the tissue, likely reflecting tissue-specific differences in acetylcholine turnover, particularly in the brain and adenohypophysis.^75^ Nevertheless, further studies are required to fully elucidate the molecular mechanisms underlying this apparent activation.

### 3.7 Mixture Effects and Environmental Implications

This study provides robust evidence that both natural (guanitoxin) and synthetic (malathion and trichlorfon) organophosphates induce significant developmental toxicity in zebrafish larvae. Although additional independent replicates for the mixtures could further strengthen the robustness of the findings, the observed effects were consistent, biologically meaningful, and of high magnitude, thereby supporting the reliability of the conclusions.

Notably, guanitoxin exhibited toxicity levels comparable to synthetic insecticides, underscoring its ecological relevance during cyanobacterial bloom events. Some mixtures produced synergistic effects, amplifying harm beyond individual impacts, while others showed additive responses. Binary mixtures, particularly trichlorfon + guanitoxin, induced the most severe and widespread adverse effects, with equitoxic and high-dose combinations resulting in nearly 100% incidence of lethal or sublethal outcomes (Figure S21).

In Tables S8–S10, it is possible to see all interactions per biological parameter for each mixture (e.g., locomotion, morphology, muscle integrity). What we observe is a complex interaction between the compounds, with different types of effects occurring in the same mixture at different ratios. These differences in interaction types across the same biological parameters highlight the complexity of chemical mixture effects. A single parameter may exhibit synergistic responses at one mixture ratio and antagonistic responses at another, indicating that the relative proportions of trichlorfon, malathion, and guanitoxin critically influence the overall effect. These variations likely reflect differences in parameter-specific sensitivity, as well as distinct physiological or biochemical mechanisms triggered by each compound. For instance, synergism may result from complementary actions on the same pathway, whereas antagonism may arise when one compound counteracts or attenuates the effect of the other. This complexity is further amplified by the highly dynamic regulation of cholinergic signaling at enzymatic, transcriptional, and post-translational levels, where small changes in acetylcholine availability can shift outcomes from paralysis to hyperactivity or muscle damage, such that different combinations and concentrations of AChE inhibitors give rise to distinct interaction types. Overall, these findings emphasize that it is difficult to predict the effects of a mixture by simply summing the individual effects.

Guanitoxin, despite being a highly potent neurotoxin with a well-characterized pharmacology, remains largely overlooked in environmental monitoring. Its limited detection is due to chemical instability and incompatibility with conventional analytical methods. However, the identification of the guanitoxin biosynthetic gene cluster (BGC) has enabled detection of environmental hotspots in rural and urban areas,^13^ highlighting the need to include guanitoxin in cyanobacterial bloom monitoring programs. Organophosphate insecticides, such as malathion and trichlorfon, represent ∼45% of the global pesticide market^76^ and are frequently detected in soils, water bodies, and industrial zones. In Brazil, one of the largest pesticide users globally, organophosphate contamination has been reported even in remote areas such as the Amazon.^77^ These compounds are a serious public health concern, contributing to millions of poisonings and hundreds of thousands of deaths annually.^70^

Overall, environmentally relevant mixtures of cyanotoxins and organophosphate insecticides pose significant risks to early vertebrate development, particularly during organogenesis when detoxification capacity is low. Our findings demonstrate that even sublethal concentrations of these mixtures can induce behavioral, muscular, and molecular alterations, indicating that current single-compound risk assessments may underestimate ecological hazards.

It should be noted that the environmental occurrence and concentrations of guanitoxin remain largely unknown, as it is not routinely monitored globally and no commercial standard is available. Nevertheless, guanitoxin can be reliably detected using LC–MS/MS methods, enabling meaningful toxicological studies and risk assessment. These findings underscore the importance of incorporating mixture toxicity into environmental monitoring and regulatory frameworks, as combined exposures to cyanotoxins and organophosphate insecticides can produce significant behavioral, muscular, and molecular effects even at sublethal concentrations. Considering such interactions is critical for accurately assessing ecological risks, protecting aquatic organisms, and informing management strategies, particularly in freshwater systems affected by agricultural runoff and cyanobacterial blooms, where these mixtures are likely to occur and may impact community structure and ecosystem health.

## Supplementary Material





## Notes

Appendix 1: Analytical chemistry methods for compound quantification; transcriptomic methods and analyses; supplementary figures (Figures S1–S21); supplementary tables (Tables S1–S11) (PDF)
Appendix 2: Excel spreadsheet containing transcriptomic data sets (XLSX)
