# Supplementary material for: Environmental Mixture Toxicity of Guanitoxin and Organophosphates in Zebrafish: from Developmental and Neurobehavioral Phenotypes to Transcriptomic Responses
Source: Environ Sci Technol. 2026 Mar 11;60(12):9076–92. doi: 10.1021/acs.est.5c16673 (PMC13045019; doi:10.1021/acs.est.5c16673)
Supplement: Supplementary file 1 [file es5c16673_si_001.pdf]

Environmental mixture toxicity of guanitoxin and organophosphates in zebrafish: From developmental and neurobehavioral phenotypes to transcriptomic responses

Larissa Souza Passos[a], Melissa von Wyl[b], Elisabeth M.-L. Janssen[c], David Lopez Rodriguez[b,d,e], Ernani Pinto[a*], Colette vom Berg[b*]

[a]Laboratory of Environmental Toxicology, Center for Nuclear Energy in Agriculture, University of São Paulo, Piracicaba, São Paulo, 13416-000, Brazil.

[b]Department of Environmental Toxicology, Swiss Federal Institute of Aquatic Science and Technology, Dübendorf, 8600, Switzerland.

[c]Department of Environmental Chemistry, Swiss Federal Institute of Aquatic Science and Technology, Dübendorf, 8600, Switzerland.

[d]Department of Biomedical Sciences, University of Lausanne, Lausanne, 1005, Switzerland.

[e]Institute of Earth Surface Dynamics, University of Lausanne, Lausanne, 1015, Switzerland.

Supporting Information

This Supporting Information contains 49 pages, 21 figures, and 11 tables.

Material and Methods

Chemical concentrations

The chemical concentrations used in the individual exposures were as follows:

<u>Guanitoxin-containing extracts:</u>

T1 = 7.8125 mg/L; T2 = 15.625 mg/L; T3 = 31.25 mg/L; T4 = 62.5 mg/L; T5 = 125 mg/L.

<u>Trichlorfon:</u>

T1 = 1.875 mg/L; T2 = 3.75 mg/L; T3 = 7.5 mg/L; T4 = 15 mg/L; T5 = 30 mg/L.

Malathion:

T1 = 0.125 mg/L; T2 = 0.25 mg/L; T3 = 0.5 mg/L; T4 = 1.0 mg/L; T5 = 2.0 mg/L.

Chemical analysis

Guanitoxin was analyzed using high-resolution liquid chromatography (HRLC; Dionex UltiMate 3000 RS pump, Thermo Fisher Scientific) coupled to high-resolution tandem mass spectrometry (HRMS/MS; Orbitrap Fusion Lumos). A 20 µL injection was performed using a CTC Analytics autosampler, and chromatographic separation was achieved on an Atlantis T3 column (C18, 3 × 150 mm, 3 µm, 100 Å; Waters™) equipped with a C18 guard cartridge and an inline filter (BGB®), maintained at 30°C. Elution was performed with a binary gradient of nanopure water (solvent A) and methanol (solvent B), each containing 0.1% (v/v) formic acid, ramping from 5% to 95% B over 25 min at a flow rate of 300 µL/min.

The eluate was introduced into the HRMS/MS system (Orbitrap Fusion Lumos) via an electrospray ionization (ESI) source operating in positive ionization mode with the following parameters: capillary voltage of 3.5 kV, capillary temperature of 320 °C, sheath gas at 40 arbitrary units (AU), auxiliary gas at 10 AU, and vaporizer temperature of 275 °C. Full-scan spectra were acquired over a mass range of 100-1200 *m/z*. Guanitoxin was identified based on its characteristic fragment ions, as reported by Dörr et al. [6], using the transitions *m/z* 253 > 58 $[M + H]^+$ and *m/z* 253 > 159 $[M + H]^+$. Due to the lack of commercially available analytical standards for guanitoxin, chromatographic peak areas were normalized and reported as relative concentrations (%). Due to the lack of commercially available analytical standards and the known chemical instability of guanitoxin, chromatographic peak areas were normalized and reported as relative

concentrations (%). This relative quantification approach, based on normalized peak area comparisons between control samples and exposed groups, allowed us to assess the presence and relative stability of guanitoxin in the exposure medium between renewals.

Furthermore, both insecticides were quantified using an HP1200 HPLC-DAD system (Agilent, Germany). Chromatographic separations were performed on a Macherey-Nagel Nucleodur C18 ec analytical column (250 mm × 4.6 mm, 3 µm; Switzerland). For trichlorfon, the eluents were 100% nanopure water with 0.1% v/v formic acid (eluent A) and 100% acetonitrile with 0.1% v/v formic acid (eluent B). For malathion, the eluents were 100% nanopure water (eluent A) and 100% MeOH (eluent B). DAD detection was performed at a wavelength of 220 nm, with an injection volume of 45 µL for trichlorfon and 40 µL for malathion. The column temperature was maintained at 10°C. Calibration curves were prepared in fish embryo medium at concentrations ranging from 0.1 to 3.5 mg/L for malathion and 1.5 to 100 mg/L for trichlorfon. Calibration was performed using stock solutions of malathion and trichlorfon (1000 and 100 mg/L, respectively), followed by serial dilutions within the respective concentration ranges. Final concentrations were calculated based on the standard calibration curves: malathion (slope: 49.713; y-intercept: 4.4162; $R^2 = 0.994$) and trichlorfon (slope: 26.277; y-intercept: 214.56; $R^2 = 0.991$). The LOD and LOQ values were 0.1 mg/L and 0.25 mg/L for malathion, respectively, and 7.5 and 10 mg/L for trichlorfon, respectively.

Transcriptomics Analysis

RNA extraction

Total RNA was extracted from batches of 20 zebrafish larvae using the Qiagen RNeasy Mini Kit (Qiagen GmbH, Germany). Briefly, frozen tissue was homogenized in

a lysis buffer (RLT buffer with β-mercaptoethanol) by repeated vortexing for 30 seconds and incubation for 5 minutes. Following the procedure on purification of RNA from animal and human tissues, the RNA was then extracted using RNeasy MinElute spin columns. To reduce genomic DNA contamination, an on-column DNase I digestion was performed. RNA concentration and purity was assessed using the NanoDrop fluorometer (ThermoFisher Scientific Inc., USA). To ensure that the extracted RNA was intact, the samples were further tested on the 2200 TapeStation system (Agilent Technologies, USA).

RNA library construction

The Lexogen QuantSeq 3' mRNA-Seq V2 (FWD) kit with 12-nucleotide Unique Dual Indices (UDIs) was employed for the preparation of Illumina-compatible libraries from polyadenylated RNA. This protocol generates a single fragment per transcript, enabling accurate gene expression quantification with sequences enriched near the 3' end of transcripts. Library construction was initiated by first-strand cDNA synthesis through reverse transcription using an oligo(dT) primer containing a 5' adapter sequence. Following this step, the RNA template was enzymatically degraded to facilitate efficient synthesis of the second cDNA strand. Second-strand synthesis was primed by a random primer harboring a 5' sequence, resulting in double-stranded DNA (dsDNA). The resulting libraries were purified using magnetic beads to remove residual reaction components. Subsequently, libraries were amplified via PCR to incorporate full-length adapter sequences and 12-nt UDIs, enabling multiplexing. For each sample, 10 μl of the appropriate UDI primer pair was added, with one unique index pair per sample, and PCR was performed for 12 to 26 cycles. Final libraries were purified to eliminate remaining

PCR reagents and prepared for quality control assessment, pooling, and cluster generation for high-throughput sequencing.

RNA sequencing and data processing

The mRNA library, obtained from pooled samples, underwent sequencing at the Functional Genomics Center Zurich (FGCZ), Switzerland. Before proceeding with high-throughput sequencing, the quality of the mRNA was verified using the Illumina iSeq platform. This preliminary, low-yield sequencing step served to evaluate library quality, determine the optimal loading concentration, and assess index distribution. Samples identified as underrepresented during the iSeq analysis were adjusted accordingly by spiking them into the final mRNA pool. The concentration and integrity of these adjusted samples were subsequently confirmed using the ScreenTape assay (Agilent). The final sequencing was conducted on an Illumina NovaSeq X platform with a 300-cycle 10B flow cell, generating an average of 20 million paired-end reads (2×150 bp) per sample.

The resulting raw data were processed by the Genetic Diversity Centre, ETH Zürich (GDC). For transcript quantification, pseudo-alignment was carried out using Kallisto (v0.50.1), based on the Ensembl reference transcriptome for *D. rerio* (GRCz11/GCA_000002035.4).

Data analysis

Following transcript quantification, data analysis was performed using Deseq2 (v1.48.1) in R (v4.5.0). First, low-count genes (<10) were filtered prior to analysis and counts were normalized using DESeq2 size factor estimation, correcting for library size and sequencing depth. Then, transcriptomic variability between samples was assessed using a principal component analysis (PCA), performed on variance-stabilized

transformed (VST) counts obtained using the vst() function from the DESeq2. Sample clustering and replicate consistency were further evaluated by computing pairwise Euclidean distances between samples using VST counts, which were visualized as a sample-to-sample distance matrix. Furthermore, potential batch effects were evaluated based on sample metadata and PCA visualization. All samples were processed using the same experimental pipeline and sequencing platform. PCA of variance-stabilized counts did not reveal clustering associated with technical variables.

Next, the DESeq2 results() function was used to compute differential expression analysis (DEA) using Wald tests between exposure conditions and their respective control conditions. Genes were considered significantly differentially expressed if they exhibited an absolute log fold change greater/lower than 1 and a Benjamini-Hochberg (BH) adjusted p-value $\leq$ 0.05. Volcano plots were generated using ggplot2. Significant upregulated and downregulated genes for each condition were used to perform a biological function enrichment analysis using enrichR (v3.2) (library: GO Biological Process 2023 database). Gene names and human gene name homologs were used for this analysis, obtained from BioMart (v2.60) and org.Dr.eg.db. Venn diagrams were generated using the VennDiagram package (v1.7.3).

Supplementary Tables and Figures

Table S1. Chemical compounds used in the study, guanitoxin, trichlorfon, and malathion, with their basic structures, molecular weights (g/mol), chemical formulas, water solubility (mg/L), log Kow or pKa values, and chemical structures.

Compound	Basic structure	Molecular weight (g/mol)	Chemical formula	Solubility in water (mg/L)	log K_{ow}/pka	Chemical structure
Guanitoxin (GNT)	Organophosphate	252.21	$C_7H_{17}N_4O_4P$	Highly soluble	8.4[c]/ -9	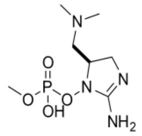
Trichlorfon (TCF)	Organophosphate	257.43	$C_4H_8Cl_3O_4P$	1.20×10^5	0.51/ 10.12	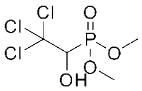
Malathion (MLT)	Organophosphate	330.36	$C_{10}H_{19}O_6PS_2$	143	2.36/ -6.8	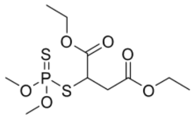

Table S2. Raw data normalized to the control group from individual experiments, expressed as percentages (%), used for the heatmap (Figure 8). A negative sign indicates down-regulation (−), and a positive sign indicates up-regulation (+). T1 to T5 represent the different concentrations of each chemical, ranging from the lowest to the highest concentration, as described in Table 1.

Guanitoxin					
Biological parameters	T1	T2	T3	T4	T5
Body length	1,47	-0,11	-1,23	-1,42	-3,60
Eye size	0,27	-4,89	-12,70	-16,70	-20,52
Swim bladder area	-25,63	-38,07	-41,44	-38,15	-53,80
Yolk area	7,99	5,33	2,34	3,69	8,26
Pericardial area	4,67	2,96	6,49	9,18	60,79
Heartbeat	-0,03	-1,16	-5,71	2,25	-2,30
Locomotion spontaneous	-24,78	-10,41	-14,28	-7,06	3,56
Locomotion light	-11,22	-7,79	-7,99	2,14	3,89
Locomotion dark	-16,48	-26,75	-31,54	-39,09	-51,00
Muscle integrity	-2,72	-16,89	-7,06	-13,90	-17,32
Somite angle	-3,40	-1,99	-5,85	-1,46	0,63
Trichlorfon					
Biological parameters	T1	T2	T3	T4	T5
Body length	-1,22	-3,12	-5,08	-14,08	-23,18
Eye size	-9,26	-11,99	-18,78	-32,71	-46,20
Swim bladder area	-38,47	-40,44	-47,52	-54,32	-59,37
Yolk area	0,66	6,91	10,25	12,75	41,27
Pericardial area	2,23	4,12	5,64	43,82	357,40
Heartbeat	-3,41	3,98	3,40	4,56	-50,00
Locomotion spontaneous	-16,69	-8,23	-10,19	4,83	15,48
Locomotion light	-11,36	-10,55	-3,81	-1,90	-31,49
Locomotion dark	-33,86	-51,96	-72,84	-79,98	-98,15
Muscle integrity	-7,36	-11,77	-29,25	-29,16	-44,75
Somite angle	-7,07	-5,52	-6,70	-5,16	-5,87
Malathion					
Biological parameters	T1	T2	T3	T4	T5
Body length	-1,28	-0,44	-0,36	-2,44	-7,45
Eye size	-3,11	-3,51	-4,93	-13,74	-20,90
Swim bladder area	-55,24	-55,56	-61,68	-61,36	-72,40
Yolk area	-2,75	1,51	0,33	1,10	4,46
Pericardial area	9,53	15,58	11,51	19,66	41,85
Heartbeat	-3,13	-4,17	4,16	-4,17	-6,26
Locomotion spontaneous	-22,27	-15,45	-19,90	-24,08	-26,88
Locomotion light	-15,40	-6,61	-2,74	9,48	18,27
Locomotion dark	10,73	7,44	6,48	-27,92	-72,93
Muscle integrity	-10,51	-15,67	-17,85	-10,45	-9,02
Somite angle	3,33	-6,14	2,75	0,13	1,82

Table S3. Raw data normalized to the control group from binary and tertiary combination experiments, expressed as percentages, used for the heatmap (Figure 1).

MLT + TCF										
Biological parameters	C1	C2	C3	C4	C5	C6	C7	C8	C9	C10
Body length	-0,24	-0,82	-5,35	-2,63	-11,69	-4,17	-3,32	-7,60	-15,55	-18,32
Eye size	-8,01	-11,32	-21,19	-11,94	-31,02	-12,92	-14,08	-21,81	-32,83	-42,95
Swim bladder area	-22,19	206,30	-45,57	-33,67	-40,09	-32,86	-37,84	-38,34	-46,38	-55,17
Yolk sac area	0,56	16,66	10,29	6,72	21,08	14,94	7,19	18,69	29,76	77,78
Pericardial area	4,55	18,02	14,06	18,35	48,88	11,98	18,48	13,96	158,48	435,71
Heartbeat	-3,45	3,43	1,70	10,34	0,00	3,41	-0,04	3,45	3,43	-6,91
Locomotion spontaneous	-56,24	-51,74	-70,02	-56,21	-72,18	-44,74	-46,92	-56,83	-61,76	-53,84
Locomotion light	-21,40	-12,09	-33,92	-36,39	-49,67	-20,64	-16,69	-29,70	-40,12	-30,65
Locomotion dark	-45,83	-53,63	-87,74	-75,12	-85,87	-70,40	-70,75	-84,93	-87,19	-72,16
Muscle integrity	-21,08	-16,91	-39,86	-12,14	-20,94	-46,96	-15,31	-43,03	-35,58	-43,22
Somite angle	5,75	3,27	6,46	5,98	4,28	3,98	3,76	5,67	6,07	4,07
GNT + MLT										
Biological parameters	C1	C2	C3	C4	C5	C6	C7	C8	C9	C10
Body length	-2,21	0,64	-3,47	0,89	-7,14	-1,68	-1,41	-2,22	-8,55	-6,53
Eye size	-2,40	-2,11	-15,73	-2,85	-31,92	-11,24	-6,54	-10,86	-26,41	-26,62
Swim bladder area	-45,81	-56,31	-60,61	-57,95	-68,64	-57,18	-58,75	-58,21	-66,74	-73,96
Yolk area	-2,36	-0,03	8,96	-2,67	14,70	-0,20	-2,78	5,64	16,81	9,06
Pericardial area	13,87	26,42	26,75	14,73	80,32	53,90	33,03	54,36	141,02	121,53
Heartbeat	-7,95	1,54	-11,14	-1,60	-20,63	-1,58	-11,12	-4,75	-11,12	-12,70
Locomotion spontaneous	37,84	19,19	130,65	-21,89	88,56	-10,08	82,04	-21,21	-40,35	29,72
Locomotion light	-13,85	11,03	36,15	11,64	82,41	-8,30	45,39	9,70	22,70	7,19
Locomotion dark	-18,33	-34,97	-36,29	-0,11	-41,64	-35,58	-18,17	-27,95	-79,63	-49,69
Muscle integrity	-0,20	5,02	7,01	-4,28	4,76	9,79	2,24	2,01	3,97	2,01
Somite angle	-0,16	1,04	3,24	-3,60	-0,07	-0,94	-4,86	0,16	0,28	0,04
TCF + GNT										

Biological parameters	C1	C2	C3	C4	C5	C6	C7	C8	C9	C10
Body lenght	-81,59	-80,63	-80,78	-82,14	-80,38	-83,89	-81,24	-	-83,66	-83,74
Eye size	-10,85	1,96	-3,01	-21,40	-3,48	-46,59	-7,46	-	-41,93	-43,16
Swim bladder area	-24,94	-27,03	-12,02	-43,79	-17,80	-57,34	-28,44	-	-62,10	-57,22
Yolk area	10,01	7,82	16,63	18,01	13,98	31,73	10,84	-	11,40	20,18
Pericardial area	13,02	29,08	15,57	27,76	24,19	670,62	12,16	-	112,46	212,76
Heartbeat	-3,30	0,03	0,03	0,03	0,02	-60,01	-0,03	-	-29,98	-73,33
Locomotion spontaneous	-40,59	-19,93	-25,08	-33,05	15,81	-49,12	-4,53	-	-45,71	-55,88
Locomotion light	-47,59	-47,24	-38,30	-59,71	-20,54	-58,74	-32,61	-	-51,31	25,29
Locomotion dark	-59,93	-53,45	-54,00	-77,28	-47,13	-84,11	-54,20	-	-76,10	-58,69
Muscle integrity	-17,51	-18,81	-14,73	-16,98	-13,68	-10,64	-21,01	-	-15,40	-17,48
Somite angle	4,22	3,28	1,03	3,16	3,56	0,66	1,44	-	-0,01	3,23

TCF + GNT + MLT

Biological parameters	C1	C2	C3	C4	C5	C6	C7	C8	C9	C10
Body lenght	2,13	-0,38	-3,94	2,47	-0,39	-4,62				
Eye size	-2,92	-5,32	-14,45	-3,22	-16,41	-30,06				
Swim bladder area	-16,66	-24,00	-36,27	-33,17	-40,52	-49,52				
Yolk area	-1,28	-1,77	9,38	1,26	3,28	19,99				
Pericardial area	8,91	24,83	22,89	27,68	50,74	94,40				
Heartbeat	-2,19	-13,06	4,33	-4,37	-4,35	-0,02				
Locomotion spontaneous	2,44	-6,44	-24,96	8,70	-11,06	-36,24				
Locomotion light	85,03	30,77	10,38	13,67	16,25	-13,11				
Locomotion dark	-44,17	-70,54	-82,17	-35,37	-77,72	-85,05				
Muscle integrity	-0,09	-5,14	1,10	-1,69	-14,83	-14,91				
Somite angle	6,62	2,10	1,56	5,31	4,57	9,93				

Table S4. Malathion and trichlorfon concentrations (mg/L) and guanitoxin relative concentration (%) in the water samples from the Fish Embryo Toxicity test at the beginning of the experiment (0 hours), at the middle (48 and 72 hours) and at the end (120 hours). Data are presented as mean in mg/L.

Malathion (mg/L)						
Time	0 mg/L	0.125 mg/L	0.25 mg/L	0.5 mg/L	1 mg/L	2 mg/L
0h	0	0.122	0.309	0.649	1.328	2.838
48h	0	<LQ	0.182	0.275	0.573	1.227
96h	0	<LQ	0.188	0.205	0.579	0.934
120h	0	<LQ	<LQ	0.207	0.575	1.300
Trichlorfon (mg/L)						
Time	0 mg/L	1.875 mg/L	3.75 mg/L	7.5 mg/L	15 mg/L	30 mg/L
0h	0	<LQ	2.50	6.87	11.45	27.60
48h	0	<LQ	2.70	4.33	8.86	17.05
96h	0	<LQ	1.23	4.62	11.31	20.40
120h	0	<LQ	<LQ	2.09	7.25	12.81
Guanitoxin extracts (%)						
Time	0 mg/L	7.81 mg/L	15.62 mg/L	31.25 mg/L	62.5 mg/L	125 mg/L
0h	0	63.55	58.30	67.33	54.88	65.27
48h	0	34.61	56.38	62.48	61.04	61.07
96h	0	49.53	53.90	53.94	51.31	61.20
120h	0	11.82	30.37	44.34	40.93	57.52

Table S5. Morphological damages caused in *Danio rerio* larvae after 120 hours of exposure to guanitoxin extracts, trichlorfon, and malathion individually, besides binary and tertiary combinations. C = coagulation; T = tail not detached; H = no heartbeat; LH = lack of hatching; MH = malformed head; MT = malformed tail; EY = modified eye development; A = modified axis structure; Y = yolk deformations; HE = heart edema; YE = yolk edema; M = uncontrolled movements and trembling; P = no pigmentation, and NR = no reaction after trigger.

Guanitoxin

Treatments	Exposure group (mg/L)	Total affected larvae (%)	Frequency of morphological alterations observed (%)													
			Lethal endpoints				Sub-lethal endpoints									
			C	T	H	LH	MH	MT	EY	A	Y	HE	YE	M	P	NR
T0	0	2.3	0	0	0	0	0	0	0	0	0	0	0	0	2.3	0
T1	7.81	40.7	0	0	0	0	0	0	0	3.7	0	7.4	3.7	33.3	3.7	0
T2	15.62	79.3	0	0	6.9	13.8	0	0	3.4	0	0	10.3	0	62.1	0	0
T3	31.25	60.9	0	0	4.3	2.2	10.9	4.3	2.2	8.7	15.2	32.6	21.7	47.8	0	4.3
T4	62.5	71.7	0	0	0	0	0	0	0	6.5	13.0	23.9	23.9	71.7	0	0
T5	125	100	0	0	9.5	2.4	0	2.4	0	9.5	31.0	76.2	64.3	71.4	2.4	4.8

Trichlorfon

Treatments	Exposure group (mg/L)	Total affected larvae (%)	Frequency of morphological alterations observed (%)													
			Lethal endpoints				Sub-lethal endpoints									
			C	T	H	LH	MH	MT	EY	A	Y	HE	YE	M	P	NR
T0	0	0	0	0	0	0	0	0	0	0	0	0	0	0	0	0
T1	1.875	12.8	0	0	4.3	4.3	0	0	0	0	0	8.5	2.1	4.3	0	0
T2	3.75	15.6	0	0	4.4	0	0	2.2	2.2	4.4	8.9	8.9	6.7	0	2.2	4.4
T3	7.5	22.2	0	0	2.2	0	0	2.2	0	2.2	4.4	13.3	6.7	4.4	2.2	2.2
T4	15	97.9	0	0	4.3	0	0	4.3	0	6.4	29.8	63.8	85.1	61.7	4.3	8.5
T5	30	100	0	0	29.5	0	0	0	0	27.3	90.9	95.5	95.5	15.9	4.5	54.5

Malathion

Treatments	Exposure group (mg/L)	Total affected larvae (%)	Frequency of morphological alterations observed (%)													
			Lethal endpoints				Sub-lethal endpoints									
			C	T	H	LH	MH	MT	EY	A	Y	HE	YE	M	P	NR

T0	0	4.3	0	0	0	0	0	2.2	0	2.2	0	2.2	0	4.3	0	0
T1	0.125	25.0	0	0	0	0	0	0	0	0	0	6.3	0	21.9	0	0
T2	0.25	28.1	0	0	0	0	0	0	0	0	0	9.4	0	21.9	0	0
T3	0.5	53.1	3.1	0	0	0	0	0	0	0	0	3.1	0	46.9	0	0
T4	1	66.7	0	0	0	0	0	0	0	0	8.3	10.4	4.2	60.4	2.1	0
T5	2	97.9	0	0	0	4.2	0	2.1	0	8.3	27.1	43.8	33.3	87.5	0	6.3

Table S6. Morphological damages caused in *Danio rerio* larvae after 120 hours of exposure to binary and tertiary combinations of guanitoxin, trichlorfon, and malathion. C = coagulation; T = tail not detached; H = no heartbeat; LH = lack of hatching; MH = malformed head; MT = malformed tail; EY = modified eye development; A = modified axis structure; Y = yolk deformations; HE = heart edema; YE = yolk edema; M = uncontrolled movements and trembling; P = no pigmentation, and NR = no reaction after trigger.

Guanitoxin + Malathion

Combinations	Exposure group (ratios)	Total affected larvae (%)	Frequency of morphological alterations observed (%)													
			Lethal endpoints				Sub-lethal endpoints									
			C	T	H	LH	MH	MT	EY	A	Y	HE	YE	M	P	NR
C0	0	3.4	0	0	0	0	0	0	0	0	0	3.4	0	0	0	0
C1	0.5 + 0.25	81.3	0	0	0	0	0	0	0	0	0	12.5	0	62.5	0	0
C2	0.25 + 0.5	80.0	0	0	0	0	0	0	0	0	0	0	0	81.3	6.3	0
C3	0.5 + 1	68.8	0	0	0	0	18.8	0	12.5	0	12.5	37.5	18.8	50	0	0
C4	1 + 0.5	100	12.5	0	12.5	18.8	37.5	6.25	31.25	12.5	37.5	37.5	37.5	31.3	0	6.3
C5	0.5 + 1.5	87.5	0	0	0	0	0	0	0	0	0	0	0	80	0	0
C6	1.5 + 0.5	100	0	0	0	0	0	0	0	0	0	13.3	6.7	100	0	0
C7	0.5 + 0.5	62.5	13.3	0	20	20	20	20	20	20	33.3	66.7	40	66.7	0	13.3
C8	1 + 1	100	0	0	0	0	7.1	7.1	7.1	7.1	7.1	42.9	14.3	100	0	0
C9	1.5 + 1.5	100	0	0	0	14.3	42.9	21.4	35.7	35.7	50	71.4	50	78.6	0	21.4
C10	2 + 2	100	18.8	0	18.8	18.8	31.3	37.5	6.3	37.5	50	75	75	68.8	0	12.5

Malathion + Trichlorfon

Combinations	Exposure group (ratios)	Total affected larvae (%)	Frequency of morphological alterations observed (%)													
			Lethal endpoints				Sub-lethal endpoints									
			C	T	H	LH	MH	MT	EY	A	Y	HE	YE	M	P	NR
C0	0	6.5	0	0	0	0	0	0	0	0	3.2	3.2	3.2	3.2	0	0
C1	0.5 + 0.25	43.8	0	0	6.3	6.3	6.3	6.3	0	6.3	6.3	6.3	6.3	37.5	0	0
C2	0.25 + 0.5	100	0	0	0	0	0	0	0	0	0	0	25	100	6.3	0
C3	0.5 + 1	100	0	0	0	6.3	6.3	0	0	6.3	6.3	6.3	56.3	93.8	0	6.3
C4	1 + 0.5	100	0	0	0	0	6.3	6.25	6.3	12.5	6.3	6.3	18.8	93.8	0	6.3

C5	0.5 + 1.5	100	0	0	0	0	12.5	0	12.5	18.8	43.8	56.3	100	93.8	0	6.3
C6	1.5 + 0.5	100	0	0	0	0	0	0	0	0	0	0	25	100	0	0
C7	0.5 + 0.5	93.8	12.5	0	12.5	12.5	25	25	12.5	25	25	25	25	68.8	6.3	12.5
C8	1 + 1	100	0	0	0	0	6.3	0	12.5	6.25	12.5	18.8	50	93.8	0	6.3
C9	1.5 + 1.5	100	0	0	0	0	43.8	6.25	37.5	12.5	50	68.8	81.3	75	0	18.8
C10	2 + 2	100	0	0	0	0	73.3	0	60	13.3	86.7	93.3	100	60	20	40

Trichlorfon + Guanitoxin

Combinations	Exposure group (ratios)	Total affected larvae (%)	Frequency of morphological alterations observed (%)													
			Lethal endpoints				Sub-lethal endpoints									
			C	T	H	LH	MH	MT	EY	A	Y	HE	YE	M	P	NR
C0	0	0	0	0	0	0	0	0	0	0	0	0	0	0	0	0
C1	0.5 + 0.25	68.8	0	0	0	0	0	0	0	0	0	0	12.5	56.3	0	0
C2	0.25 + 0.5	43.8	6.3	0	6.3	6.3	6.3	6.3	0	6.3	6.3	6.3	6.3	31.3	6.3	0
C3	0.5 + 1	37.5	0	0	0	0	0	0	0	0	0	0	0	37.5	0	0
C4	1 + 0.5	100	6.3	0	6.3	12.5	12.5	12.5	12.5	0	31.3	43.8	75	68.8	0	18.8
C5	0.5 + 1.5	50	0	0	0	0	0	0	0	0	0	0	6.3	43.8	0	0
C6	1.5 + 0.5	100	56.3	6.3	56.3	50	75	6.3	62.5	18.8	93.8	100	100	12.5	0	31.3
C7	0.5 + 0.5	81.3	0	0	0	0	6.3	6.3	6.3	6.3	12.5	12.5	12.5	75	0	6.3
C8	1 + 1	100	73.3	6.7	86.7	73.3	86.7	73.3	86.7	86.7	86.7	86.7	93.3	13.3	0	0
C9	1.5 + 1.5	100	50	6.3	50	62.5	75	62.5	75	68.8	75	93.8	93.8	25	0	25
C10	2 + 2	100	12.5	0	6.25	6.3	50	25	43.8	31.3	75	93.8	93.8	62.5	0	31.3

Trichlorfon + Guanitoxin + Malathion

Combinations	Exposure group (ratios)	Total affected larvae (%)	Frequency of morphological alterations observed (%)													
			Lethal endpoints				Sub-lethal endpoints									
			C	T	H	LH	MH	MT	EY	A	Y	HE	YE	M	P	NR
C0	0	4.3	0	0	0	0	0	0	0	0	0	4.3	0	0	0	0
C1	0.1 + 0.1 + 0.5	31.6	0	0	0	0	0	5.3	0	0	0	5.3	0	47.4	0	0
C2	0.1 + 0.5 + 0.1	73.7	10.5	0	10.5	10.5	5.3	5.3	0	5.3	5.3	15.8	10.5	47.4	0	0
C3	0.5 + 0.1 + 0.1	100	5	0	5	5	5	0	5	5	10	25	20	90	0	0
C4	0.1 + 0.1 + 0.1	68.4	5.3	0	5.3	5.3	0	0	0	0	0	42.1	0	52.6	0	0
C5	0.25 + 0.25 + 0.25	80	0	0	0	0	0	0	0	0	5	50	25	75	5	0
C6	0.5 + 0.5 + 0.5	100	5.3	0	5.3	5.3	42.1	5.3	31.6	10.5	52.6	73.7	68.4	94.7	0	0

Table S7. Effects of the mixtures on zebrafish larvae. Exposure ratios indicate the proportion of each compound in the mixture. Toxic Unit (ΣTU) represents the sum of the relative toxicities of the individual compounds at the given concentrations, calculated using the Concentration Addition (CA) model. Predicted effect (%) refers to the expected effect based on the additive model of the individual compound effects. Total affected larvae (%) indicates the observed percentage of larvae showing effects under mixture exposure in the FET assay. Interaction type classifies the combined response as antagonistic, additive, or synergistic based on the comparison between observed and predicted effects.

Guanitoxin + Malathion

Combinations	Exposure ratios	Toxic Unit (ΣTU)	Predicted effect (%)	Total affected larvae (%)	Interaction type
C1	0.5 + 0.25	0.75	37.5	81,3	Synergism
C2	0.25 + 0.5	0.75	37.5	80,0	Synergism
C3	0.5 + 1	1.5	75	68,8	Antagonism
C4	1 + 0.5	1.5	75	100	Synergism
C5	0.5 + 1.5	2	100	87,5	Antagonism
C6	1.5 + 0.5	2	100	100	Additivity
C7	0.5 + 0.5	1	50	62,5	Synergism
C8	1 + 1	2	100	100	Additivity
C9	1.5 + 1.5	3	100	100	Additivity
C10	2 + 2	4	100	100	Additivity

Malathion + Trichlorfon

Combinations	Exposure ratios	Toxic Unit (ΣTU)	Predicted effect (%)	Total affected larvae (%)	Interaction type
C1	0.5 + 0.25	0.75	37.5	43,8	Synergism
C2	0.25 + 0.5	0.75	37.5	100	Synergism
C3	0.5 + 1	1.5	75	100	Synergism
C4	1 + 0.5	1.5	75	100	Synergism
C5	0.5 + 1.5	2	100	100	Additivity
C6	1.5 + 0.5	2	100	100	Additivity
C7	0.5 + 0.5	1	50	93,8	Synergism
C8	1 + 1	2	100	100	Additivity
C9	1.5 + 1.5	3	100	100	Additivity
C10	2 + 2	4	100	100	Additivity

Trichlorfon + Guanitoxin

Combinations	Exposure ratios	Toxic Unit (ΣTU)	Predicted effect (%)	Total affected larvae (%)	Interaction type

C1	0.5 + 0.25	0.75	37.5	68,8	Synergism
C2	0.25 + 0.5	0.75	37.5	43,8	Synergism
C3	0.5 + 1	1.5	75	37,5	Antagonism
C4	1 + 0.5	1.5	75	100	Synergism
C5	0.5 + 1.5	2	100	50,0	Antagonism
C6	1.5 + 0.5	2	100	100	Additivity
C7	0.5 + 0.5	1	50	81,3	Synergism
C8	1 + 1	2	100	100	Additivity
C9	1.5 + 1.5	3	100	100	Additivity
C10	2 + 2	4	100	100	Additivity
Trichlorfon + Guanitoxin + Malathion					
Combinations	Exposure ratios	Toxic Unit (ΣTU)	Predicted effect (%)	Total affected larvae (%)	Interaction type
C1	0.1 + 0.1 + 0.5	0.70	35	31.6	Additivity
C2	0.1 + 0.5 + 0.1	0.70	35	73.7	Synergism
C3	0.5 + 0.1 + 0.1	0.70	35	100	Synergism
C4	0.1 + 0.1 + 0.1	0.3	15	68.4	Synergism
C5	0.25 + 0.25 + 0.25	0.75	37.5	80	Synergism
C6	0.5 + 0.5 + 0.5	1.5	75	100	Synergism

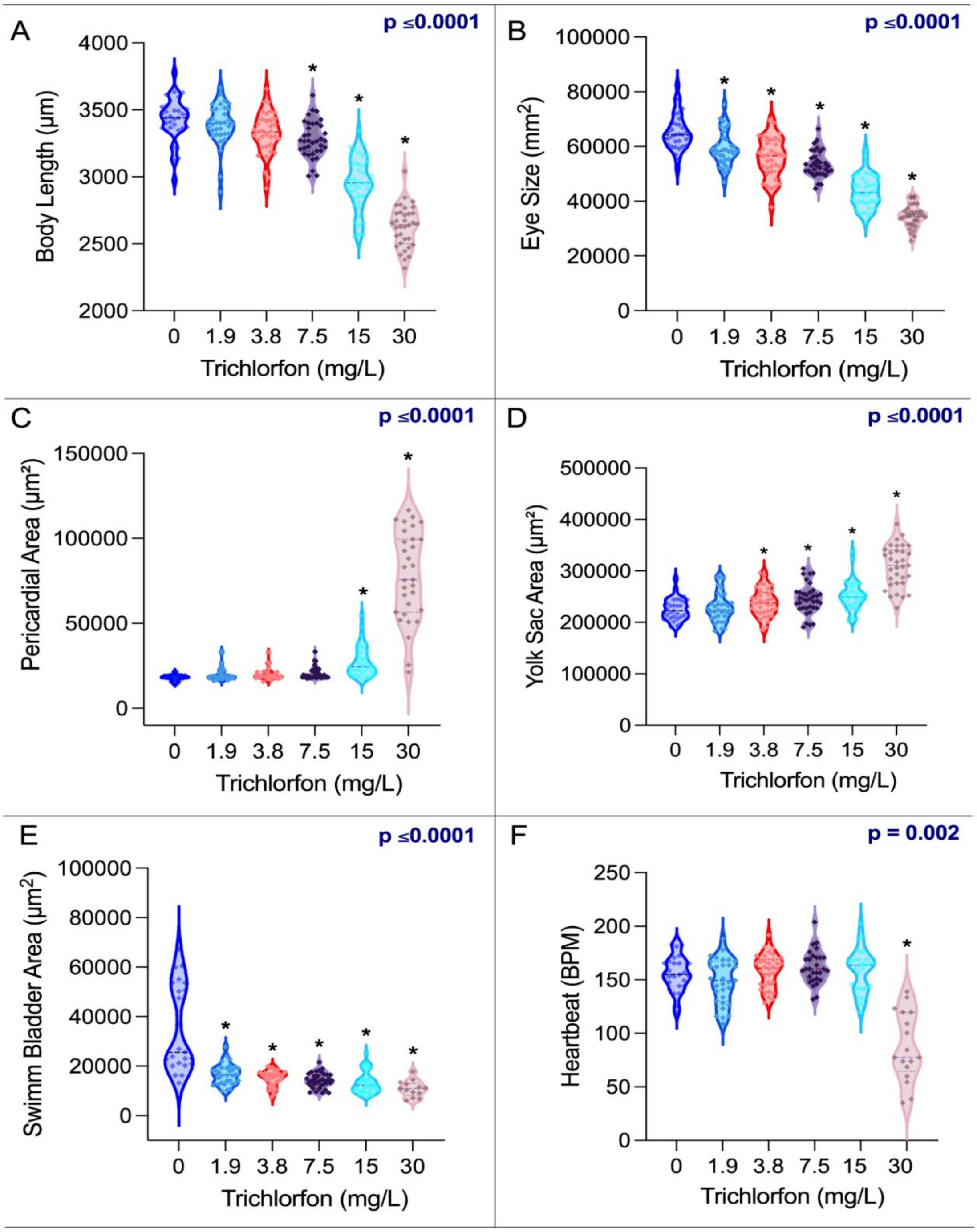

Figure S1. Body length (µm), eye size (mm^2), pericardial area (μm^2), yolk area (μm^2), swim bladder area (μm^2), and heartbeat (BPM) of *Danio rerio* larvae exposed to trichlorfon for 120 hours. Asterisks (*) indicate statistically significant differences according to ANOVA and Dunnett post hoc tests.

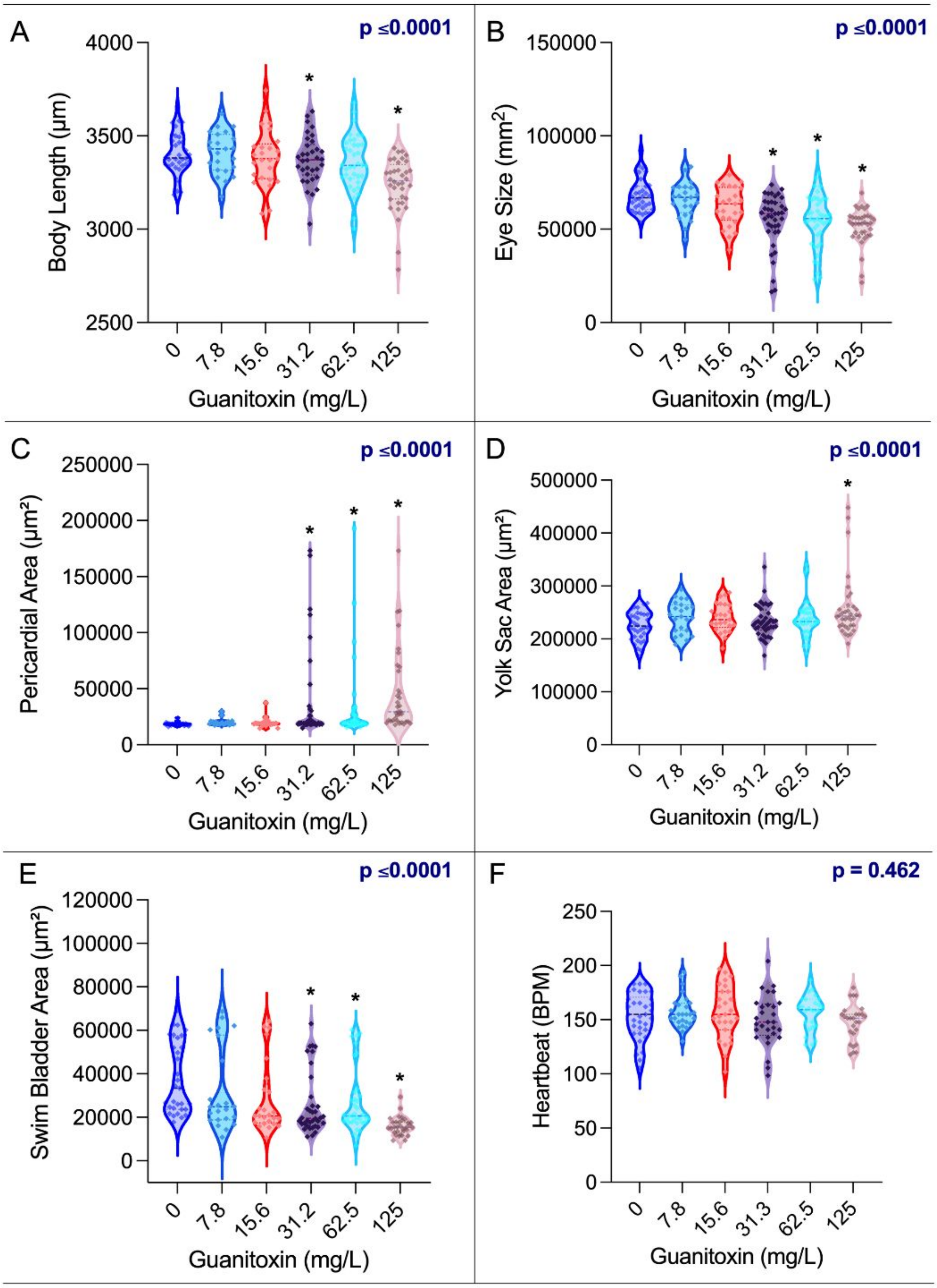

Figure S2. Body length (µm), eye size (mm^2), pericardial area ($µm^2$), yolk area ($µm^2$), swim bladder area ($µm^2$), and heartbeat (BPM) of *Danio rerio* larvae exposed to guanitoxin extracts for 120 hours. Asterisks (*) indicate statistically significant differences according to ANOVA and Dunnett post hoc tests.

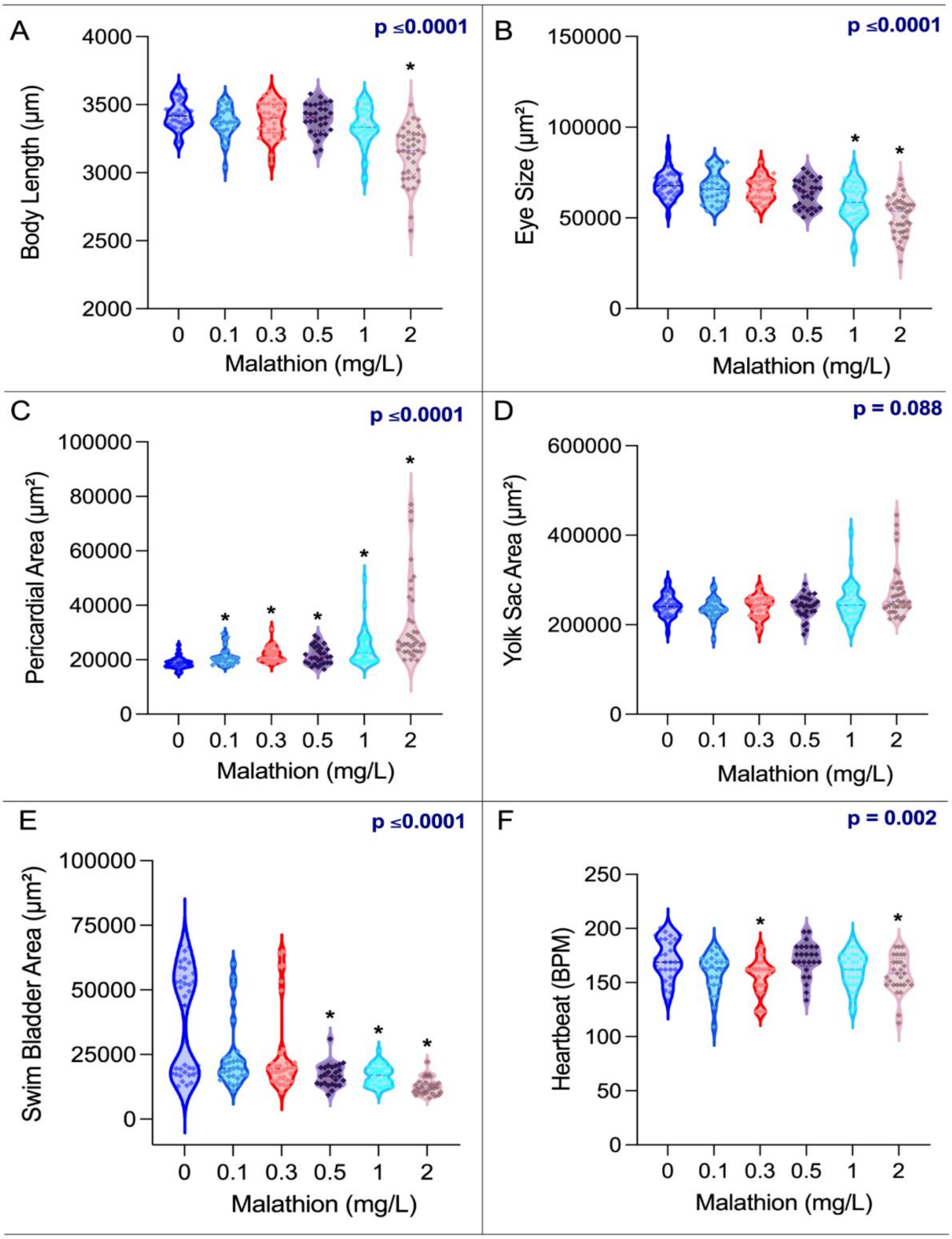

Figure S3. Body length (µm), eye size (mm^2), pericardial area (μm^2), yolk area (μm^2), swim bladder area (μm^2), and heartbeat (BPM) of *Danio rerio* larvae exposed to malathion for 120 hours. Asterisks (*) indicate statistically significant differences according to ANOVA and Dunnett post hoc tests.

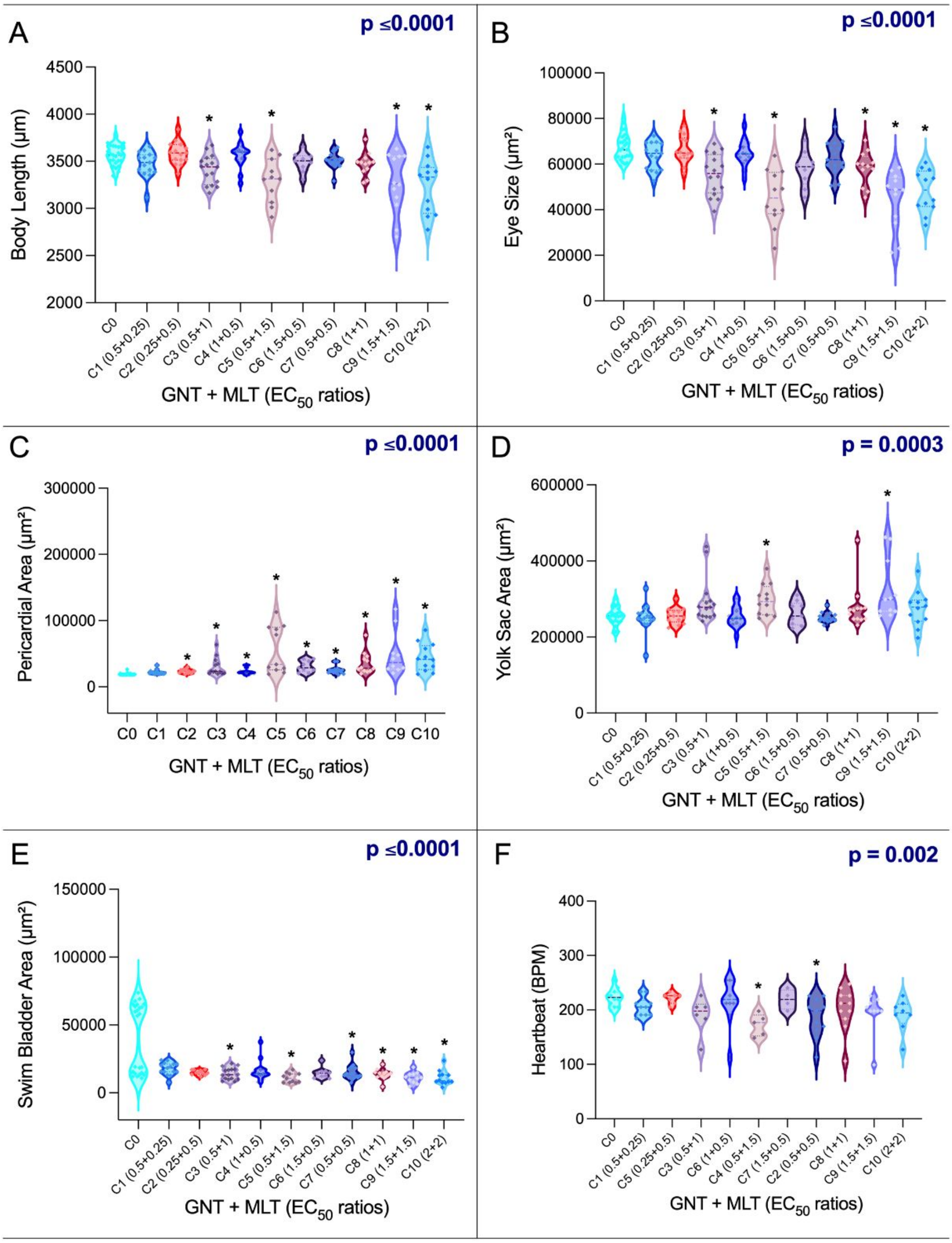

Figure S4. Body length (µm), eye size (mm^2), pericardial area (μm^2), yolk area (μm^2), swim bladder area (μm^2), and heartbeat (BPM) of *Danio rerio* larvae exposed to the binary mixture of guanitoxin + malathion for 120 hours. Asterisks (*) indicate statistically significant differences according to ANOVA and Dunnett post hoc tests.

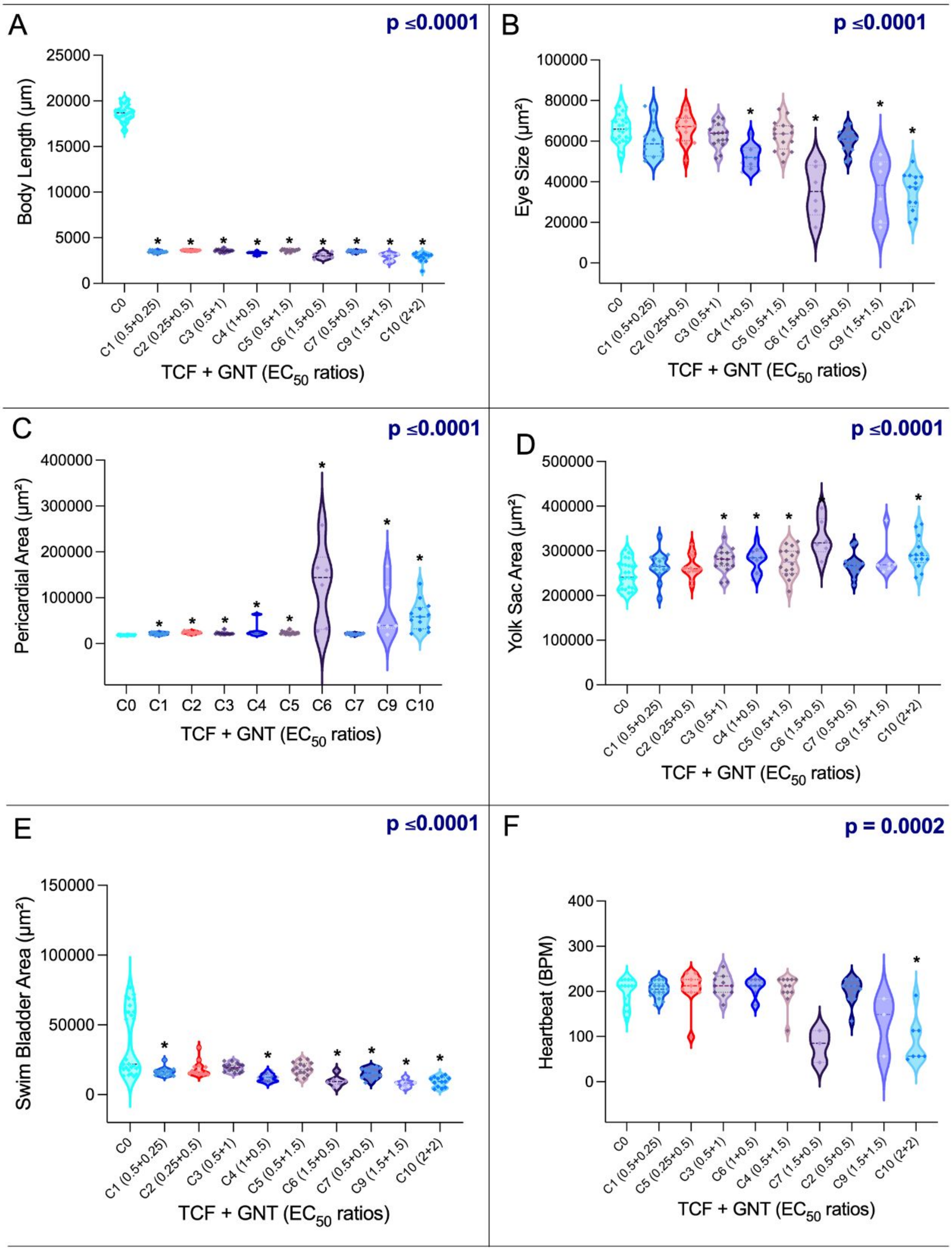

Figure S5. Body length (µm), eye size (mm^2), pericardial area (µm^2), yolk area (µm^2), swim bladder area (µm^2), and heartbeat (BPM) of *Danio rerio* larvae exposed to the binary mixture of trichlorfon + guanitoxin for 120 hours. Asterisks (*) indicate statistically significant differences according to ANOVA and Dunnett post hoc tests.

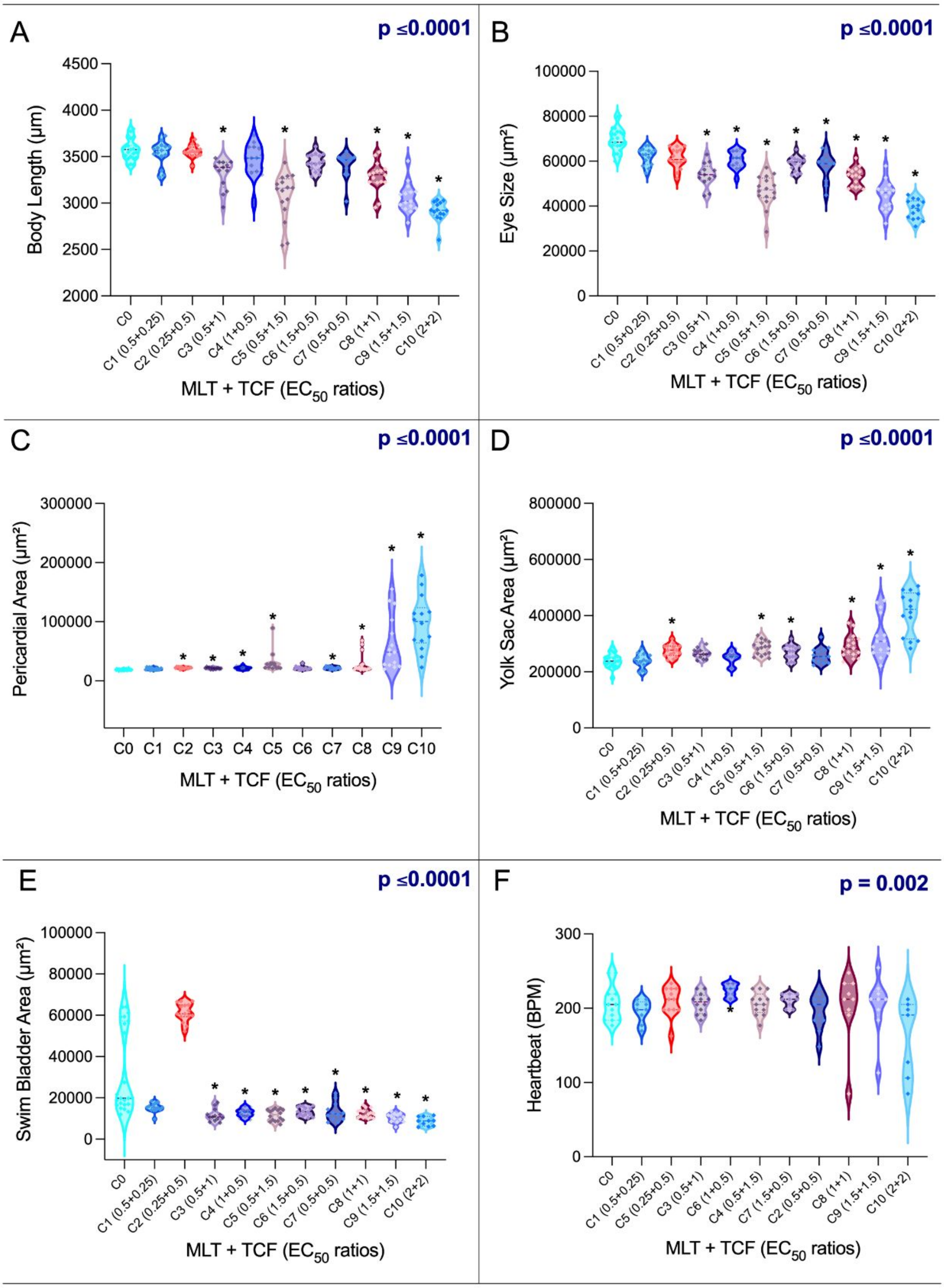

Figure S6. Body length (μm), eye size (mm^2), pericardial area (μm^2), yolk area (μm^2), swim bladder area (μm^2), and heartbeat (BPM) of *Danio rerio* larvae exposed to the binary mixture of malathion + trichlorfon for 120 hours. Asterisks (*) indicate statistically significant differences according to ANOVA and Dunnett post hoc tests.

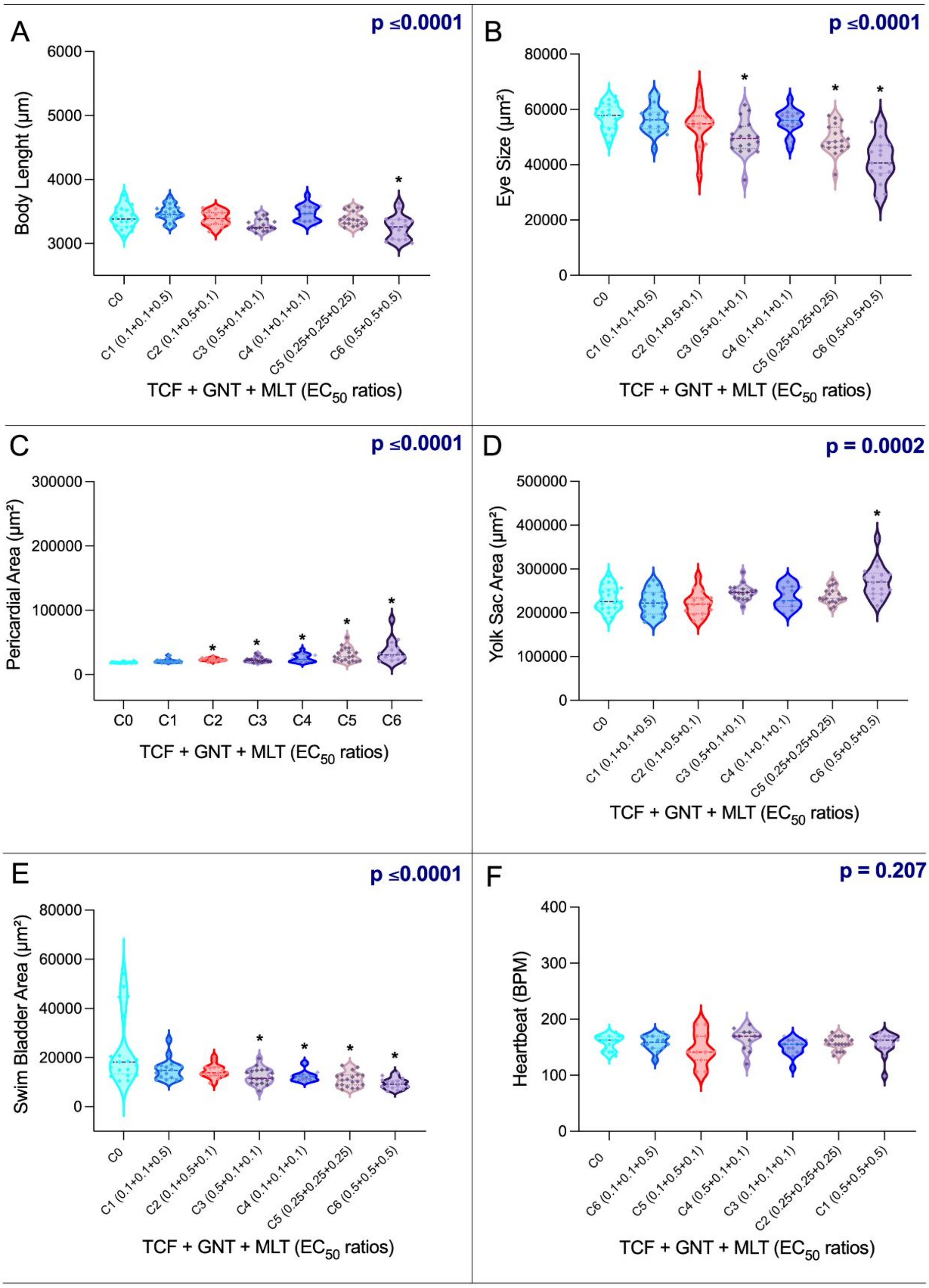

Figure S7. Body length (µm), eye size (mm^2), pericardial area (μm^2), yolk area (μm^2), swim bladder area (μm^2), and heartbeat (BPM) of *Danio rerio* larvae exposed to the tertiary mixture of trichlorfon + guanitoxin + malathion for 120 hours. Asterisks (*) indicate statistically significant differences according to ANOVA and Dunnett post hoc tests.

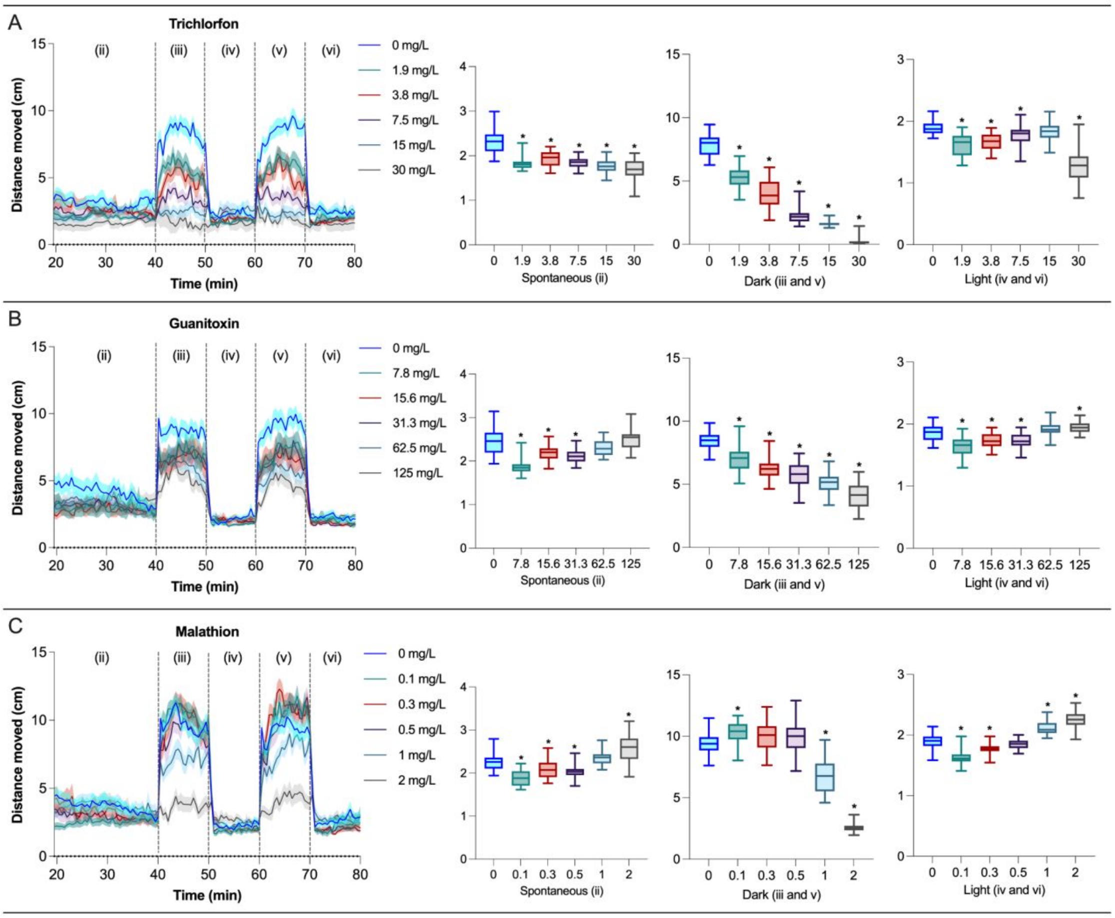

Figure S8. Time series illustrating the accumulated distance moved by *Danio rerio* larvae, recorded every 30 seconds, following 120 hours of exposure to varying concentrations of trichlorfon, guanitoxin extracts, and malathion. Boxplots represent the average distance moved during each phase of the behavior assay: dark intervals (III and IV) and light intervals (IV and VI). Asterisks (*) indicate statistically significant differences according to ANOVA and Dunnett post hoc tests.

Table S8. Effect size analysis of locomotor activity alterations in zebrafish larvae exposed to individual treatments (T1–T5) and binary or tertiary mixtures (C1–C10) of guanitoxin (GNT), malathion (MLT), and trichlorfon (TCF) during the dark phase. Effect sizes were calculated using Cohens d and expressed alongside the Common Language Effect Size (CLES), representing the probability that locomotor activity in exposed larvae differs from the control condition. Effect size magnitudes were classified according to conventional thresholds, with Cohen's d values greater than 1.3 interpreted as very large effects.

Malathion	*CLES*	Effect Size d_{Cohen}	Effect Size
T1	0.795	-1.165	Large
T2	0.716	-0.807	Large
T3	0.649	-0.543	Medium
T4	0.951	2.339	Very large
T5	1	10.563	Very large
Guanitoxin	*CLES*	Effect Size d_{Cohen}	Effect Size
T1	0.883	1.686	Very large
T2	0.982	2.967	Very large
T3	0.991	3.322	Very large
T4	0.999	4.274	Very large
T5	1	5.147	Very large
Trichlofon	*CLES*	Effect Size d_{Cohen}	Effect Size
T1	0.989	3.223	Very large
T2	1	4.945	Very large
T3	1	7.897	Very large
T4	1	8.672	Very large
T5	1	12.391	Very large
GNT + MLT	*CLES*	Effect Size d_{Cohen}	Effect Size
C1	0.933	2.12	Very large
C2	0.999	4.225	Very large
C3	0.979	2.886	Very large
C4	0.503	0.011	Small
C5	0.999	4.573	Very large
C6	0.997	3.908	Very large
C7	0.934	2.127	Very large
C8	0.957	2.428	Very large
C9	1	5.433	Very large
C10	1	5.815	Very large

TCF + MLT	*CLES*	Effect Size d_{Cohen}	Effect Size
C1	1	4.798	Very large
C2	1	5.614	Very large
C3	1	7.38	Very large
C4	1	10.062	Very large
C5	1	11.664	Very large
C6	1	5.42	Very large
C7	1	5.447	Very large
C8	1	11.529	Very large
C9	1	11.836	Very large
C10	1	7.622	Very large

GNT + TCF	*CLES*	Effect Size d_{Cohen}	Effect Size
C1	1	5.351	Very large
C2	0.998	4.035	Very large
C3	0.999	4.497	Very large
C4	1	7.293	Very large
C5	0.998	4.05	Very large
C6	1	8.518	Very large
C7	1	4.683	Very large
C9	0.999	4.607	Very large
C10	1	4.887	Very large

GNT + TCF + MLT	*CLES*	Effect Size d_{Cohen}	Effect Size
C1	0.999	4.607	Very large
C2	1	6.52	Very large
C3	1	12.041	Very large
C4	0.985	3.053	Very large
C5	1	11.524	Very large
C6	1	12.611	Very large

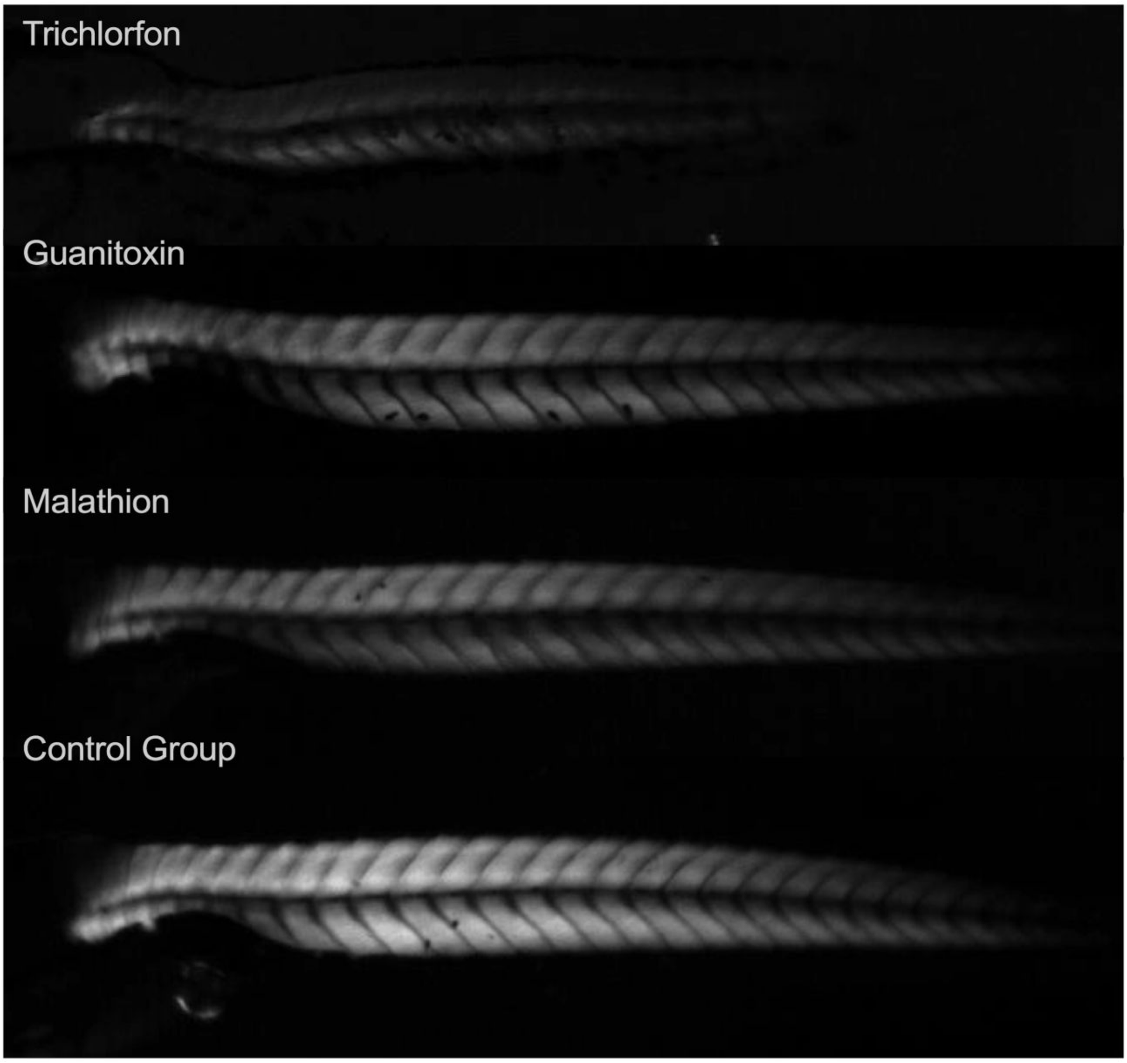

Figure S9. Representative birefringence images of zebrafish larvae from the control group and those exposed to trichlorfon, guanitoxin, and malathion.

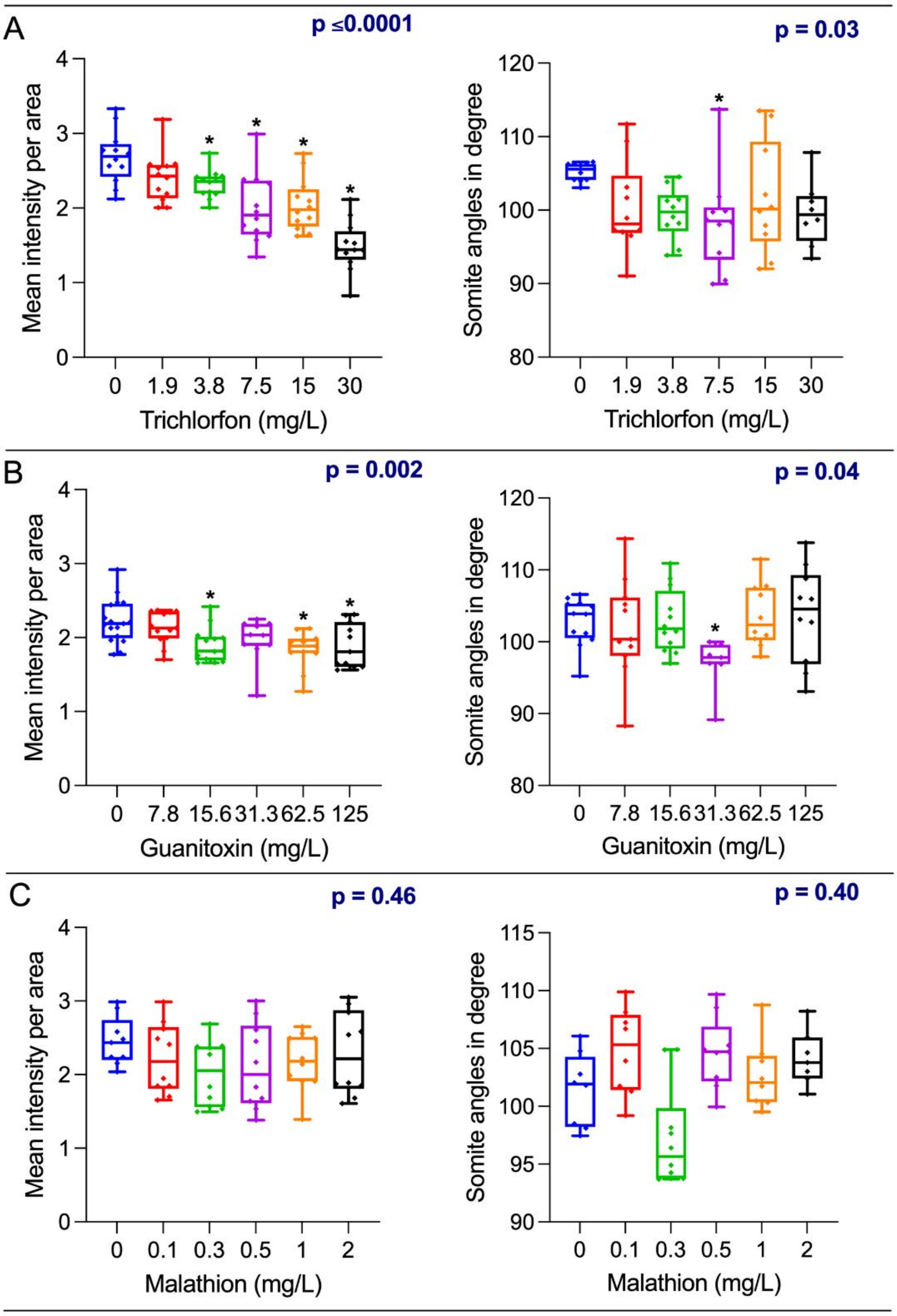

Figure S10. Average signal intensity per area (left panels) and somite angle in degrees (right panels) in *Danio rerio* larvae exposed to the individual concentrations of trichlorfon (A), guanitoxin extracts (B), and malathion (C), evaluated using the FIJI angle tool. For each treatment, 10 larvae were imaged and analyzed. Asterisks (*) denote statistically significant differences as determined by ANOVA followed by Dunnett's post hoc test.

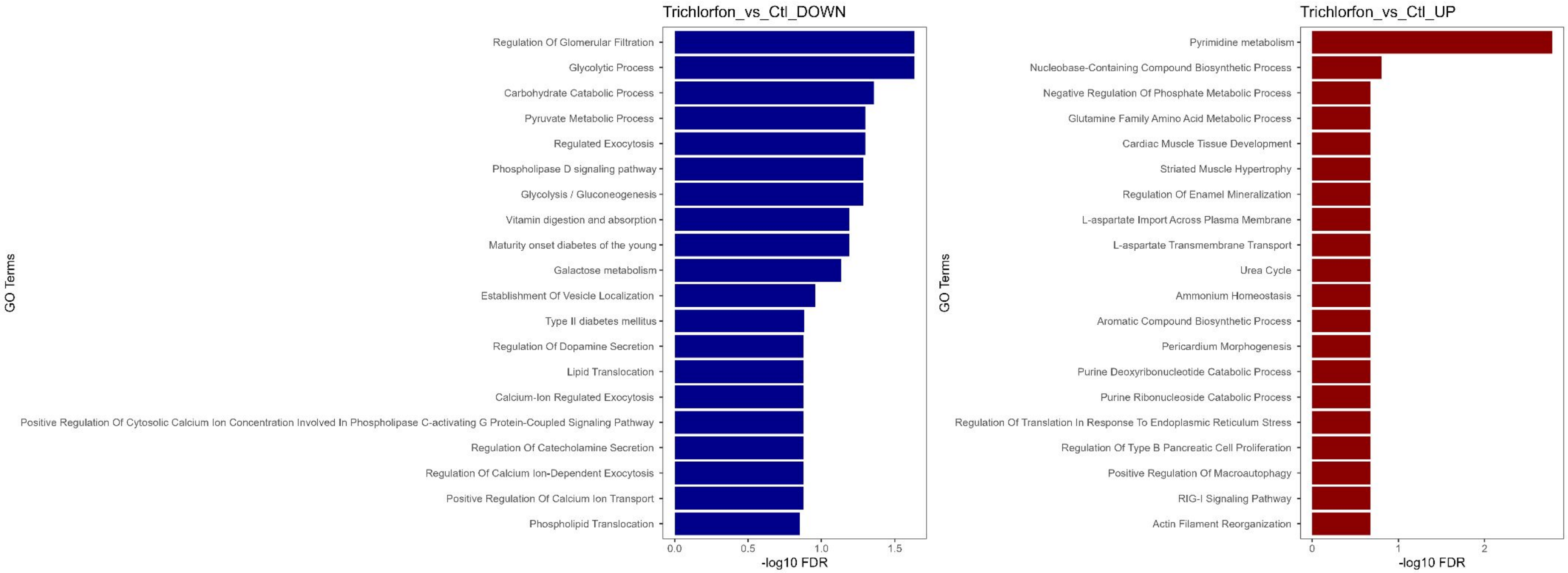

Figure S11. Enriched gene ontology (GO) terms in zebrafish larvae exposed to trichlorfon. The $-\log_{10}$(FDR) reflects the significance of each pathway. Pathways are displayed along the vertical axis, with red bars representing downregulated pathways and blue bars representing upregulated pathways.

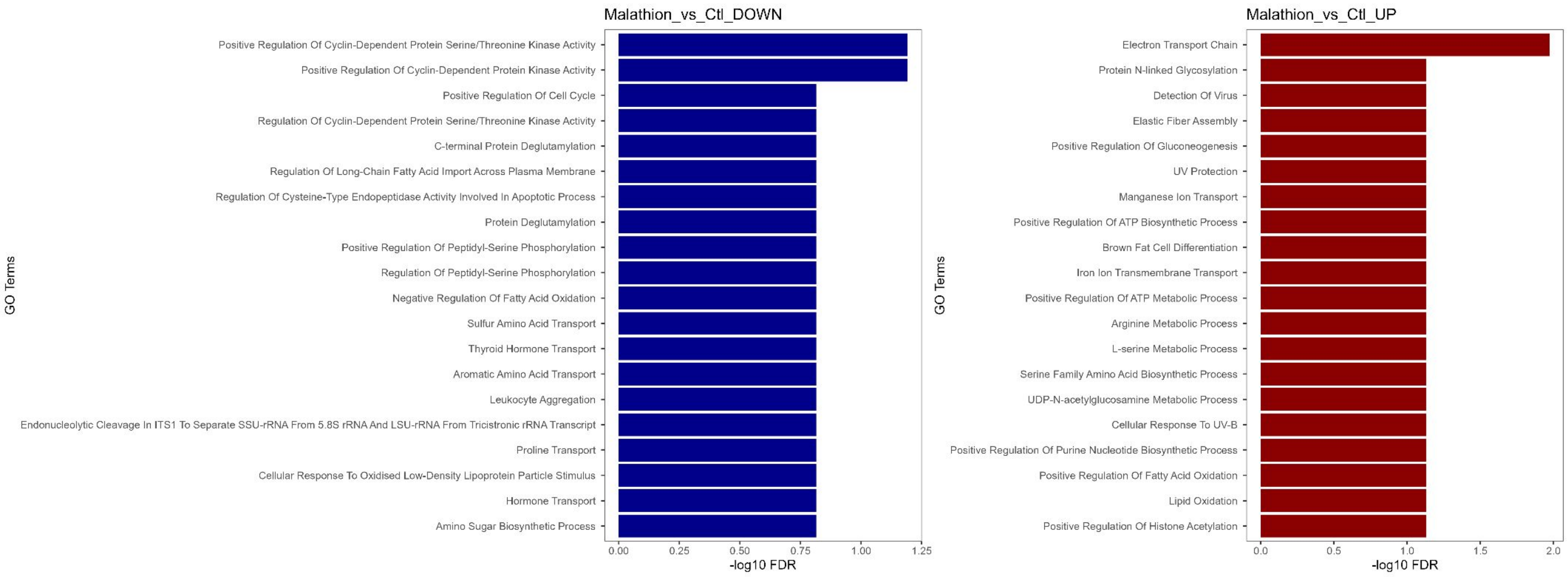

Figure S12. Enriched gene ontology (GO) terms in zebrafish larvae exposed to malathion. The –$\log_{10}$(FDR) reflects the significance of each pathway. Pathways are displayed along the vertical axis, with red bars representing downregulated pathways and blue bars representing upregulated pathways.

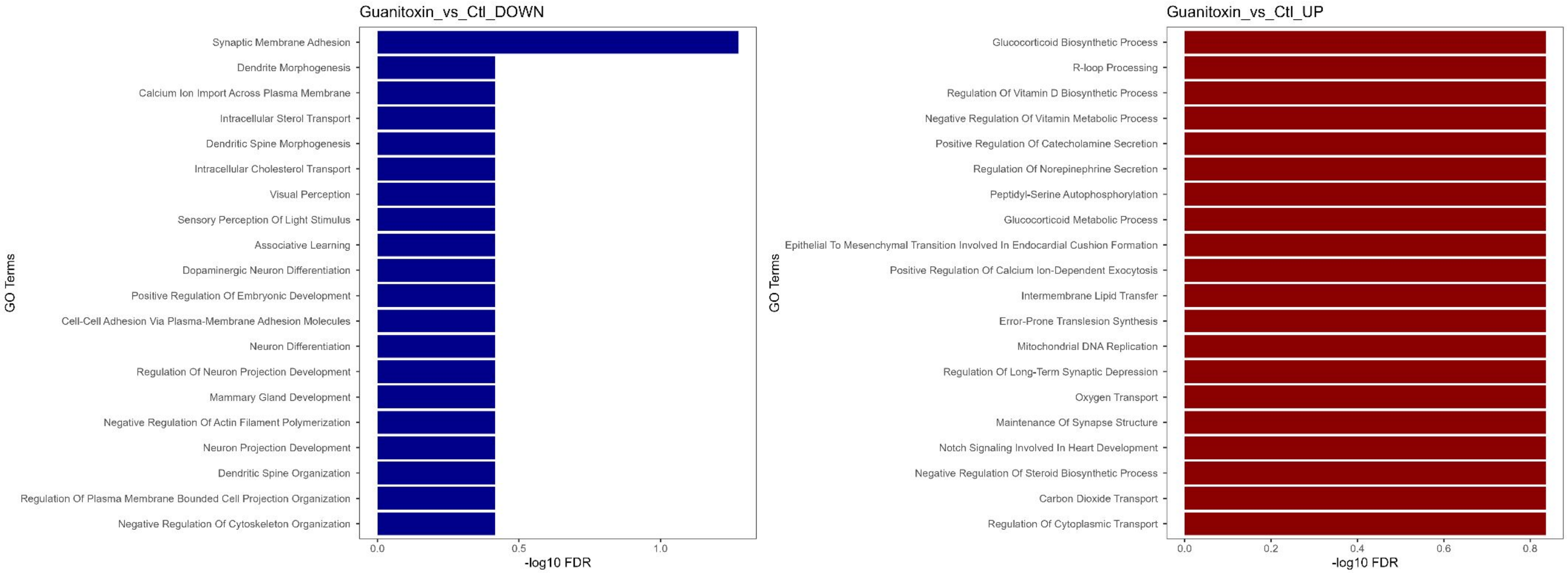

Figure S13. Enriched gene ontology (GO) terms in zebrafish larvae exposed to guanitoxin. The $-\log_{10}$(FDR) reflects the significance of each pathway. Pathways are displayed along the vertical axis, with red bars representing downregulated pathways and blue bars representing upregulated pathways.

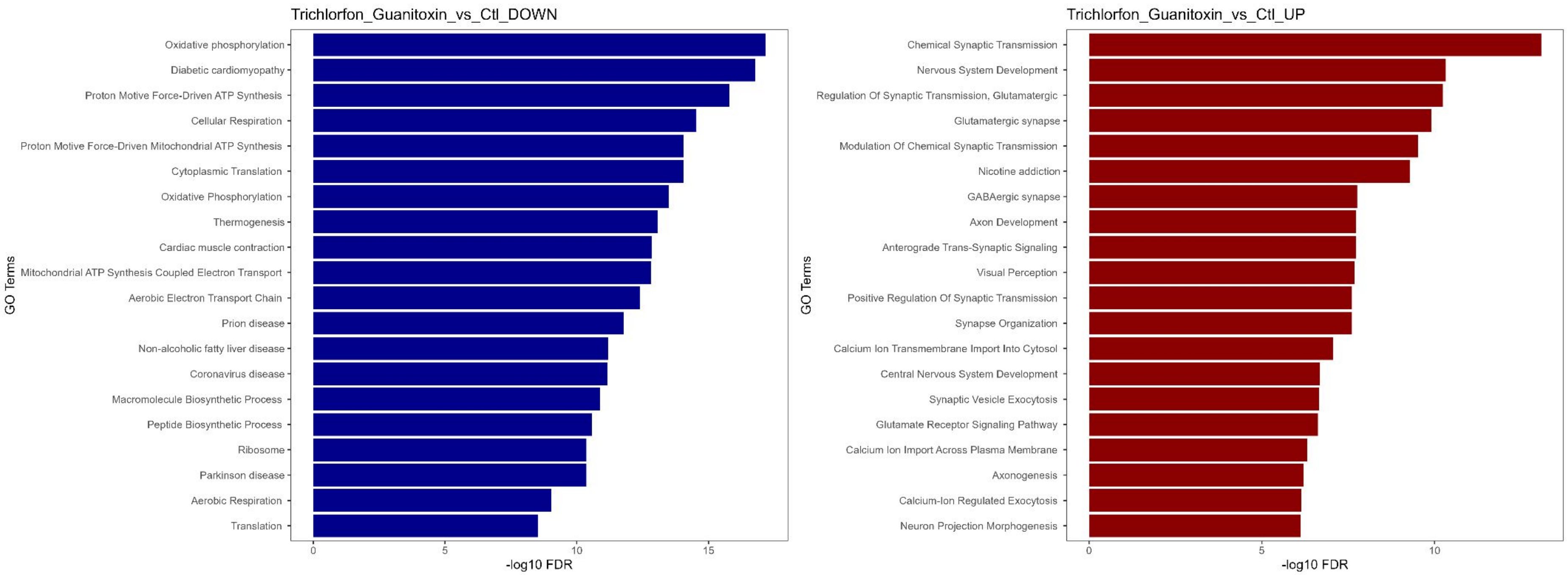

Figure S14. Enriched gene ontology (GO) terms in zebrafish larvae exposed to the trichlorfon + guanitoxin combination. The –$\log_{10}$(FDR) reflects the significance of each pathway. Pathways are displayed along the vertical axis, with red bars representing downregulated pathways and blue bars representing upregulated pathways.

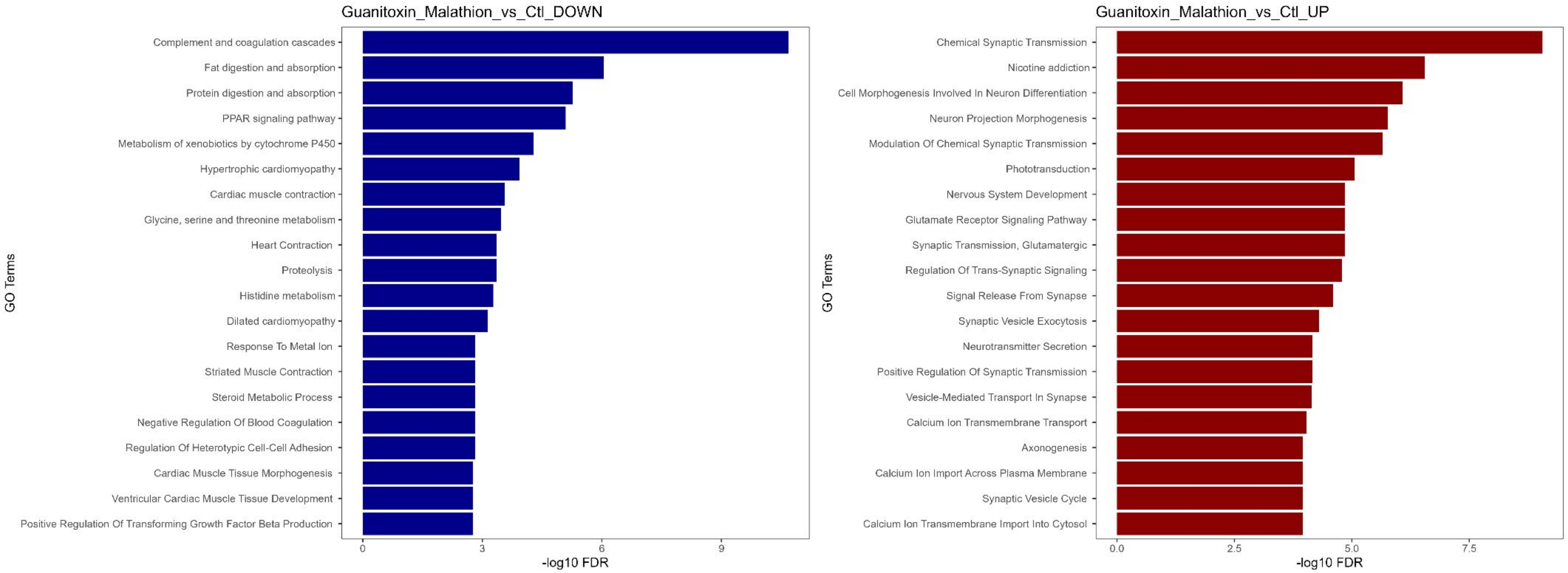

Figure S15. Enriched gene ontology (GO) terms in zebrafish larvae exposed to the guanitoxin + malathion combination. The $-\log_{10}$(FDR) reflects the significance of each pathway. Pathways are displayed along the vertical axis, with red bars representing downregulated pathways and blue bars representing upregulated pathways.

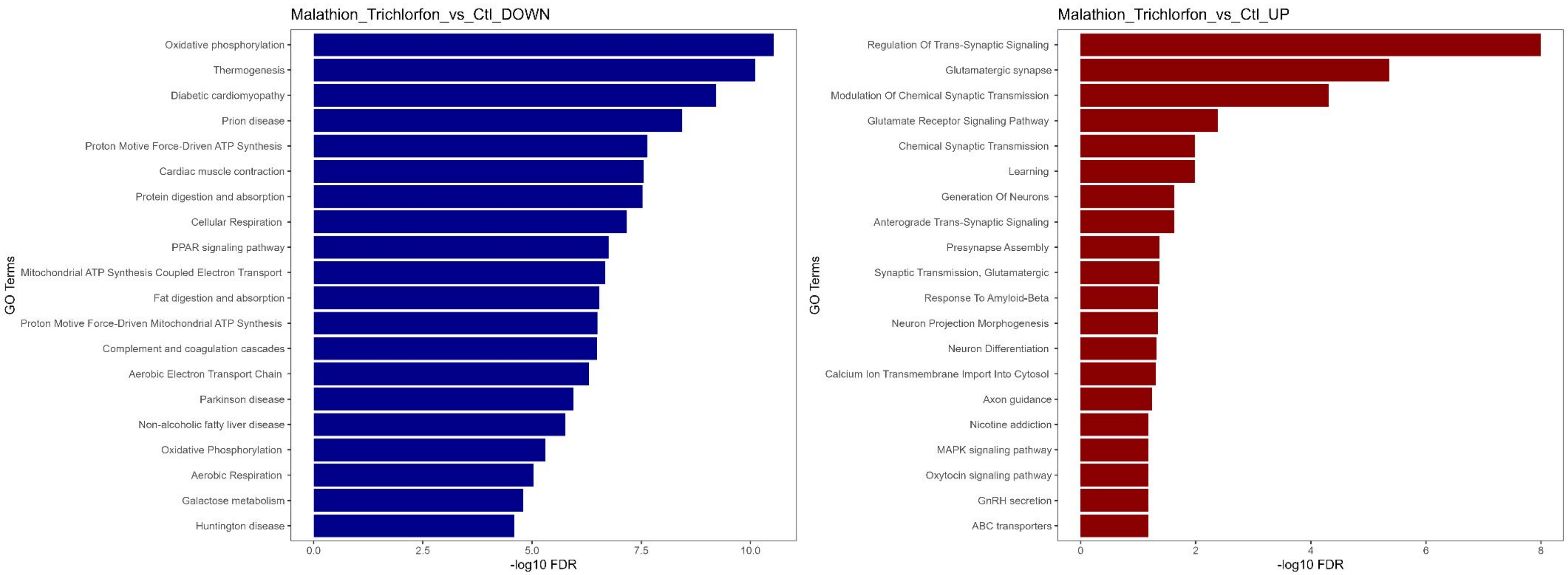

Figure S16. Enriched gene ontology (GO) terms in zebrafish larvae exposed to the malathion + trichlorfon combination. The $-\log_{10}$(FDR) reflects the significance of each pathway. Pathways are displayed along the vertical axis, with red bars representing downregulated pathways and blue bars representing upregulated pathways.

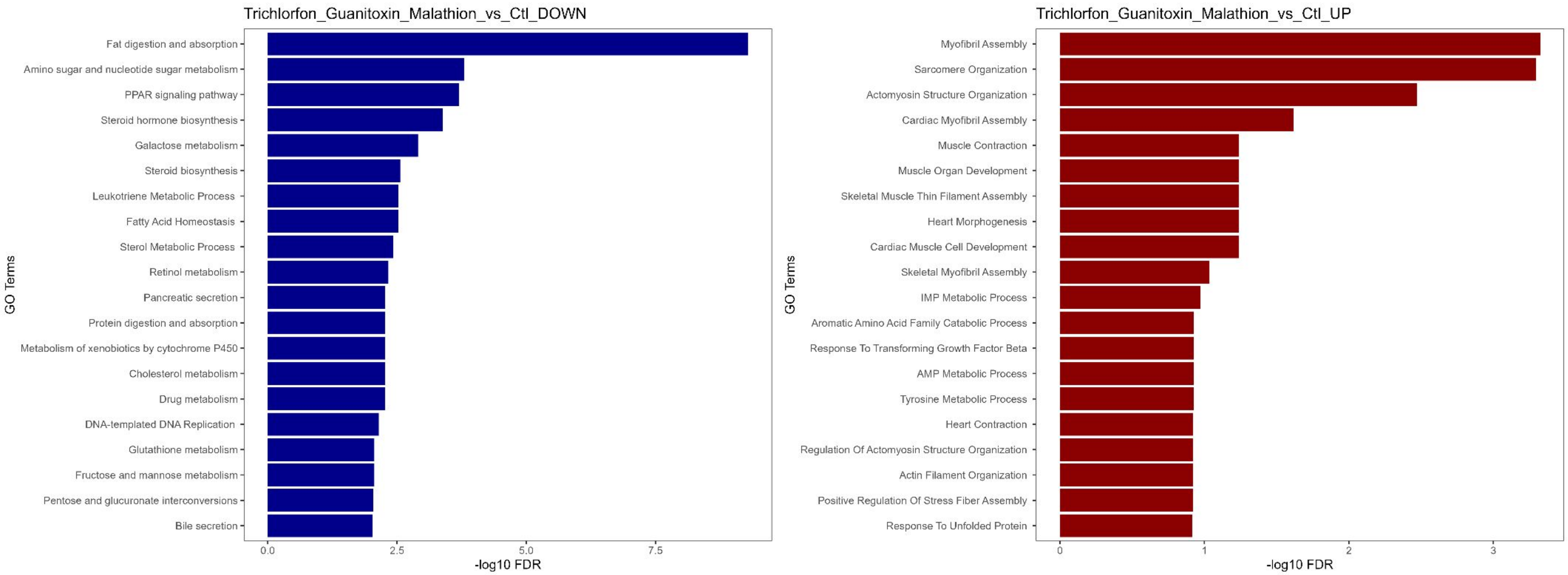

Figure S17. Enriched gene ontology (GO) terms in zebrafish larvae exposed to the trichlorfon + guanitoxin + malathion combination. The –$\log_{10}$(FDR) reflects the significance of each pathway. Pathways are displayed along the vertical axis, with red bars representing downregulated pathways and blue bars representing upregulated pathways.

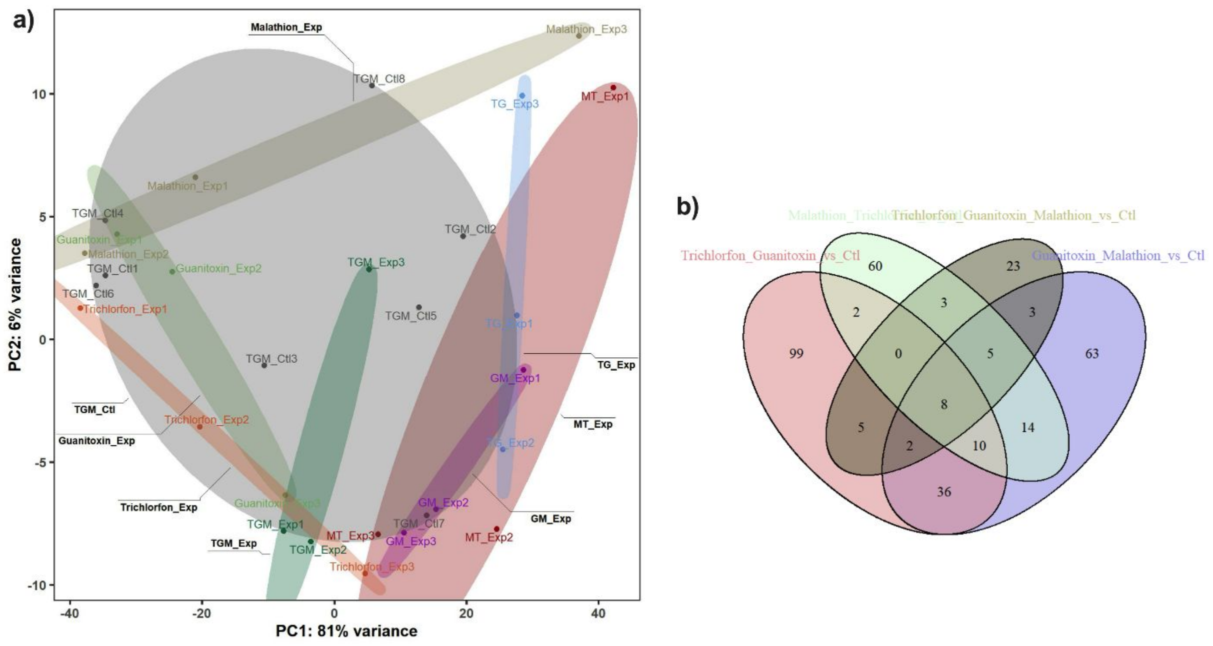

Figure S18. Principal component analysis (a) shows distinct transcriptomic profiles among treatments. Venn/intersection graphs (b) display shared and unique differentially expressed genes across mixtures.

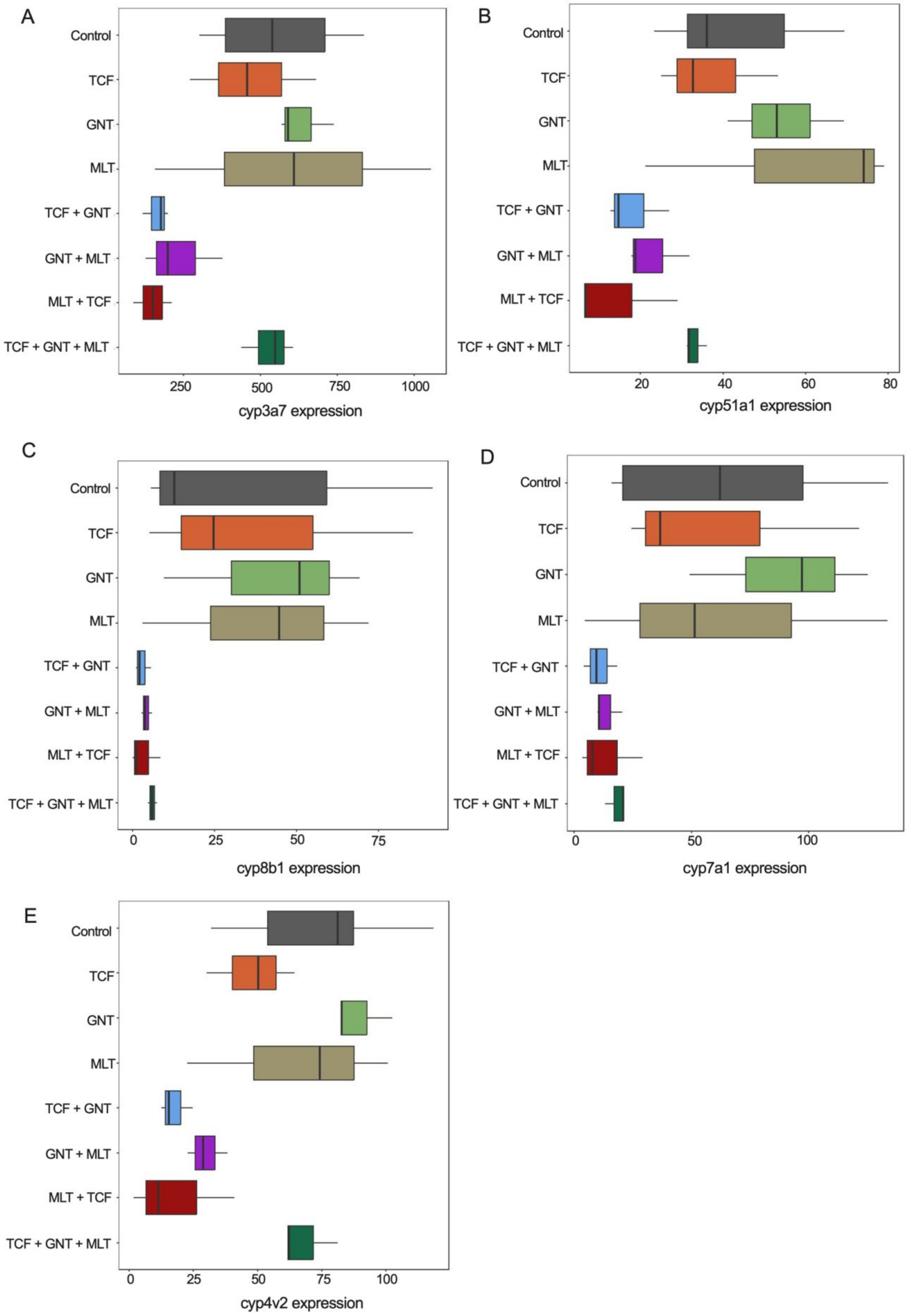

Figure S19. Transcriptomic expression patterns of *cyp* family genes in zebrafish exposed to trichlorfon (TCF), guanitoxin (GNT), and malathion (MLT), as well as to their binary (GNT + MLT, TCF + GNT, MLT + TCF) and tertiary (TCF + GNT + MLT) mixtures.

These plots provide a qualitative visualization of expression trends and were not used for statistical quantification or inference.

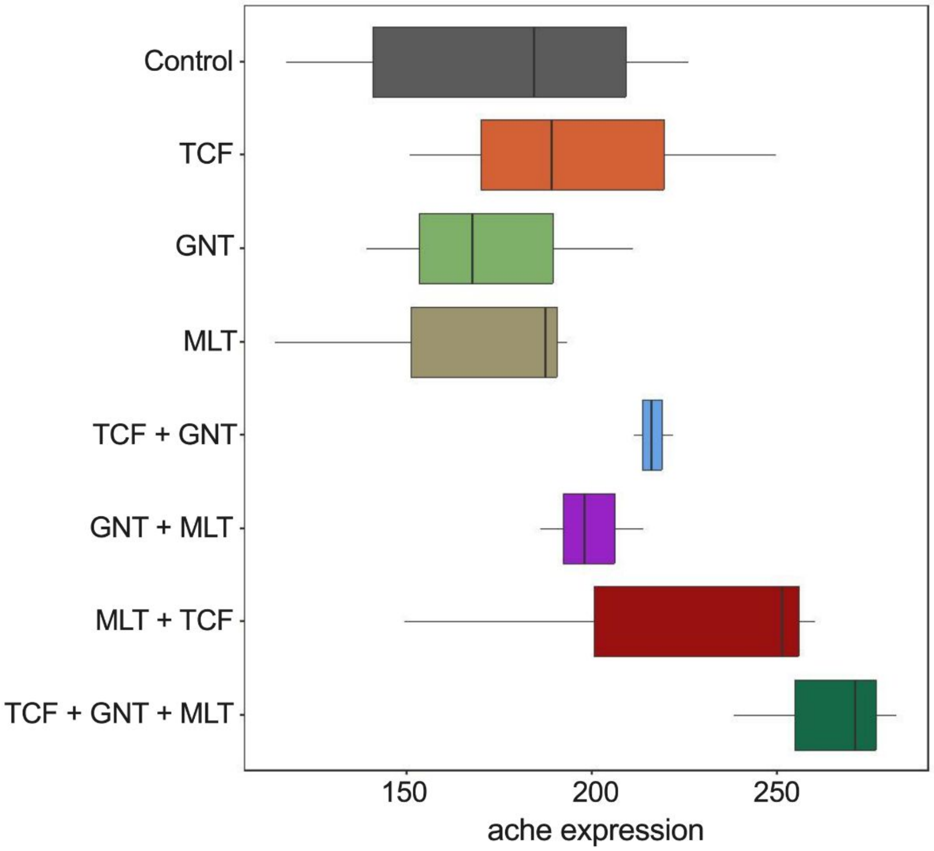

Figure S20. Transcriptomic expression patterns of *ache* gene in zebrafish exposed to trichlorfon (TCF), guanitoxin (GNT), and malathion (MLT), as well as to their binary (GNT + MLT, TCF + GNT, MLT + TCF) and tertiary (TCF + GNT + MLT) mixtures. These plots provide a qualitative visualization of expression trends and were not used for statistical quantification or inference.

Table S9. Effects of trichlorfon + guanitoxin (TCF + GNT) mixtures on biological parameters in zebrafish. The interaction type indicates whether the combined response is antagonistic, additive, or synergistic based on an empirical comparison between the mixture effect and the sum of individual compound effects. The empirical sum column represents the sum of the two individual values (TCF and GNT exposures), while the mixture column shows the values observed for the combined exposures. Synergism: observed mixture effect exceeds the sum of individual effects; Additive: difference < 10%; Antagonism: difference > 10–15%. The direction of the effect is considered for each parameter. Values represent up- or downregulation relative to control (%), with negative values indicating downregulation and positive values indicating upregulation.

Body lenght (% of control)						
Combinations	Ratios	TCF	GNT	Empirical sum	Mixture (TCF + GNT)	Interaction type
C1	0.5 + 0.25	-3,12	1,47	-1,65	-81,59	Synergism
C2	0.25 + 0.5	-1,22	1,47	0,25	-80,63	Synergism
C3	0.5 + 1	-3,12	-0,11	-3,23	-80,78	Synergism
C4	1 + 0.5	-5,08	1,47	-3,61	-82,14	Synergism
C5	0.5 + 1.5	-3,12	-0,11	-3,23	-80,38	Synergism
C6	1.5 + 0.5	-14,08	1,47	-12,61	-83,89	Synergism
C7	0.5 + 0.5	-3,12	1,47	-1,65	-81,24	Synergism
C9	1.5 + 1.5	-14,08	-0,11	-14,19	-83,66	Synergism
C10	2 + 2	-14,08	-1,23	-15,31	-83,74	Synergism
Eye size (% of control)						
Combinations	Ratios	TCF	GNT	Empirical sum	TCF + GNT	Interaction type
C1	0.5 + 0.25	-11,99	0,27	-11,72	-10,85	Additivity
C2	0.25 + 0.5	-9,26	0,27	-8,99	1,96	Antagonism
C3	0.5 + 1	-11,99	-4,89	-16,88	-3,01	Antagonism
C4	1 + 0.5	-18,78	0,27	-18,51	-21,4	Synergism
C5	0.5 + 1.5	-11,99	-4,89	-16,88	-3,48	Antagonism
C6	1.5 + 0.5	-32,71	0,27	-32,44	-46,59	Synergism
C7	0.5 + 0.5	-11,99	0,27	-11,72	-7,46	Antagonism
C9	1.5 + 1.5	-32,71	-4,89	-37,6	-41,93	Synergism
C10	2 + 2	-32,71	-12,7	-45,41	-43,16	Additivity
Swim bladder area (% of control)						
Combinations	Ratios	TCF	GNT	Empirical sum	TCF + GNT	Interaction type
C1	0.5 + 0.25	-40,44	-25,63	-66,07	-24,94	Antagonism
C2	0.25 + 0.5	-38,47	-25,63	-64,1	-27,03	Antagonism

C3	0.5 + 1	-40,44	-38,07	-78,51	-12,02	Antagonism
C4	1 + 0.5	-47,52	-25,63	-73,15	-43,79	Antagonism
C5	0.5 + 1.5	-40,44	-38,07	-78,51	-17,8	Antagonism
C6	1.5 + 0.5	-54,32	-25,63	-79,95	-57,34	Antagonism
C7	0.5 + 0.5	-40,44	-25,63	-66,07	-28,44	Antagonism
C9	1.5 + 1.5	-54,32	-38,07	-92,39	-62,1	Antagonism
C10	2 + 2	-54,32	-41,44	-95,76	-57,22	Antagonism

Yolk sac area (% of control)

Combinations	Ratios	TCF	GNT	Empirical sum	TCF + GNT	Interaction type
C1	0.5 + 0.25	6,91	7,99	14,9	0,56	Antagonism
C2	0.25 + 0.5	0,66	7,99	8,65	16,66	Synergism
C3	0.5 + 1	6,91	5,33	12,24	10,29	Antagonism
C4	1 + 0.5	10,25	7,99	18,24	6,72	Antagonism
C5	0.5 + 1.5	6,91	5,33	12,24	21,08	Synergism
C6	1.5 + 0.5	12,75	7,99	20,74	14,94	Antagonism
C7	0.5 + 0.5	6,91	7,99	14,9	7,19	Antagonism
C9	1.5 + 1.5	12,75	5,33	18,08	29,76	Synergism
C10	2 + 2	12,75	2,34	15,09	77,78	Synergism

Pericardial area (% of control)

Combinations	Ratios	TCF	GNT	Empirical sum	TCF + GNT	Interaction type
C1	0.5 + 0.25	2,23	4,67	6,9	13,02	Synergism
C2	0.25 + 0.5	4,12	4,67	8,79	29,08	Synergism
C3	0.5 + 1	5,64	2,96	8,6	15,57	Synergism
C4	1 + 0.5	4,12	4,67	8,79	27,76	Synergism
C5	0.5 + 1.5	43,82	2,96	46,78	24,19	Antagonism
C6	1.5 + 0.5	4,12	4,67	8,79	670,62	Synergism
C7	0.5 + 0.5	4,12	4,67	8,79	12,16	Synergism
C9	1.5 + 1.5	43,82	2,96	46,78	112,46	Synergism
C10	2 + 2	43,82	6,49	50,31	212,76	Synergism

Heartbeat (% of control)

Combinations	Ratios	TCF	GNT	Empirical sum	TCF + GNT	Interaction type
C1	0.5 + 0.25	3,98	-0,03	3,95	-3,3	Synergism
C2	0.25 + 0.5	-3,41	-0,03	-3,44	0,03	Antagonism
C3	0.5 + 1	3,98	-1,16	2,82	0,03	No interaction
C4	1 + 0.5	3,4	-0,03	3,37	0,03	No interaction
C5	0.5 + 1.5	3,98	-1,16	2,82	0,02	No interaction
C6	1.5 + 0.5	4,56	-0,03	4,53	-60,01	Synergism
C7	0.5 + 0.5	3,98	-0,03	3,95	-0,03	No interaction
C9	1.5 + 1.5	4,56	-1,16	3,4	-29,98	Synergism
C10	2 + 2	4,56	-5,71	-1,15	-73,33	Synergism

Locomotion dark (% of control)

Combinations	Ratios	TCF	GNT	Empirical sum	TCF + GNT	Interaction type
C1	0.5 + 0.25	-51,96	-16,48	-68,44	-59,93	Antagonism

C2	0.25 + 0.5	-33,86	-16,48	-50,34	-53,45	Additivity
C3	0.5 + 1	-51,96	-26,75	-78,71	-54	Antagonism
C4	1 + 0.5	-72,84	-16,48	-89,32	-77,28	Antagonism
C5	0.5 + 1.5	-51,96	-26,75	-78,71	-47,13	Antagonism
C6	1.5 + 0.5	-79,98	-16,48	-96,46	-84,11	Antagonism
C7	0.5 + 0.5	-51,96	-16,48	-68,44	-54,2	Antagonism
C9	1.5 + 1.5	-79,98	-26,75	-106,73	-76,1	Antagonism
C10	2 + 2	-79,98	-31,54	-111,52	-58,69	Antagonism
Muscle integrity (% of control)						
Combinations	Ratios	TCF	GNT	Empirical sum	TCF + GNT	Interaction type
C1	0.5 + 0.25	-11,77	-2,72	-14,49	-17,51	Antagonism
C2	0.25 + 0.5	-7,36	-2,72	-10,08	-18,81	Antagonism
C3	0.5 + 1	-11,77	-16,89	-28,66	-14,73	Antagonism
C4	1 + 0.5	-29,25	-2,72	-31,97	-16,98	Antagonism
C5	0.5 + 1.5	-11,77	-16,89	-28,66	-13,68	Antagonism
C6	1.5 + 0.5	-29,16	-2,72	-31,88	-10,64	Antagonism
C7	0.5 + 0.5	-11,77	-2,72	-14,49	-21,01	Antagonism
C9	1.5 + 1.5	-29,16	-16,89	-46,05	-15,4	Antagonism
C10	2 + 2	-29,16	-7,06	-36,22	-17,48	Antagonism
Somite angle (% of control)						
Combinations	Ratios	TCF	GNT	Empirical sum	TCF + GNT	Interaction type
C1	0.5 + 0.25	-5,52	-3,4	-8,92	4,22	Synergism
C2	0.25 + 0.5	-7,07	-3,4	-10,47	3,28	Synergism
C3	0.5 + 1	-5,52	-1,99	-7,51	1,03	Synergism
C4	1 + 0.5	-6,7	-3,4	-10,1	3,16	Synergism
C5	0.5 + 1.5	-5,52	-1,99	-7,51	3,56	Synergism
C6	1.5 + 0.5	-5,16	-3,4	-8,56	0,66	Synergism
C7	0.5 + 0.5	-5,52	-3,4	-8,92	1,44	Synergism
C9	1.5 + 1.5	-5,16	-1,99	-7,15	-0,01	No interaction
C10	2 + 2	-5,16	-5,85	-11,01	3,23	Synergism

Table S10. Effects of trichlorfon + guanitoxin (GNT + MLT) mixtures on biological parameters in zebrafish. The interaction type indicates whether the combined response is antagonistic, additive, or synergistic based on an empirical comparison between the mixture effect and the sum of individual compound effects. The empirical sum column represents the sum of the two individual values (GNT and MLT exposures), while the mixture column shows the values observed for the combined exposures. Synergism: observed mixture effect exceeds the sum of individual effects; Additive: difference < 10%; Antagonism: difference > 10–15%. The direction of the effect is considered for each parameter. Values represent up- or downregulation relative to control (%), with negative values indicating downregulation and positive values indicating upregulation.

Body lenght (% of control)						
Combinations	Ratios	GNT	MLT	Empirical sum	Mixture (GNT + MLT)	Interaction type
C1	0.5 + 0.25	1,47	-0,44	1,03	-2,21	Synergism
C2	0.25 + 0.5	1,47	-1,28	0,19	0,64	No interaction
C3	0.5 + 1	-0,11	-0,44	-0,55	-3,47	Synergism
C4	1 + 0.5	1,47	-0,36	1,11	0,89	No interaction
C5	0.5 + 1.5	-0,11	-0,44	-0,55	-7,14	Synergism
C6	1.5 + 0.5	1,47	-2,44	-0,97	-1,68	Synergism
C7	0.5 + 0.5	1,47	-0,44	1,03	-1,41	Synergism
C8	1 + 1	-0,11	-0,36	-0,47	-2,22	Synergism
C9	1.5 + 1.5	-0,11	-2,44	-2,55	-8,55	Synergism
C10	2 + 2	-1,23	-2,44	-3,67	-6,53	Synergism
Eye size (% of control)						
Combinations	Ratios	GNT	MLT	Empirical sum	GNT + MLT	Interaction type
C1	0.5 + 0.25	0,27	-3,51	-3,24	-2,4	Antagonism
C2	0.25 + 0.5	0,27	-3,11	-2,84	-2,11	Additivity
C3	0.5 + 1	-4,89	-3,51	-8,4	-15,73	Synergism
C4	1 + 0.5	0,27	-4,93	-4,66	-2,85	Antagonism
C5	0.5 + 1.5	-4,89	-3,51	-8,4	-31,92	Synergism
C6	1.5 + 0.5	0,27	-13,74	-13,47	-11,24	Antagonism
C7	0.5 + 0.5	0,27	-3,51	-3,24	-6,54	Synergism
C8	1 + 1	-4,89	-4,93	-9,82	-10,86	Additivity
C9	1.5 + 1.5	-4,89	-13,74	-18,63	-26,41	Synergism
C10	2 + 2	-12,7	-13,74	-26,44	-26,62	Additivity
Swim bladder area (% of control)						
Combinations	Ratios	GNT	MLT	Empirical sum	GNT + MLT	Interaction type

C1	0.5 + 0.25	-25,63	-55,56	-81,19	-45,81	Antagonism
C2	0.25 + 0.5	-25,63	-55,24	-80,87	-56,31	Antagonism
C3	0.5 + 1	-38,07	-55,56	-93,63	-60,61	Antagonism
C4	1 + 0.5	-25,63	-61,68	-87,31	-57,95	Antagonism
C5	0.5 + 1.5	-38,07	-55,56	-93,63	-68,64	Antagonism
C6	1.5 + 0.5	-25,63	-61,36	-86,99	-57,18	Antagonism
C7	0.5 + 0.5	-25,63	-55,56	-81,19	-58,75	Antagonism
C8	1 + 1	-38,07	-61,68	-99,75	-58,21	Antagonism
C9	1.5 + 1.5	-38,07	-61,36	-99,43	-66,74	Antagonism
C10	2 + 2	-41,44	-61,36	-102,8	-73,96	Antagonism

Yolk sac area (% of control)						
Combinations	Ratios	GNT	MLT	Empirical sum	GNT + MLT	Interaction type
C1	0.5 + 0.25	7,99	1,51	9,5	-2,36	Antagonism
C2	0.25 + 0.5	7,99	-2,75	5,24	-0,03	Antagonism
C3	0.5 + 1	5,33	1,51	6,84	8,96	Synergism
C4	1 + 0.5	7,99	0,33	8,32	-2,67	Antagonism
C5	0.5 + 1.5	5,33	1,51	6,84	14,7	Synergism
C6	1.5 + 0.5	7,99	1,1	9,09	-0,2	Antagonism
C7	0.5 + 0.5	7,99	1,51	9,5	-2,78	Antagonism
C8	1 + 1	5,33	0,33	5,66	5,64	Additivity
C9	1.5 + 1.5	5,33	1,1	6,43	16,81	Synergism
C10	2 + 2	2,34	1,1	3,44	9,06	Synergism

Pericardial area (% of control)						
Combinations	Ratios	GNT	MLT	Empirical sum	GNT + MLT	Interaction type
C1	0.5 + 0.25	4,67	15,58	20,25	13,87	Antagonism
C2	0.25 + 0.5	4,67	9,53	14,2	26,42	Synergism
C3	0.5 + 1	2,96	15,58	18,54	26,75	Synergism
C4	1 + 0.5	4,67	11,51	16,18	14,73	Additivity
C5	0.5 + 1.5	2,96	15,58	18,54	80,32	Synergism
C6	1.5 + 0.5	4,67	19,66	24,33	53,9	Synergism
C7	0.5 + 0.5	4,67	15,58	20,25	33,03	Synergism
C8	1 + 1	2,96	11,51	14,47	54,36	Synergism
C9	1.5 + 1.5	2,96	19,66	22,62	141,02	Synergism
C10	2 + 2	6,49	19,66	26,15	121,53	Synergism

Heartbeat (% of control)						
Combinations	Ratios	GNT	MLT	Empirical sum	GNT + MLT	Interaction type
C1	0.5 + 0.25	-0,03	-4,17	-4,2	-7,95	Synergism
C2	0.25 + 0.5	-0,03	-3,13	-3,16	1,54	Antagonism
C3	0.5 + 1	-1,16	-4,17	-5,33	-11,14	Synergism
C4	1 + 0.5	-0,03	4,16	4,13	-1,6	Synergism
C5	0.5 + 1.5	-1,16	-4,17	-5,33	-20,63	Synergism
C6	1.5 + 0.5	-0,03	-4,17	-4,2	-1,58	Antagonism
C7	0.5 + 0.5	-0,03	-4,17	-4,2	-11,12	Synergism

C8	1 + 1	-1,16	4,16	3	-4,75	Synergism
C9	1.5 + 1.5	-1,16	-4,17	-5,33	-11,12	Synergism
C10	2 + 2	-5,71	-4,17	-9,88	-12,7	Synergism
Locomotion dark (% of control)						
Combinations	Ratios	GNT	MLT	Empirical sum	GNT + MLT	Interaction type
C1	0.5 + 0.25	-16,48	7,44	-9,04	-18,33	Synergism
C2	0.25 + 0.5	-16,48	10,73	-5,75	-34,97	Synergism
C3	0.5 + 1	-26,75	7,44	-19,31	-36,29	Synergism
C4	1 + 0.5	-16,48	6,48	-10	-0,11	Antagonism
C5	0.5 + 1.5	-26,75	7,44	-19,31	-41,64	Synergism
C6	1.5 + 0.5	-16,48	-27,92	-44,4	-35,58	Antagonism
C7	0.5 + 0.5	-16,48	7,44	-9,04	-18,17	Synergism
C8	1 + 1	-26,75	6,48	-20,27	-27,95	Synergism
C9	1.5 + 1.5	-26,75	-27,92	-54,67	-79,63	Synergism
C10	2 + 2	-31,54	-27,92	-59,46	-49,69	Antagonism
Muscle integrity (% of control)						
Combinations	Ratios	GNT	MLT	Empirical sum	GNT + MLT	Interaction type
C1	0.5 + 0.25	-2,72	-15,67	-18,39	-0,2	Antagonism
C2	0.25 + 0.5	-2,72	-10,51	-13,23	5,02	Antagonism
C3	0.5 + 1	-16,89	-15,67	-32,56	7,01	Antagonism
C4	1 + 0.5	-2,72	-17,85	-20,57	-4,28	Antagonism
C5	0.5 + 1.5	-16,89	-15,67	-32,56	4,76	Antagonism
C6	1.5 + 0.5	-2,72	-10,45	-13,17	9,79	Antagonism
C7	0.5 + 0.5	-2,72	-15,67	-18,39	2,24	Antagonism
C8	1 + 1	-16,89	-17,85	-34,74	2,01	Antagonism
C9	1.5 + 1.5	-16,89	-10,45	-27,34	3,97	Antagonism
C10	2 + 2	-7,06	-10,45	-17,51	2,01	Antagonism
Somite angle (% of control)						
Combinations	Ratios	GNT	MLT	Empirical sum	GNT + MLT	Interaction type
C1	0.5 + 0.25	-3,4	-6,14	-9,54	-0,16	No interaction
C2	0.25 + 0.5	-3,4	3,33	-0,07	1,04	Synergism
C3	0.5 + 1	-1,99	-6,14	-8,13	3,24	Synergism
C4	1 + 0.5	-3,4	2,75	-0,65	-3,6	No interaction
C5	0.5 + 1.5	-1,99	-6,14	-8,13	-0,07	No interaction
C6	1.5 + 0.5	-3,4	0,13	-3,27	-0,94	No interaction
C7	0.5 + 0.5	-3,4	-6,14	-9,54	-4,86	No interaction
C8	1 + 1	-1,99	2,75	0,76	0,16	Antagonism
C9	1.5 + 1.5	-1,99	0,13	-1,86	0,28	Synergism
C10	2 + 2	-5,85	0,13	-5,72	0,04	No interaction

Table S11. Effects of trichlorfon + guanitoxin (MLT + TCF) mixtures on biological parameters in zebrafish. The interaction type indicates whether the combined response is antagonistic, additive, or synergistic based on an empirical comparison between the mixture effect and the sum of individual compound effects. The empirical sum column represents the sum of the two individual values (MLT and TCF exposures), while the mixture column shows the values observed for the combined exposures. Synergism: observed mixture effect exceeds the sum of individual effects; Additive: difference < 10%; Antagonism: difference > 10–15%. The direction of the effect is considered for each parameter. Values represent up- or downregulation relative to control (%), with negative values indicating downregulation and positive values indicating upregulation.

Body lenght (% of control)						
Combinations	Ratios	MLT	TCF	Empirical sum	Mixture (MLT + TCF)	Interaction type
C1	0.5 + 0.25	-0,44	-3,12	-3,56	-0,24	Antagonism
C2	0.25 + 0.5	-1,28	-1,22	-2,5	-0,82	Antagonism
C3	0.5 + 1	-0,44	-3,12	-3,56	-5,35	Synergism
C4	1 + 0.5	-0,36	-5,08	-5,44	-2,63	Antagonism
C5	0.5 + 1.5	-0,44	-3,12	-3,56	-11,69	Synergism
C6	1.5 + 0.5	-2,44	-14,08	-16,52	-4,17	Antagonism
C7	0.5 + 0.5	-0,44	-3,12	-3,56	-3,32	Additivity
C8	1 + 1	-0,36	-5,08	-5,44	-7,6	Synergism
C9	1.5 + 1.5	-2,44	-14,08	-16,52	-15,55	Additivity
C10	2 + 2	-2,44	-14,08	-16,52	-18,32	Synergism

Eye size (% of control)						
Combinations	Ratios	MLT	TCF	Empirical sum	MLT + TCF	Interaction type
C1	0.5 + 0.25	-3,51	-11,99	-15,5	-8,01	Antagonism
C2	0.25 + 0.5	-3,11	-9,26	-12,37	-11,32	Additivity
C3	0.5 + 1	-3,51	-11,99	-15,5	-21,19	Synergism
C4	1 + 0.5	-4,93	-18,78	-23,71	-11,94	Antagonism
C5	0.5 + 1.5	-3,51	-11,99	-15,5	-31,02	Synergism
C6	1.5 + 0.5	-13,74	-32,71	-46,45	-12,92	Antagonism
C7	0.5 + 0.5	-3,51	-11,99	-15,5	-14,08	Additivity
C8	1 + 1	-4,93	-18,78	-23,71	-21,81	Additivity
C9	1.5 + 1.5	-13,74	-32,71	-46,45	-32,83	Antagonism
C10	2 + 2	-13,74	-32,71	-46,45	-42,95	Additivity

Swim bladder area (% of control)						
Combinations	Ratios	MLT	TCF	Empirical sum	MLT + TCF	Interaction type

C1	0.5 + 0.25	-55,56	-40,44	-96	-22,19	Antagonism
C2	0.25 + 0.5	-55,24	-38,47	-93,71	206,3	No interaction
C3	0.5 + 1	-55,56	-40,44	-96	-45,57	Antagonism
C4	1 + 0.5	-61,68	-47,52	-109,2	-33,67	Antagonism
C5	0.5 + 1.5	-55,56	-40,44	-96	-40,09	Antagonism
C6	1.5 + 0.5	-61,36	-54,32	-115,68	-32,86	Antagonism
C7	0.5 + 0.5	-55,56	-40,44	-96	-37,84	Antagonism
C8	1 + 1	-61,68	-47,52	-109,2	-38,34	Antagonism
C9	1.5 + 1.5	-61,36	-54,32	-115,68	-46,38	Antagonism
C10	2 + 2	-61,36	-54,32	-115,68	-55,17	Antagonism
Yolk sac area (% of control)						
Combinations	Ratios	MLT	TCF	Empirical sum	MLT + TCF	Interaction type
C1	0.5 + 0.25	1,51	6,91	8,42	0,56	Antagonism
C2	0.25 + 0.5	-2,75	0,66	-2,09	16,66	Synergism
C3	0.5 + 1	1,51	6,91	8,42	10,29	Synergism
C4	1 + 0.5	0,33	10,25	10,58	6,72	Antagonism
C5	0.5 + 1.5	1,51	6,91	8,42	21,08	Synergism
C6	1.5 + 0.5	1,1	12,75	13,85	14,94	Additivity
C7	0.5 + 0.5	1,51	6,91	8,42	7,19	Antagonism
C8	1 + 1	0,33	10,25	10,58	18,69	Synergism
C9	1.5 + 1.5	1,1	12,75	13,85	29,76	Synergism
C10	2 + 2	1,1	12,75	13,85	77,78	Synergism
Pericardial area (% of control)						
Combinations	Ratios	MLT	TCF	Empirical sum	MLT + TCF	Interaction type
C1	0.5 + 0.25	15,58	2,23	17,81	4,55	Antagonism
C2	0.25 + 0.5	9,53	4,12	13,65	18,02	Synergism
C3	0.5 + 1	15,58	5,64	21,22	14,06	Antagonism
C4	1 + 0.5	11,51	4,12	15,63	18,35	Synergism
C5	0.5 + 1.5	15,58	43,82	59,4	48,88	Antagonism
C6	1.5 + 0.5	19,66	4,12	23,78	11,98	Antagonism
C7	0.5 + 0.5	15,58	4,12	19,7	18,48	Additivity
C8	1 + 1	11,51	5,64	17,15	13,96	Antagonism
C9	1.5 + 1.5	19,66	43,82	63,48	158,48	Synergism
C10	2 + 2	19,66	43,82	63,48	435,71	Synergism
Heartbeat (% of control)						
Combinations	Ratios	MLT	TCF	Empirical sum	MLT + TCF	Interaction type
C1	0.5 + 0.25	-4,17	3,98	-0,19	-3,45	Synergism
C2	0.25 + 0.5	-3,13	-3,41	-6,54	3,43	Antagonism
C3	0.5 + 1	-4,17	3,98	-0,19	1,7	Antagonism
C4	1 + 0.5	4,16	3,4	7,56	10,34	No interaction
C5	0.5 + 1.5	-4,17	3,98	-0,19	0	No interaction
C6	1.5 + 0.5	-4,17	4,56	0,39	3,41	No interaction
C7	0.5 + 0.5	-4,17	3,98	-0,19	-0,04	No interaction

C8	1 + 1	4,16	3,4	7,56	3,45	No interaction
C9	1.5 + 1.5	-4,17	4,56	0,39	3,43	No interaction
C10	2 + 2	-4,17	4,56	0,39	-6,91	Synergism
Locomotion dark (% of control)						
Combinations	Ratios	MLT	TCF	Empirical sum	MLT + TCF	Interaction type
C1	0.5 + 0.25	7,44	-51,96	-44,52	-45,83	Additivity
C2	0.25 + 0.5	10,73	-33,86	-23,13	-53,63	Synergism
C3	0.5 + 1	7,44	-51,96	-44,52	-87,74	Synergism
C4	1 + 0.5	6,48	-72,84	-66,36	-75,12	Synergism
C5	0.5 + 1.5	7,44	-51,96	-44,52	-85,87	Synergism
C6	1.5 + 0.5	-27,92	-79,98	-107,9	-70,4	Antagonism
C7	0.5 + 0.5	7,44	-51,96	-44,52	-70,75	Synergism
C8	1 + 1	6,48	-72,84	-66,36	-84,93	Synergism
C9	1.5 + 1.5	-27,92	-79,98	-107,9	-87,19	Antagonism
C10	2 + 2	-27,92	-79,98	-107,9	-72,16	Antagonism
Muscle integrity (% of control)						
Combinations	Ratios	MLT	TCF	Empirical sum	MLT + TCF	Interaction type
C1	0.5 + 0.25	-15,67	-11,77	-27,44	-21,08	Antagonism
C2	0.25 + 0.5	-10,51	-7,36	-17,87	-16,91	Additivity
C3	0.5 + 1	-15,67	-11,77	-27,44	-39,86	Synergism
C4	1 + 0.5	-17,85	-29,25	-47,1	-12,14	Antagonism
C5	0.5 + 1.5	-15,67	-11,77	-27,44	-20,94	Antagonism
C6	1.5 + 0.5	-10,45	-29,16	-39,61	-46,96	Synergism
C7	0.5 + 0.5	-15,67	-11,77	-27,44	-15,31	Antagonism
C8	1 + 1	-17,85	-29,25	-47,1	-43,03	Additivity
C9	1.5 + 1.5	-10,45	-29,16	-39,61	-35,58	Additivity
C10	2 + 2	-10,45	-29,16	-39,61	-43,22	Additivity
Somite angle (% of control)						
Combinations	Ratios	MLT	TCF	Empirical sum	MLT + TCF	Interaction type
C1	0.5 + 0.25	-6,14	-5,52	-11,66	5,75	Synergism
C2	0.25 + 0.5	3,33	-7,07	-3,74	3,27	Synergism
C3	0.5 + 1	-6,14	-5,52	-11,66	6,46	Synergism
C4	1 + 0.5	2,75	-6,7	-3,95	5,98	Synergism
C5	0.5 + 1.5	-6,14	-5,52	-11,66	4,28	Synergism
C6	1.5 + 0.5	0,13	-5,16	-5,03	3,98	Synergism
C7	0.5 + 0.5	-6,14	-5,52	-11,66	3,76	Synergism
C8	1 + 1	2,75	-6,7	-3,95	5,67	Synergism
C9	1.5 + 1.5	0,13	-5,16	-5,03	6,07	Synergism
C10	2 + 2	0,13	-5,16	-5,03	4,07	Synergism

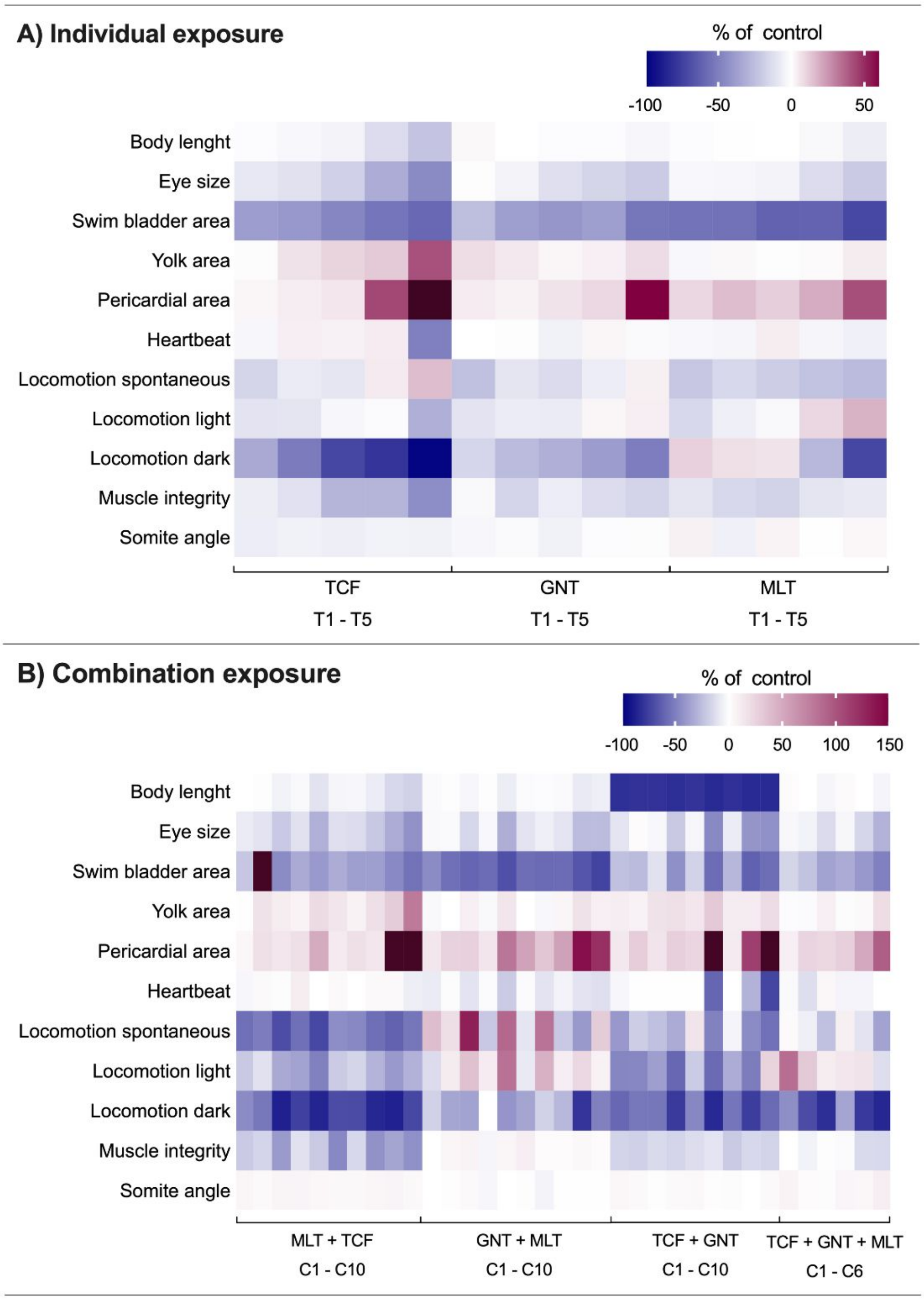

Figure S21. Heatmap of endpoints in zebrafish after 120 hours exposure to guanitoxin (GNT), malathion (MLT), and trichlorfon (TCF), individually (T1-T5, expressed in mg/L; see also Table S2) (A) and in combination (C1-C10, ratios based on the EC_{50}, expressed in mg/L; see also Table S3) (B). Data are presented as percentage relative to control (normalized). Blue indicates downregulation and maroon indicates upregulation.
